# Supplementary material for: A cluster randomised controlled trial to evaluate the effectiveness and cost-effectiveness of the GoActive intervention to increase physical activity among adolescents aged 13–14 years
Source: BMJ Open. 2017 Sep 27;7(9):e014419. doi: 10.1136/bmjopen-2016-014419 (PMC5623411; doi:10.1136/bmjopen-2016-014419)
Supplement: Supplementary data 1 [file bmjopen-2016-014419supp001.pdf]

Supplementary File 1: Process-Evaluation Plan for GoActive Intervention

Component	Process Evaluation Question	Data sources	Tools/Procedures
Fidelity	*To what extent was the intervention implemented consistently and as planned?*	- Students<br>- Mentors<br>- Facilitators<br>- Teachers<br>- GoActive staff	Fidelity of implementation will be assessed utilizing an observation procedure*. This will include observing the encouragement and modelling of activities from mentors and leaders to students<br>- T2 student website use: Google analytics on points uploaded, and hence, prizes redeemed<br>- T2 mentor questionnaire<br>- T2 mentor log book<br>- T3 mentor focus groups<br>- T3 facilitator questionnaire*<br>- T3 facilitator focus groups/interviews*<br>- T2 facilitator log book<br>- T3 teacher questionnaire<br>- T3 teacher focus groups<br>- Field notes<br>- Interview notes<br>- Minutes of meetings<br>- Emails<br>- Logs (record keeping)<br>- Reflections
Dose delivered	*To what extent were the units within the intervention implemented?*	- Students<br>- Mentors<br>- Facilitators<br>- Teachers<br>- GoActive staff	- T2 student questionnaire<br>- T2 student focus groups<br>- T3 student individual interviews<br>- T2 mentor questionnaire<br>- T3 mentor focus groups<br>- T2 mentor log book<br>- T3 facilitator questionnaire*<br>- T3 facilitator groups/interviews*<br>- T2 facilitator log book<br>- T3 teacher questionnaire<br>- T3 teacher focus groups<br>- Documentation of staff activities<br>- Review of notes and other documents<br>- Classroom observations*
Dose received	*Did students enjoy the GoActive activities?*<br>*Were mentors, teachers and facilitators satisfied with the intervention?*<br>*Were the GoActive staff satisfied with the intervention?*	- Students<br>- Mentors	- T2 student website use: Google analytics on frequency and duration of website use, resources download and points upload statistics<br>- T2 student questionnaire<br>- T2 student focus groups<br>- T3 student individual interviews<br>- T2 mentor questionnaire<br>- T2 mentor log book

		- Facilitators<br><br>- Teachers<br><br>- Go Active staff	- T3 mentor focus groups<br>- T3 facilitator questionnaire*<br>- T3 facilitator focus groups*<br>- T2 facilitator log book<br>- T3 teacher questionnaire<br>- T3 teacher focus groups<br>- Field notes<br>- Interview notes<br>- Minutes of meetings<br>- Emails<br>- Logs<br>- Reflections<br>- Classroom observations*
Reach	*Was the intervention delivered to at least 75% of Year 9 students?*	- Students	- T2 student questionnaire
Recruitment	*What procedures were followed to recruit schools and participants (students, teachers, mentors and facilitators) to the GoActive intervention?*	- GoActive staff<br>- Mentors<br>- Students	- £200 sporting equipment voucher for schools<br>- Sports clothing for mentors<br>Maintenance for students:<br>- Awards<br>- Prizes<br>- Competition
Context	*What were barriers and facilitators to implementing the GoActive intervention?*	- Students<br><br>- Mentors<br><br>- Facilitators<br><br>- Teachers<br><br>- GoActive staff	- T2 student questionnaire<br>- T2 student focus groups<br>- T3 student individual interview<br>- T2 mentor questionnaire<br>- T3 mentor focus groups<br>- T2 mentor log book<br>- T3 facilitator questionnaire*<br>- T3 facilitator focus groups*<br>- T2 facilitator log book<br>- T2 teacher questionnaire<br>- T3 teacher focus groups<br>- Field notes<br>- Interview notes<br>- Minutes of meetings<br>- Emails<br>- Logs (record keeping)<br>- Reflections<br>- Classroom observations*

*Ethics approval pending for these elements.
