# Supplementary material for: A cluster randomised controlled trial to evaluate the effectiveness and cost-effectiveness of the GoActive intervention to increase physical activity among adolescents aged 13–14 years
Source: BMJ Open. 2017 Sep 27;7(9):e014419. doi: 10.1136/bmjopen-2016-014419 (PMC5623411; doi:10.1136/bmjopen-2016-014419)
Supplement: Author's manuscript [file bmjopen-2016-014419.draft_revisions.pdf]

BMJ Open

BMJ Open is committed to open peer review. As part of this commitment we make the peer review history of every article we publish publicly available.

When an article is published we post the peer reviewers' comments and the authors' responses online. We also post the versions of the paper that were used during peer review. These are the versions that the peer review comments apply to.

The versions of the paper that follow are the versions that were submitted during the peer review process. They are not the versions of record or the final published versions. They should not be cited or distributed as the published version of this manuscript.

BMJ Open is an open access journal and the full, final, typeset and author-corrected version of record of the manuscript is available on our site with no access controls, subscription charges or pay-per-view fees (http://bmjopen.bmj.com).

If you have any questions on BMJ Open's open peer review process please email editorial.bmjopen@bmj.com

BMJ Open

Protocol for a cluster randomised controlled trial to evaluate the effectiveness and cost-effectiveness of the GoActive intervention to increase physical activity among 13-14 year-old adolescents

Journal:	*BMJ Open*
Manuscript ID	bmjopen-2016-014419
Article Type:	Protocol
Date Submitted by the Author:	26-Sep-2016
Complete List of Authors:	Brown, Helen Elizabeth; University of Cambridge, MRC Epidemiology Unit and Centre for Diet and Activity Research<br>Whittle, Fiona; University of Cambridge, MRC Epidemiology Unit<br>Croxson, Caroline; University of Oxford, Nuffield Department of Primary Care Health Sciences<br>Sharp, Stephen; University of Cambridge, MRC Epidemiology Unit<br>Wilkinson, Paul; University of Cambridge, Department of Psychiatry<br>Wilson, Edward; University of Cambridge, Cambridge Centre for Health Services Research<br>van Sluijs, Esther; University of Cambridge, MRC Epidemiology Unit and Centre for Diet and Activity Research<br>Vignoles, Anna; University of Cambridge, Faculty of Education<br>Corder, Kirsten; University of Cambridge, MRC Epidemiology Unit and Centre for Diet and Activity Research
**Primary Subject Heading**:	Public health
Secondary Subject Heading:	Epidemiology, Sports and exercise medicine
Keywords:	physical activity, promotion, intervention, adolescent, protocol, school

SCHOLARONE™
Manuscripts

Protocol for a cluster randomised controlled trial to evaluate the effectiveness and cost-effectiveness of the GoActive intervention to increase physical activity among 13-14 year-old adolescents

Helen Elizabeth Brown[1*], Fiona Whittle[1], Caroline Croxson[2], Stephen J. Sharp[1], Paul Wilkinson[3], Edward CF Wilson[4], Esther MF van Sluijs[1], Anna Vignoles[5], Kirsten Corder[1]

[1]UKCRC Centre for Diet and Activity Research (CEDAR) *and* MRC Epidemiology Unit, University of Cambridge
[2]Nuffield Department of Primary Care Health Sciences, University of Oxford
[3]Department of Psychiatry, University of Cambridge
[4]Cambridge Centre for Health Services Research, University of Cambridge
[5]Faculty of Education, University of Cambridge
*Corresponding Author

Address for correspondence:
Dr Helen Elizabeth Brown
UK CRC Centre for Diet and Activity Research (CEDAR)
Department of MRC Epidemiology
University of Cambridge School of Clinical Medicine
Box 285 Institute of Metabolic Science
Cambridge Biomedical Campus
Cambridge
CB2 0QQ
Email: heb56@medschl.cam.ac.uk

Abstract

Background: Adolescent physical activity promotion is rarely effective, despite adolescence being critical for preventing physical activity decline. Low adolescent physical activity is likely to last into adulthood, increasing health risks. The GoActive intervention is evidence-based and was developed iteratively with adolescents and teachers. This intervention aims to increase physical activity through increased peer support, self-efficacy, group cohesion, self-esteem and friendship quality, and is implemented using a tiered-leadership system. We previously established feasibility in 1 school and conducted a pilot randomised controlled trial (RCT) in 3 schools.

Methods: We will conduct a school-based cluster RCT (CRCT) in 16 secondary schools targeting all Year 9 students (N=2400). In 8 schools, GoActive will run for 2 terms: weekly facilitation support from a council-funded health trainer will be offered in Term 1, with more distant support in Term 2. Tutor groups choose 2 weekly activities, encouraged by older adolescent mentors and weekly peer-leaders. Students gain points for trying new activities; points are entered into a between-class competition. Outcomes will be assessed at baseline, interim (week 6), post-intervention (week 14-16), and 10-month follow-up (main outcome). The primary outcome will be change from baseline in daily accelerometer-assessed moderate-to-vigorous

For peer review only - http://bmjopen.bmj.com/site/about/guidelines.xhtml

physical activity. Secondary outcomes include accelerometer-assessed activity intensities on weekdays/weekends; self-reported physical activity and psycho-social outcomes; cost-effectiveness and cost-utility analyses; mixed methods process evaluation integrating information from focus groups and participation logs/questionnaires.

Discussion: Given the lack of rigorously evaluated interventions, and the inclusion of objective measurement of physical activity, long-term follow-up, and testing of causal pathways, the results of a CRCT of the effectiveness and cost-effectiveness of GoActive are expected to add substantially to the limited evidence on adolescent physical activity promotion. Workshops will be held with key stakeholders including students, parents, teachers, school governors, and government representatives, to discuss plans for wider dissemination of the intervention.

Strengths and limitations of this study

- The GoActive evaluation study includes objective measurement of physical activity, long-term follow-up, and testing of causal pathways, to rigorously assess the effectiveness and cost-effectiveness of the GoActive programme.
- This manuscript reports in detail on the recruitment and randomisation procedures, gives an overview of the GoActive intervention, and describes the included measures and proposed analyses, in accordance with SPIRIT guidance.
- However, as the trial is currently underway, there are no results presented in this manuscript.

Trial registration number: ISRCTN31583496

Originally registered: 18/2/2014
Funding reference: NIHR-PHR 13/90/18
Intervention delivery costs will be borne by Essex and Cambridgeshire County Councils.
Sponsor: University of Cambridge, contact**:** Mrs Carolyn Read, University of Cambridge School of Clinical Medicine, Box 111 Cambridge Biomedical Campus, Cambridge, CB2 0SP, United Kingdom, cad50@medschl.cam.ac.uk
Contact for Public and Scientific Queries: As address for correspondence
Public title: To establish the effect of the GoActive programme to increase physical activity among 13-14 year-old (Year 9) adolescents.
Scientific title: A cluster randomised controlled trial to evaluate the effectiveness and cost-effectiveness of the GoActive programme to increase physical activity among 13-14 year-old adolescents.
Protocol version: 2.0

Keywords: physical activity, promotion, intervention, adolescent, health behaviour

For peer review only - http://bmjopen.bmj.com/site/about/guidelines.xhtml

Background

Physical activity is protective against obesity and related metabolic disorders in young people[1,2]. Meta-analytic data from 20,871 4-18 year olds suggest that every 10-minute increase in moderate-to-vigorous activity (MVPA) is associated with a smaller waist circumference (-0.52 cm) and lower fasting insulin (-0.028 pmol/L).[2] In adolescence however, physical activity declines 7% per year.[3] Low physical activity in adolescence is also likely to progress to adulthood inactivity,[4] increasing the risk of diabetes, cancer and mortality.[5,6] Adolescence is therefore a critical period to increase physical activity,[7] both due to the aforementioned decline and because pubertal, brain and social development during this time leads to new capacity for changing health behaviours,[8] increasing the likelihood of long term change.

The 2012 Chief Medical Officer's report states the importance of physical activity among young people[9] and a recent international expert panel concluded that developing effective and sustainable interventions to increase physical activity among young people is the most important priority in the physical activity research field.[10] Further, the recently published report from the All-Party Commission on Physical Activity calls specifically for the creation of active schools, including the provision of a more diverse and inclusive offer of physical activity.[11]

Reviews highlight the limited efficacy of existing adolescent physical activity promotion interventions.[12,13,14,15] Further, there is a lack of rigorous evaluation of those existing interventions; for example, in a meta-analysis of 30 studies with objective outcomes,[14] only two of the included studies focused on adolescents over the age of 13 years.[16,17] There is therefore an urgent need for the rigorous evaluation of potentially effective strategies to increase physical activity in adolescents.

Objectives

The primary aim of this study is to assess the 10-month effectiveness of the GoActive intervention to increase average daily objectively measured MVPA among 13-14 year-old adolescents. We will also assess the effect of GoActive immediately post-intervention, and on the following secondary outcomes: a) objectively assessed activity intensities during school time, weekday evenings and weekends; b) student-reported physical activity participation, self-efficacy, peer support, social networks, self-esteem, friendship quality (proposed mediators), and wellbeing, and school-level attendance and academic performance and c) body composition (body fat percentage and body mass index (BMI) z-score). We will investigate potential moderation of intervention effects by sex, socio-economic status, ethnicity, baseline activity level, and weight status, and potential mechanisms of effect by proposed mediators using a mixed-methods approach. Further, we will assess short term (within-trial) and potential long term cost-effectiveness of the GoActive intervention.

Intervention

The development of the "GoActive" (Get Others Active) intervention with supporting rationale has been described in detail previously.[18] Briefly, each Year 9 class (tutor group or home room class) chooses two activities each week from a selection provided. There are currently 20 activities available, utilising little or no equipment, and appealing to a wide variety of students (including Ultimate Frisbee, Zumba and Hula Hoop).

For peer review only - http://bmjopen.bmj.com/site/about/guidelines.xhtml

Materials available on the password-protected GoActive intervention website include activity instructions (Quick Cards) which offer an overview of each activity, a short explanation, suggestions for adaptations, and provide advice, safety tips and 'factoids', in addition to a short video introducing each activity. GoActive is implemented using a tiered-leadership system where mentors (older adolescents within the school) and peer-leaders (within each Year 9 class) encourage students to try these activities each week. The mentors remain paired with each class for the duration of the intervention, whereas the peer-leaders (two per class each week, one male and one female) change every week. In addition to the student leaders, a local authority-funded intervention facilitator will support the programme during the first term of delivery and will provide distant support thereafter.

Teachers are encouraged to use one tutor time weekly to do one of the chosen activities as a class, however, students gain points for trying these new activities at any time in or out of school. Points are gained every time they try an activity; there is no expectation of time spent doing the activity as points are rewarded for the taking part itself. Individual students keep track of their own points privately on the study website and their points are entered into the between-class competition. Class rankings are available on the website to encourage teacher support and students receive small rewards (such as a frisbee, or a water bottle) for reaching individual points thresholds.

Methods

Study design

We will conduct a school-based cluster randomised controlled trial (CRCT) of the GoActive intervention. The study will be conducted in government-funded, non-fee-paying (state), all-ability, co-educational secondary schools including Year 9 students in Cambridgeshire and Essex, UK. After baseline measurements (September - December 2016), schools will be randomly allocated to one of two conditions; (1) to deliver the GoActive intervention to the whole of Year 9, or (2) to a no-treatment control group. Participant data collection will occur at baseline, 6 weeks, 14-16 weeks and 10 months (primary outcome). The protocol will be conducted and reported in accordance with SPIRIT guidance (Standard Protocol Items: Recommendations for Interventional Trials).[19,20,21] The trial has been registered with the ISRCTN registry (trial registration number: ISRCTN31583496).

Ethical approval

Ethical approval for the conduct of the study will be sought from the Cambridge Psychology Research Ethics Committee, who previously provided ethical approval for the development, feasibility and pilot studies following similar procedures. [18,22]

Recruitment procedures

Schools

We will recruit 16 secondary schools with a mixture of socio-economic status, representative of UK variability. Head teachers, Year 9 leaders, and Physical Education (PE) leaders from all eligible schools will be sent an invitation letter and school information sheet via email. These documents will describe the study procedures (e.g. student recruitment and consent, measurements), and will include an electronic link to an information video describing GoActive. A follow-up phone call to each school will be made approximately one

For peer review only - http://bmjopen.bmj.com/site/about/guidelines.xhtml

week after the initial invitation, asking for a meeting with relevant staff to discuss the study and request consent to participate. Phone calls and repeat emails will continue until 16 schools (8 in Cambridgeshire, and 8 in Essex) have provided consent to participate. We will also create a waiting list to replace any schools who may withdraw from the study prior to randomisation. We will also use our existing networks and school contacts to facilitate school recruitment. Schools who do not agree to take part will be asked to select the most relevant reason for their refusal from a pre-determined list (e.g. lack of interest, lack of time).

Participants

All Year 9 students (13-14 year-olds) in participating schools will be eligible to participate in study measurements. As in feasibility and pilot work, we plan to include disabled participants and those with learning or movement difficulties, taking care to follow advice from schools.[22] This is appropriate due to the inclusive nature of the GoActive intervention, and will help to avoid stigmatisation of any groups within schools.[23] As such, no exclusion criteria will be applied.

All Year 9 students and their parents will receive a paper invitation pack, including a participant information sheet and an invitation to participate in study measurements. Parents will also be sent duplicate information via email (‘ParentMail’ or the appropriate equivalent system as agreed by the school). Parents will be asked to provide passive consent (active opt-out consent) for their child to take part in study measurements. We will give parents at least two weeks to respond (a final date for response will be included in all correspondence). After one week, parents will receive an additional copy to ensure further opportunity for opting out prior to study measurements. Parents will be given the option to phone or email the study team (in lieu of returning a form) to facilitate their ability to respond. Reminders will additionally be included in all relevant school media, including regular newsletters sent from the school. Written assent will be obtained from the students by research assistants trained in Good Clinical Practice prior to any baseline measurements taking place. Consent forms will be available on the study website www.goactive-uk.com after ethical approval for the trial has been obtained. Mentors and teachers will provide written consent or assent (for those older and younger than 16 years, respectively) to participate in process evaluation following the same procedures as study participants.

Parental opt-out responses ranged from 2 (<1%) to 18 (7%) in feasibility and pilot schools with 72-88% of eligible students assenting to participate.[22] Recruitment rates using this strategy are substantially higher than previous UK-based research in this age group using parental opt-in consent (23% of eligible participants).[7] Participants will be informed that they can discontinue all or any part of the study (either or both measurements and intervention) at any time at their or their parent/guardian’s request.

School randomisation

Schools will be stratified based on Pupil Premium (proxy for socio-economic status, below/above the county-specific median; for information: https://www.gov.uk/guidance/pupil-premium-information-for-schools-and-alternative-provision-settings), and county (i.e. Cambridgeshire or Essex). Randomisation lists for each stratum will be prepared by a statistician, using Stata (ref: StataCorp. 2015. Stata Statistical Software: Release 14. College Station, TX: StataCorp LP), after baseline measurements are completed to ensure schools and participants are unaware of their group allocation at baseline. Eight schools will be randomised

For peer review only - http://bmjopen.bmj.com/site/about/guidelines.xhtml

to deliver the GoActive intervention and eight to a no-treatment control condition. For measurements after randomisation, it will not be possible to blind participants to randomised allocation, as the intervention schools will have received the GoActive intervention.

Measurement staff will be blinded to intervention condition throughout the study, as they will be trained and work separately from those involved in intervention delivery. Process evaluation with measurement staff will examine the success of blinding.

Control condition

The control group will receive no-treatment or ‘usual care’, and no intervention will be implemented. If we were to offer the control group the intervention after follow-up measures, it would prevent us from potentially assessing longer-term impact of the programme. As such, this study has no wait-list control condition.

Data collection

Measurements will be conducted at four time points by trained researchers (Figure 1). The primary measure of intervention effectiveness will be change from baseline in accelerometer-measured average daily MVPA at 10-month follow-up. All primary and secondary outcomes will be assessed at T1, T3, and T4; T2 will focus on assessing the questionnaire-based measures (including mediators of change). To prevent artificially inflated school-level clustering (due to weather conditions or school events) and facilitate recruitment and retention, measurements at each school will be staggered over ≥2 weeks using a predetermined schedule.

Figure 1 near here

Accelerometry

The primary outcome will be accelerometer-assessed change in average daily MVPA between baseline and 10-month follow-up. Secondary accelerometry outcomes will be change from baseline in average minutes spent in sedentary and light activity, as well as overall physical activity (counts per minute) during school, weekdays after school and at weekends.

Participants will be asked to wear a wrist-worn Axivity AX3 monitor at T1, T3 and T4. Participants will be asked to wear the monitors on a strap on their non-dominant wrist, continuously for seven consecutive days, (including when in water and when asleep). Wrist-worn monitors have been validated for use among children and adolescents, in laboratory and free-living environments, and to assess physical activity, sedentary time and postural allocation.[24,25,26] There is evidence to support the increased acceptability and higher compliance rates of wrist-worn monitors compared to waist-worn monitors.[27,28,29,30,31,32,33] To further optimise accelerometer-wear compliance, we have developed a monitor wear and return protocol which is led by researchers (and not teachers), and includes regular reminders and an incentive. We have previously successfully applied this protocol in adolescent cohort studies to obtain high levels of valid accelerometry data (ROOTS: 825/930- 89%[7]; SPEEDY-3: 428/480 - 89%[34]).

Throughout data collection, we will continuously monitor response rates and take appropriate action (e.g. requesting teacher involvement) if it drops below 70% for the primary outcome. In cases where participants

For peer review only - http://bmjopen.bmj.com/site/about/guidelines.xhtml

do not return their accelerometer after frequent requests, they may not be issued a monitor at subsequent measurements, but will be allowed to continue their participation in the study and all other (secondary) measures. This is to prevent excessive monitor loss. We deem this appropriate as sample size calculations indicate that we will retain 95% power should retention drop to 55% (80/150 participants predicted to participate in each school based on pilot data).

Once returned, data (continuous waveform data) from the accelerometers will be downloaded. Non-wear time will be removed, using a criterion of consecutive runs of zero counts for a minimum duration of 60 minutes.[35,36] Remaining data will be included if accelerometer wear time ≥480 mins, on at least two days. Cut-points comparable to those used previously for ActiGraph accelerometers will be used to classify time spent sedentary (equivalent to ≤100 ActiGraph cpm), or in light (equivalent to 101 - 1999 ActiGraph cpm), moderate-vigorous (equivalent to ≥2000 ActiGraph cpm) or appropriate vector magnitude equivalents.[37,38,39] Monitor output will be reviewed prior to analysis to confirm that these decisions are appropriate for the population and monitor applied. Further, we will consult physical activity measurement experts to ensure we can be aware of relevant new methodology and apply where appropriate.

Anthropometry

Trained staff will measure height, weight and waist circumference following standardised operating procedures (e.g. wearing light clothing, removing shoes). Body fat percentage will be calculated from bio-electrical impedance, age- and sex-specific BMI z-score will be calculated from height and weight. Quality checking of researchers' anthropometry measurements will be conducted prior to baseline measurements and before 10-month follow-up.

Questionnaires

At each measurement session (i.e. T1, T2, T3, and T4), participants will complete a questionnaire concerning secondary outcomes, potential mediators or moderators, and items to monitor any adverse intervention effects. Physical activity type will be assessed using the 30-item Youth Physical Activity Questionnaire (YPAQ), which has previously been validated in 12-17 year olds.[40] Self-efficacy[41] and social support for physical activity[42] will be assessed using two scales (each with 3 items). Further items include friendship quality (8-item Cambridge Friendships Questionnaire),[43] well-being (14-item Edinburgh-Warwick Wellbeing Scale)[44] self-esteem (10- item Rosenberg Self Esteem Scale)[45], and an adapted social network modelling tool in which participants provided with a list of tutor group members and asked to select names of their friends)[46], and shyness and sociability (two 5-item measures from EAS temperament scale).[47] Questionnaires will be checked for completion before the end of the measurement sessions, and participants will be asked to complete any missing items. At T1, participants will respond to additional items providing demographic data (i.e. age, sex, ethnicity, language spoken at home, parent education, and family socio-economic status). School-level attendance and academic performance (from National Pupil Database) will be collected (publicly available data).

Process evaluation

Intervention process data will include mixed-methods assessment of student, mentor and teacher experiences, and perspectives on intervention delivery, feasibility, acceptance, and barriers/facilitators to

For peer review only - http://bmjopen.bmj.com/site/about/guidelines.xhtml

participation. Uptake, maintenance, and dose will be established using the points entries on the study website, download statistics for intervention materials and mentor-reported participation. Process questionnaires will be administered at T2 and T3 for (both intervention and control) participants, mentors, and form teachers. Control participants will also be asked to complete process questionnaires to determine possible contamination.[48] We will include a GoActive logbook for the intervention facilitator and mentors to assess frequency of intervention delivery. Given the flexible, spontaneous and informal nature of the intervention (mentors/leaders attend the same school and can therefore encourage/motivate Year 9 students at any time during the week), observation of intervention delivery is not deemed feasible. However, existing and emerging school practices which may affect students' physical activity behaviour will be documented and monitored in a structured manner using an adapted school environment questionnaire.[49]

A qualitative researcher will conduct semi-structured focus groups after the facilitated intervention phase (T2) with representatives from all relevant groups (Year 9 students, mentors, and teachers) in each intervention school. Each focus group (separate for students, mentors, and teachers) will comprise 3-8 individuals, with up to two facilitators. Students will be purposively sampled to ensure a mix of sex and ethnicity, and grouped by level of participation and physical activity. A topic guide will be developed and updated as new issues and themes emerge; participants will be encouraged to discuss additional issues. Issues arising will be incorporated into the next round of questionnaires and subsequent focus groups, so that additional mechanisms of change can be investigated. In addition to focus groups, interviews will be conducted with a purposive sample of inactive and shy participants (identified using questionnaire data) at intervention schools to provide a deeper understanding of their intervention experience, and barriers and facilitators to participation (we anticipate these individuals will be more comfortable participating in one-to-one interviews).

At T4, additional semi-structured focus groups and interviews with students will explore maintenance of physical activity behaviour change, including who did or did not maintain physical activity behaviour change and why, whether GoActive helped and why or how, and other factors that helped or hindered physical activity maintenance. T2 participants will be re-invited, supplemented by additional students if needed. This gives us a unique opportunity to explore physical activity maintenance across time in the context of a trial, and to better understand barriers and facilitators to physical activity maintenance.

Cost-effectiveness

We will conduct both a within-trial and decision-model based economic evaluation. The within-trial analysis will be from the cost perspective of the school/local authority. Cost data collected will include intervention-related facilitator time, travel, and expenses collected by schools/researchers. Outcomes will comprise change in MVPA and quality adjusted life years (QALYs) gained. These will be assessed using the CHU-9D[50] and converted to health state utilities using UK specific valuations.[51] Change in physical activity observed and costs to schools/local authorities will be input into a previously developed model to predict longer term costs (to the National Health Service (NHS)) and QALYS hence cost-effectiveness from a public sector perspective (defined as local authority and NHS).

Data collection forms and questionnaires for all measurements are available on request from the corresponding author.

For peer review only - http://bmjopen.bmj.com/site/about/guidelines.xhtml

Data management and monitoring

All data will be collected and managed in line with International Conference on Harmonisation Good Clinical Practice guidelines. Real time entry and retrospective data validation checks will be conducted. All paper based questionnaire data will be professionally double data entered and a sample verified for accuracy. Data will be stored securely at the MRC Epidemiology Unit, Cambridge, UK. The MRC Epidemiology Unit specialist teams will provide support for training, and quality assessment and control of measurements, and this support will ensure that collection, processing, protection and management of data is timely and of high quality. We will ensure that all provided data are treated as confidential and stored securely. Where this is electronic, data are held on secure computer systems with at minimum password access. All identifiable data will be held on a separate computer system with access limited to appropriate staff by group and password permissions. Personal data will be stored and accessed up to 20 years after study completion.

Due to the low risk nature of the trial, a formal data monitoring committee has not been appointed. However, the Trial Steering Committee (TSC) will receive regular reports from the investigators and will monitor trial progress and conduct. The TSC will consist of an independent chair, one independent expert, two lay representatives (including a representative from educational sector) and at least two investigators; the committee will be at least 75% independent. The study coordinator and a sponsor representative will be invited as observers. The TSC will meet approximately once per year, or more frequently if needed. The TSC is responsible for communicating any issues of concern to the Sponsor, specifically where the integrity of the study or data or patient safety could be comprised. The study coordinator will also monitor trial conduct and will report independently to the MRC Epidemiology Unit Clinical Research Manager. Potential harms will be monitored by the study team. These will be reviewed by the Study Coordinator, Principal Investigator, and Trial Steering Committee, and will include reported adverse events (e.g. injuries or psychological indicators such as well-being). While we do not expect harm as a result of the GoActive intervention or this trial, it is insured by the University of Cambridge who would provide compensation in case of harm.

The council-funded intervention facilitators will work closely with mentors and research staff to monitor protocol adherence. Poor adherence will be discussed with the research team and TSC, and strategies will be put in place where necessary. No activities are prohibited during the trial as students are expected to do their normal physical activities, including school PE.

Any protocol amendments will be proposed to the TSC and subsequently altered if necessary before submission to funder (NIHR) for approval. Protocol updates will then be uploaded to the NIHR website and trial registry if relevant.

Analyses

Sample size

We aim to detect a 5-minute difference in change in MVPA per day at 10-month follow-up, as observed in the pilot study.[22] A 5-minute increase is relevant at population level, as it would increase the proportion of adolescents meeting the guidelines of 60 minutes of MVPA per day from 43% to 50% (based on baseline pilot data), with significant impact on population health.[2] To estimate the required sample size, the following

For peer review only - http://bmjopen.bmj.com/site/about/guidelines.xhtml

parameters have been used: power=85%, significance level=5%, standard deviation=17.8 (observed in the GoActive pilot),[22] intraclass correlation coefficient=0.034 (observed in SPEEDY-3, N=57 schools),[39] correlation between baseline and follow-up MVPA=0.59 (observed in GoActive pilot, to account for adjustment for baseline MVPA),[22] and average cluster size=100. Based on these parameters, we estimate N=1310 participants will be required for the primary effectiveness analysis. To account for potential school dropout and an estimated loss-to-follow-up of 30-40%, we aim to recruit 16 schools with 150 participants (total N=2400; average recruitment per school in pilot=154).[22]

Quantitative analyses

The primary analysis of effectiveness, intermediate, and safety outcomes will use an Intention To Treat (ITT) population, which includes all participants in the group to which they were randomised, regardless of the intervention received. A secondary analysis of efficacy and intermediate outcomes will use a Per Protocol (PP) population. Inclusion in the PP population will be based on the degree of usage of the intervention website and/or submission of points, and will be defined once clean data are available (but before the start of any trial analyses), when the distributions of degree of website usage can be inspected.

Outcome analyses

The primary efficacy outcome, MVPA, will be compared between intervention and control groups using analysis of covariance (ANCOVA), with adjustment for baseline MVPA; robust standard errors will be calculated to allow for the non-independence of individuals within each school. Where baseline values of MVPA are missing, the missing indicator method will be used to enable these participants to be included in the analysis.[52] An estimate of the intervention effect, 95% confidence interval, and p-value will be calculated. A similar method will be used for the secondary efficacy outcomes. School-level data will also enable analysis of key differences between those participating in the evaluation, and the wider school population; for example, patterns of non-response by demographic variables will be explored. Subgroup analyses by pre-specified moderators (sex, socio-economic status, ethnicity, baseline activity level, weight status) will be performed for the primary outcome only. The interaction between randomised group and each moderator will be tested, and if the p-value is <0.05, the intervention effect (difference between intervention and control, and 95% confidence interval) will be estimated within each subgroup. The effect on potential mediating variables will initially be assessed as described above. We will subsequently conduct formal mediation analyses using the product of coefficient method[53] to assess the underlying causal pathways of the intervention.

Qualitative analyses

Focus groups and interviews will be audio recorded, transcribed verbatim and made anonymous. Data will be analysed using constant comparative analysis, facilitated by QSR NVivo. Coding will be inductive, incorporating emerging themes as well as topics presented a priori in the topic guide. Initial analyses will inform future data collection and analysis. Interim themes will be discussed by the research team to reach consensus.

For peer review only - http://bmjopen.bmj.com/site/about/guidelines.xhtml

Cost-effectiveness analyses

Cost-effectiveness analyses will follow standardised protocols.[54] The main economic outcome will be the incremental cost-effectiveness ratio, expressed as incremental costs per incremental change in physical activity (MVPA) and per QALY gained (based on CHU-9D) for the trial period (including follow-up). Data collected will include intervention time, travel, expenses, resource use, and study-specific costs. In addition, if GoActive increases physical activity, this should reduce adult chronic disease via changes in weight or BMI, and blood glucose. To establish whether GoActive could increase length and/or quality of life and at what cost, it is not practical to conduct lifetime follow-up, therefore we propose adjusting an existing decision-analytic model to estimate the impact of physical activity on disease risk, quality-adjusted life expectancy (QALY) and cost to the NHS. The modelled analysis will therefore be from a public sector perspective (schools/local authority and NHS).

Further analyses

Further research questions can be addressed using the cohort data, including (but not limited to) assessment of the predictors of activity maintenance, and the longitudinal association between physical activity/sedentary behaviour and a) academic performance; b) shyness and sociability; and c) friendship quality. All proposed analyses will be approved by the project group, and authorship of manuscripts will be informed by recommended guidelines.[55]

Wider dissemination

If successful, it would be appropriate to disseminate this programme to schools and councils across the UK (in addition to peer-reviewed publications). Towards the end of the project, a deliberative dialogue workshop will be held with key stakeholders including students, parents, teachers, school governors, and representatives from local/national government. This final workshop will focus on plans for dissemination of results, and will include discussion of the process of programme adaptation to a diverse range of secondary schools and further ways of ensuring long-term appeal for adolescents. We anticipate that dissemination could be facilitated through the study website, hosting intervention materials (including videos) and study information.

Discussion

Given the lack of rigorously evaluated interventions, the results of a CRCT of the effectiveness and cost-effectiveness of GoActive are expected to add substantially to the limited evidence on adolescent physical activity promotion. This study will include an objective, wrist-worn measure of physical activity, aligning with contemporary population surveillance studies[56,57,58] and ensuring greater protocol compliance for enhanced data retention and quality.[28,30,31] Achieving sustained health behaviour change is an established priority,[10] and so the inclusion of medium to long-term follow-up of participants will enable conclusions regarding the trajectories of change (in particular, whether any initial behaviour change is maintained). It will also form one of the largest cohorts in the field of adolescent physical activity promotion, providing many opportunities for secondary data analysis, in addition to testing causal pathways of effect and examining cost-effectiveness. Irrespective of study outcome, the evaluation of the GoActive intervention to increase physical activity in adolescents has the potential for significant academic impact.

For peer review only - http://bmjopen.bmj.com/site/about/guidelines.xhtml

Contributors: The PI, KC will have overall responsibility for project progress and direction; HB will be the day-to-day scientific lead for the project and FW will be the operational lead. EvS, PW and AV will advise on study procedures and evaluation from their respective disciplines; PW will additionally lead the design and evaluation of psychosocial outcomes. CC will lead the qualitative and mixed methods research. EW will lead the economic evaluation. Study sponsor and funders will have no role in the study design, collection, management, analysis, and interpretation of data, writing of the report; and the decision to submit the report for publication.

Funding

This project is funded by the National Institute for Health Research Public Health Research (13/90/18). Intervention delivery costs will be borne by Essex and Cambridgeshire County Councils.

For peer review only

Figure 1: Measurement sessions included in the GoActive evaluation

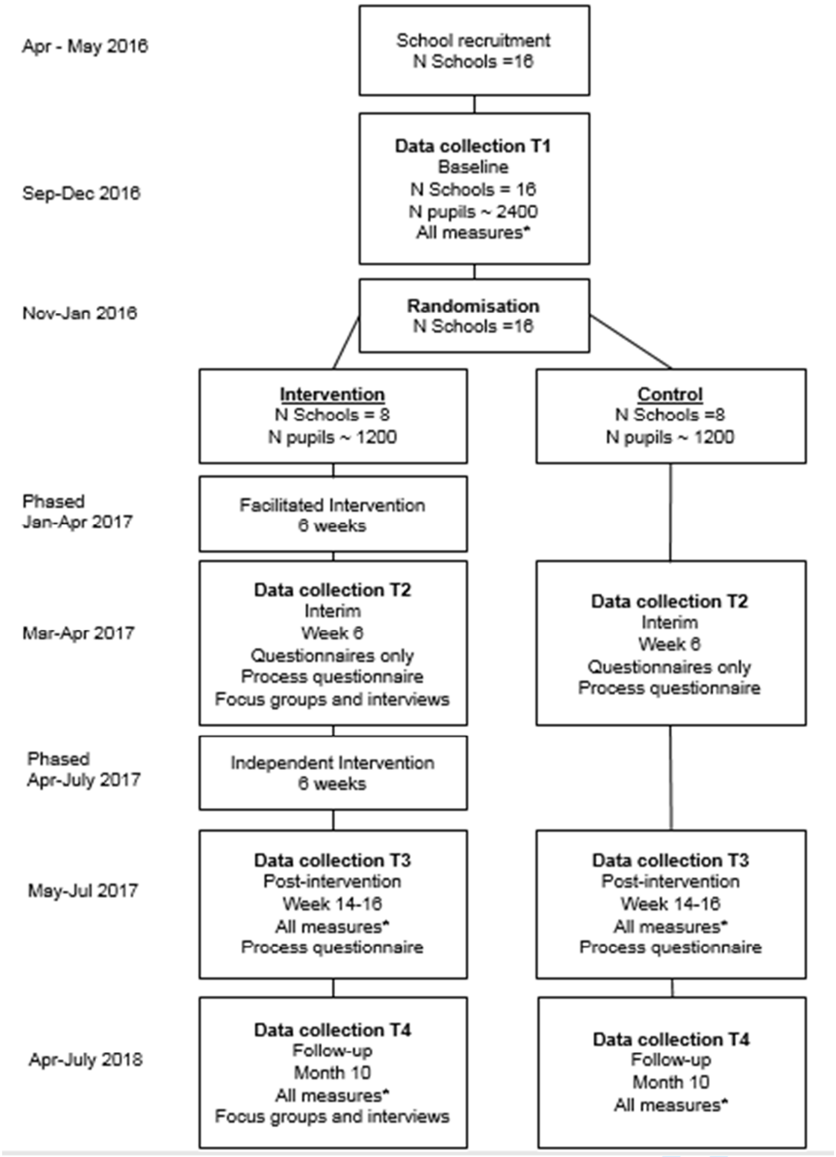

*All measures includes accelerometry, anthropometry and outcomes questionnaire (student-reported physical activity participation, self-efficacy, peer support, group cohesion, self-esteem, friendship quality, and mood).

Data sharing statement

After publication of trial analyses, and pending review of data access proposals by the investigators, data will be available on request from the corresponding author. The Principal Investigator, Co-Investigators, Statistician and Chair of the TSC will have access to the final trial dataset prior to conduct of the trial analyses. Statistical code for trial analysis will be available on request from the corresponding author.

For peer review only - http://bmjopen.bmj.com/site/about/guidelines.xhtml

There are no competing interests reported.

References

1 Janssen I, LeBlanc AG. Systematic review of the health benefits of physical activity and fitness in school-aged children and youth. Int. J. Behav. Nutr. Phys. Act. 2010;**7**:40.

2 Ekelund U, Luan J, Sherar LB, *et al.* Moderate to Vigorous Physical Activity and Sedentary Time and Cardiometabolic Risk Factors in Children and Adolescents. *JAMA J Am Med Assoc* 2012;**307**:704–12.

3 Dumith SC, Gigante DP, Domingues MR, *et al.* Physical activity change during adolescence: a systematic review and a pooled analysis. *Int J Epidemiol* 2011;**40**:685–98.

4 Raustorp A, Ekroth Y. Tracking of pedometer-determined physical activity: a 10-year follow-up study from adolescence to adulthood in Sweden. *J Phys Act Heal* 2013;**10**:1186–92.

5 Gill JMR, Cooper AR. Physical activity and prevention of type 2 diabetes mellitus. *Sports Med* 2008;**38**:807–24.

6 Khaw K-T, Wareham N, Bingham S, *et al.* Combined impact of health behaviours and mortality in men and women: the EPIC-Norfolk prospective population study. *PLoS Med* 2008;**5**:e12.

7 Corder K, Sharp SJ, Atkin AJ, *et al.* Change in objectively measured physical activity during the transition to adolescence. *Br J Sports Med* 2015;**49**:730–6.

8 Viner RM, Ozer EM, Denny S, *et al.* Adolescence and the social determinants of health. *Lancet* 2012;**379**:1641–52.

9 Department of Health. Annual Report of the Chief Medical Officer 2012: 'Our Children Deserve Better: Prevention Pays'. 2013. https://www.gov.uk/government/uploads/system/uploads/attachment_data/file/255237/2901304_CMO_complete_low_res_accessible.pdf

10 Gillis L, Tomkinson G, Olds T, *et al.* Research priorities for child and adolescent physical activity and sedentary behaviours: an international perspective using a twin-panel Delphi procedure. *Int J Behav Nutr Phys Act* 2013;**10**:112.

11 Report: Tackling Physical Inactivity— A Coordinated Approach | All-Party Commission on Physical Activity on WordPress.com. https://activitycommission.com/report-tackling-physical-inactivity-a-coordinated-approach/ (accessed 21 Jul2016).

12 van Sluijs EMF, McMinn AM, Griffin SJ. Effectiveness of interventions to promote physical activity in children and adolescents: systematic review of controlled trials. *BMJ* 2007;**335**:703.

13 Kriemler S, Meyer U, Martin E, *et al.* Effect of school-based interventions on physical activity and fitness in children and adolescents: a review of reviews and systematic update. *Br J Sports Med* 2011;**45**:923–30.

14 Metcalf B, Henley W, Wilkin T. Effectiveness of intervention on physical activity of children: systematic review and meta-analysis of controlled trials with objectively measured outcomes (EarlyBird 54). *BMJ* 2012;**345**:e5888.

15 Dobbins M, DeCorby K, Robeson P, *et al.* School-based physical activity programs for promoting physical activity and fitness in children and adolescents aged 6-18. *Cochrane Database Syst Rev* Published Online First: 2009.http://www.scopus.com/inward/record.url?eid=2-s2.0-70249099867&partnerID=40&md5=1ad81652911cae49189a325a9acf1c25

16 Jago R, Fox KR, Page AS, *et al.* Parent and child physical activity and sedentary time: Do active parents foster active children? *BMC Public Health* 2010;**10**.http://www.scopus.com/inward/record.url?eid=2-s2.0-77950788581&partnerID=40&md5=2fdfcc761212f6d6cea046fc5b556b89

17 Haerens L, Deforche B, Maes L, *et al.* Evaluation of a 2-year physical activity and healthy eating intervention in middle school children. *Health Educ Res* 2006;**21**:911–21.

18 Corder K, Schiff A, Kesten JM, *et al.* Development of a universal approach to increase physical activity among adolescents: the GoActive intervention. *BMJ Open* 2015;**5**:e008610.

19 Chan AW, Tetzlaff JM, Altman DG, *et al.* SPIRIT 2013 statement: Defining standard protocol items for clinical trials. Ann. Intern. Med. 2013;**158**:200–7.

20 Chan AW, Tetzlaff JM, Altman DG, *et al.* SPIRIT 2013: New guidance for content of clinical trial protocols. Lancet. 2013;**381**:91–2.

For peer review only - http://bmjopen.bmj.com/site/about/guidelines.xhtml

21 Chan A-W, Tetzlaff JM, Gøtzsche PC, *et al.* SPIRIT 2013 explanation and elaboration: guidance for protocols of clinical trials. *BMJ* 2013;**346**:e7586.

22 Corder, Kirsten; Brown, Helen Elizabeth; Schiff, Anne;, van Sluijs EM. Feasibility study and pilot cluster randomised controlled trial of the GoActive Intervention aiming to promote physical activity among adolescents: outcomes and lessons learnt. *BMJ Open*

23 Shochet IM, Dadds MR, Holland D, *et al.* The efficacy of a universal school-based program to prevent adolescent depression. *J Clin Child Psychol* 2001;**30**:303–15.

24 Rowlands A V, Olds TS, Hillsdon M, *et al.* Assessing sedentary behavior with the GENEActiv: introducing the sedentary sphere. *Med Sci Sports Exerc* 2014;**46**:1235–47.

25 Phillips LRS, Parfitt G, Rowlands A V. Calibration of the GENEA accelerometer for assessment of physical activity intensity in children. *J Sci Med Sport* 2013;**16**:124–8.

26 Schaefer CA, Nigg CR, Hill JO, *et al.* Establishing and evaluating wrist cutpoints for the GENEActiv accelerometer in youth. *Med Sci Sports Exerc* 2014;**46**:826–33.

27 Rowlands A V., Yates T, Olds TS, *et al.* Sedentary sphere: Wrist-worn accelerometer-brand independent posture classification. *Med Sci Sports Exerc* 2016;**48**:748–54.

28 Rowlands A V., Rennie K, Kozarski R, *et al.* Children's physical activity assessed with wrist- and hip-worn accelerometers. *Med Sci Sports Exerc* 2014;**46**:2308–16.

29 Rowlands A V., Fraysse F, Catt M, *et al.* Comparability of measured acceleration from accelerometry-based activity monitors. *Med Sci Sports Exerc* 2015;**47**:201–10.

30 Rosenberger ME, Buman MP, Haskell WL, *et al.* 24 Hours of Sleep, Sedentary Behavior, and Physical Activity with Nine Wearable Devices. *Med Sci Sports Exerc* 2015;:457–65.

31 Hildebrand M, Van Hees VT, Hansen BH, *et al.* Age group comparability of raw accelerometer output from wrist-and hip-worn monitors. *Med Sci Sports Exerc* 2014;**46**:1816–24.

32 Fairclough SJ, Noonan R, Rowlands A V., *et al.* Wear compliance and activity in children wearing wrist- and hip-mounted accelerometers. *Med Sci Sports Exerc* 2016;**48**:245–53.

33 Edwardson CLL, Rowlands A V., Bunnewell S, *et al.* Accuracy of Posture Allocation Algorithms for Thigh- and Waist-Worn Accelerometers. Med. Sci. Sports Exerc. 2016.

34 Corder K, Atkin AJ, Ekelund U, *et al.* What do adolescents want in order to become more active? *BMC Public Health* 2013;**13**:718.

35 Mattocks C, Ness A, Leary S, *et al.* Use of accelerometers in a large field-based study of children: protocols, design issues, and effects on precision. *J Phys Act Health* 2008;**5 Suppl 1**:S98–111.

36 Riddoch CJ, Bo Andersen L, Wedderkopp N, *et al.* Physical activity levels and patterns of 9- and 15-yr-old European children. *Med Sci Sport Exerc* 2004;**36**:86–92.

37 Trost SG, Loprinzi PD, Moore R, *et al.* Comparison of accelerometer cut points for predicting activity intensity in youth. *Med Sci Sports Exerc* 2011;**43**:1360–8.

38 Van Sluijs EMF, Skidmore PML, Mwanza K, *et al.* Physical activity and dietary behaviour in a population-based sample of British 10-year old children: the SPEEDY study (Sport, Physical activity and Eating behaviour: environmental Determinants in Young people). *BMC Public Health* 2008;**8**:388.

39 Corder K, van Sluijs EMF, Ekelund U, *et al.* Changes in children's physical activity over 12 months: longitudinal results from the SPEEDY study. *Pediatrics* 2010;**126**:e926–35.

40 Corder K, van Sluijs EMF, Wright A, *et al.* Is it possible to assess free-living physical activity and energy expenditure in young people by self-report? *Am J Clin Nutr* 2009;**89**:862–70.

41 Saunders RP, Pate RR, Felton G, *et al.* Development of Questionnaires to Measure Psychosocial Influences on Children's Physical Activity. *Prev Med (Baltim)* 1997;**26**:241–7.

42 Ommundsen Y, Page A, Ku PW, *et al.* Cross-cultural, age and gender validation of a computerised questionnaire measuring personal, social and environmental associations with children's physical activity: The European Youth Heart Study. *Int J Behav Nutr Phys Act* 2008;**5**:29.http://www.ncbi.nlm.nih.gov/entrez/query.fcgi?cmd=Retrieve&db=PubMed&dopt=Citation&list_uids=18489736

43 Goodyer IM, Herbert J, Tamplin a, *et al.* Short-term outcome of major depression: II. Life events, family dysfunction, and friendship difficulties as predictors of persistent disorder. *J Am Acad Child Adolesc Psychiatry* 1997;**36**:474–80.

44 Clarke A, Friede T, Putz R, *et al.* Warwick-Edinburgh Mental Well-being Scale (WEMWBS): validated

For peer review only

for teenage school students in England and Scotland. A mixed methods assessment. *BMC Public Health* 2011;**11**:487.

45 Rosenberg M. *Society and the adolescent self-image*. NJ: Princeton: : Princeton University Press 1965.

46 Eys M, Loughead T, Bray SR, *et al.* Development of a cohesion questionnaire for youth: the Youth Sport Environment Questionnaire. *J Sport Exerc Psychol* 2009;**31**:390–408.

47 Buss AH, Plomin R. *Temperament: Early developing personality traits*. 1984.

48 Salmon J, Jorna M, Hume C, *et al.* A translational research intervention to reduce screen behaviours and promote physical activity among children: Switch-2-Activity. *Health Promot Int* 2011;**26**:311–21.

49 Wyatt KM, Lloyd JJ, Abraham C, *et al.* The Healthy Lifestyles Programme (HeLP), a novel school-based intervention to prevent obesity in school children: study protocol for a randomised controlled trial. *Trials* 2013;**14**:95.

50 Stevens K. Assessing the performance of a new generic measure of health related quality of life for children and refining it for use in health state valuation. *Appl Heal Econ Heal Policy* 2011;**9**:157–69.

51 Stevens K. Valuation of the child health utility 9D index. *Pharmacoeconomics* 2012;**30**:729–47.

52 White IR, Thompson SG. Adjusting for partially missing baseline measurements in randomized trials. *Stat Med* 2005;**24**:993–1007.

53 MacKinnon DP, Fairchild AJ, Fritz MS. Mediation analysis. *Annu Rev Psychol* 2007;**58**:593–614.

54 Drummond MF, Stoddard GL, Torrance GW. Methods for the economic evaluation of health care programmes. Methods Econ. Eval. Heal. Care Program. 1988;:39–71.

55 ICMJE. Recommendations: Defining the Role of Authors and Contributors. http://www.icmje.org/recommendations/browse/roles-and-responsibilities/defining-the-role-of-authors-and-contributors.html (accessed 9 Aug2016).

56 Troiano R MCJ. Objective Measures of Physical Activity, Sleep, and Strength in U.S. National Health and Nutrition Examination Survey (NHANES) 2011-2014. *8th Intern Conf Diet Act Methods*

57 Mannini A, Intille SS, Rosenberger M, *et al.* Activity recognition using a single accelerometer placed at the wrist or ankle. *Med Sci Sports Exerc* 2013;**45**:2193–203.

58 Sudlow C, Gallacher J, Allen N, *et al.* UK biobank: an open access resource for identifying the causes of a wide range of complex diseases of middle and old age. *PLoS Med* 2015;**12**:e1001779.

For peer review only

For peer review only - http://bmjopen.bmj.com/site/about/guidelines.xhtml

BMJ Open

Protocol for a cluster randomised controlled trial to evaluate the effectiveness and cost-effectiveness of the GoActive intervention to increase physical activity among 13-14 year-old adolescents

Journal:	*BMJ Open*
Manuscript ID	bmjopen-2016-014419.R1
Article Type:	Protocol
Date Submitted by the Author:	15-Dec-2016
Complete List of Authors:	Brown, Helen Elizabeth; University of Cambridge, MRC Epidemiology Unit and Centre for Diet and Activity Research<br>Whittle, Fiona; University of Cambridge, MRC Epidemiology Unit<br>Croxson, Caroline; University of Oxford, Nuffield Department of Primary Care Health Sciences<br>Sharp, Stephen; University of Cambridge, MRC Epidemiology Unit<br>Wilkinson, Paul; University of Cambridge, Department of Psychiatry<br>Wilson, Edward; University of Cambridge, Cambridge Centre for Health Services Research<br>van Sluijs, Esther; University of Cambridge, MRC Epidemiology Unit and Centre for Diet and Activity Research<br>Vignoles, Anna; University of Cambridge, Faculty of Education<br>Corder, Kirsten; University of Cambridge, MRC Epidemiology Unit and Centre for Diet and Activity Research
**Primary Subject Heading**:	Public health
Secondary Subject Heading:	Epidemiology, Sports and exercise medicine
Keywords:	physical activity, promotion, intervention, adolescent, protocol, school

SCHOLARONE™
Manuscripts

Protocol for a cluster randomised controlled trial to evaluate the effectiveness and cost-effectiveness of the GoActive intervention to increase physical activity among 13-14 year-old adolescents

Helen Elizabeth Brown[1*], Fiona Whittle[1], Caroline Croxson[2], Stephen J. Sharp[1], Paul Wilkinson[3], Edward CF Wilson[4], Esther MF van Sluijs[1], Anna Vignoles[5], Kirsten Corder[1]

[1]UKCRC Centre for Diet and Activity Research (CEDAR) *and* MRC Epidemiology Unit, University of Cambridge
[2]Nuffield Department of Primary Care Health Sciences, University of Oxford
[3]Department of Psychiatry, University of Cambridge
[4]Cambridge Centre for Health Services Research, University of Cambridge
[5]Faculty of Education, University of Cambridge
*Corresponding Author

Address for correspondence:
Dr Helen Elizabeth Brown
UK CRC Centre for Diet and Activity Research (CEDAR)
Department of MRC Epidemiology
University of Cambridge School of Clinical Medicine
Box 285 Institute of Metabolic Science
Cambridge Biomedical Campus
Cambridge
CB2 0QQ
Email: heb56@medschl.cam.ac.uk

Abstract

Background: Adolescent physical activity promotion is rarely effective, despite adolescence being critical for preventing physical activity decline. Low adolescent physical activity is likely to last into adulthood, increasing health risks. The GoActive intervention is evidence-based and was developed iteratively with adolescents and teachers. This intervention aims to increase physical activity through increased peer support, self-efficacy, group cohesion, self-esteem and friendship quality, and is implemented using a tiered-leadership system. We previously established feasibility in 1 school and conducted a pilot randomised controlled trial (RCT) in 3 schools.

Methods: We will conduct a school-based cluster RCT (CRCT) in 16 secondary schools targeting all Year 9 students (N=2400). In 8 schools, GoActive will run for 2 terms: weekly facilitation support from a council-funded health trainer will be offered in Term 1, with more distant support in Term 2. Tutor groups choose 2 weekly activities, encouraged by older adolescent mentors and weekly peer-leaders. Students gain points for trying new activities; points are entered into a between-class competition. Outcomes will be assessed at baseline, interim (week 6), post-intervention (week 14-16), and 10-month follow-up (main outcome). The primary outcome will be change from baseline in daily accelerometer-assessed moderate-to-vigorous

For peer review only - http://bmjopen.bmj.com/site/about/guidelines.xhtml

physical activity. Secondary outcomes include accelerometer-assessed activity intensities on weekdays/weekends; self-reported physical activity and psycho-social outcomes; cost-effectiveness and cost-utility analyses; mixed methods process evaluation integrating information from focus groups and participation logs/questionnaires.

Discussion: Given the lack of rigorously evaluated interventions, and the inclusion of objective measurement of physical activity, long-term follow-up, and testing of causal pathways, the results of a CRCT of the effectiveness and cost-effectiveness of GoActive are expected to add substantially to the limited evidence on adolescent physical activity promotion. Workshops will be held with key stakeholders including students, parents, teachers, school governors, and government representatives, to discuss plans for wider dissemination of the intervention.

Strengths and limitations of this study

- The GoActive evaluation study uses a cluster-randomised controlled trial design and includes objective measurement of physical activity, long-term follow-up, and testing of causal pathways, to rigorously assess the effectiveness and cost-effectiveness of the GoActive programme.
- We will recruit 16 secondary schools from both Essex and Cambridgeshire. Despite our purposive sampling of schools with varied socio-economic status, it is likely that participants may not be entirely representative of the wider UK population (particularly with regards to ethnicity).
- This manuscript reports in detail on the recruitment and randomisation procedures, gives an overview of the GoActive intervention, and describes the included measures and proposed analyses, in accordance with SPIRIT guidance. As the trial is currently underway, there are no results presented in this manuscript.

Trial registration number: ISRCTN31583496

Originally registered: 18/2/2014
Funding reference: NIHR-PHR 13/90/18
Intervention delivery costs will be borne by Essex and Cambridgeshire County Councils.
Sponsor: University of Cambridge, contact**:** Mrs Carolyn Read, University of Cambridge School of Clinical Medicine, Box 111 Cambridge Biomedical Campus, Cambridge, CB2 0SP, United Kingdom, cad50@medschl.cam.ac.uk
Contact for Public and Scientific Queries: As address for correspondence
Public title: To establish the effect of the GoActive programme to increase physical activity among 13-14 year-old (Year 9) adolescents.
Scientific title: A cluster randomised controlled trial to evaluate the effectiveness and cost-effectiveness of the GoActive programme to increase physical activity among 13-14 year-old adolescents.
Protocol version: 2.0

Keywords: physical activity, promotion, intervention, adolescent, health behaviour

For peer review only
For peer review only - http://bmjopen.bmj.com/site/about/guidelines.xhtml

Background

Physical activity is protective against obesity and related metabolic disorders in young people[1,2]. Meta-analytic data from 20,871 4-18 year olds suggest that every 10-minute increase in moderate-to-vigorous activity (MVPA) is associated with a smaller waist circumference (-0.52 cm) and lower fasting insulin (-0.028 pmol/L).[2] In adolescence however, physical activity declines 7% per year.[3] Low physical activity in adolescence is also likely to progress to adulthood inactivity,[4] increasing the risk of diabetes, cancer and mortality.[5,6] Adolescence is therefore a critical period to increase physical activity,[7] both due to the aforementioned decline and because pubertal, brain and social development during this time leads to new capacity for changing health behaviours,[8] increasing the likelihood of long term change.

The 2012 Chief Medical Officer's report states the importance of physical activity among young people[9] and a recent international expert panel concluded that developing effective and sustainable interventions to increase physical activity among young people is the most important priority in the physical activity research field.[10] Further, the recently published report from the All-Party Commission on Physical Activity calls specifically for the creation of active schools, including the provision of a more diverse and inclusive offer of physical activity.[11]

Reviews highlight the limited efficacy of existing adolescent physical activity promotion interventions.[12–15] We have previously identified several possible reasons for this lack of effectiveness[16]; for example, many interventions only target subgroups (such as girls[17] or low socio-economic groups[18]) despite activity declining among all groups.[16] We aim to recruit the whole school year group for evaluation, and to target all groups in the GoActive intervention, which to our knowledge has rarely been done in physical activity promotion interventions. In addition, the decline in activity mainly occurs out of school;[16] however, many interventions only target specific school-based times; for example, school time[13,19] or Physical Education lessons.[20] whereas GoActive encourages participants to do more activity both in and out of school. Further, very few adolescent physical activity interventions, especially among older adolescents, have been evaluated using objective measurement of physical activity,[14] and including long-term follow-up, process evaluation, or an assessment of cost-effectiveness.[21] This therefore highlights an urgent need for more rigorous evaluation of potentially effective strategies to increase physical activity in adolescents.

Objectives

The primary aim of this study is to assess the 10-month effectiveness of the GoActive intervention to increase average daily objectively measured MVPA among 13-14 year-old adolescents. We will also assess the effect of GoActive immediately post-intervention, and on the following secondary outcomes: a) objectively assessed activity intensities during school time, weekday evenings and weekends; b) student-reported physical activity participation, self-efficacy, peer support, social networks, self-esteem, friendship quality (proposed mediators), and wellbeing, and school-level attendance and academic performance and c) body composition (body fat percentage and body mass index (BMI) z-score). We will investigate potential moderation of intervention effects by sex, socio-economic status, ethnicity, baseline activity level, and weight status, and potential mechanisms of effect by proposed mediators using a mixed-methods approach. Further, we will assess short term (within-trial) and potential long term cost-effectiveness of the GoActive

For peer review only - http://bmjopen.bmj.com/site/about/guidelines.xhtml

intervention, and will conduct a comprehensive process evaluation including questionnaires, focus groups (with participants, mentors, and teachers), individual interviews, data from intervention logs, and website analytics.

Intervention

The development of the "GoActive" (Get Others Active) intervention with supporting rationale has been described in detail previously.[22] Briefly, each Year 9 class (tutor group or home room class) chooses two activities each week from a selection provided. There are currently 20 activities available, utilising little or no equipment, and appealing to a wide variety of students (including Ultimate Frisbee, Zumba and Hula Hoop). Materials available on the password-protected GoActive intervention website include activity instructions (Quick Cards) which offer an overview of each activity, a short explanation, suggestions for adaptations, and provide advice, safety tips and 'factoids', in addition to a short video introducing each activity. GoActive is implemented using a tiered-leadership system where mentors (older adolescents within the school) and peer-leaders (within each Year 9 class) encourage students to try these activities each week. The mentors remain paired with each class for the duration of the intervention, whereas the peer-leaders (two per class each week, one male and one female) change every week. In addition to the student leaders, a local authority-funded intervention facilitator will support the programme during the first term of delivery and will provide distant support thereafter.

Teachers are encouraged to use one tutor time weekly to do one of the chosen activities as a class, however, students gain points for trying these new activities at any time in or out of school. Points are gained every time they try an activity; there is no expectation of time spent doing the activity as points are rewarded for the taking part itself. Individual students keep track of their own points privately on the study website and their points are entered into the between-class competition. Class rankings are available on the website to encourage teacher support and students receive small rewards (such as a Frisbee, a t-shirt, or a drawstring sports bag) for reaching individual points thresholds.

Methods

Study design

We will conduct a school-based cluster randomised controlled trial (CRCT) of the GoActive intervention. The study will be conducted in government-funded, non-fee-paying (state), all-ability, co-educational secondary schools including Year 9 students in Cambridgeshire and Essex, UK. After baseline measurements (September - December 2016), schools will be randomly allocated to one of two conditions; (1) to deliver the GoActive intervention to the whole of Year 9, or (2) to a no-treatment control group. Participant data collection will occur at baseline, 6 weeks, 14-16 weeks and 10 months (primary outcome). The protocol will be conducted and reported in accordance with SPIRIT guidance (Standard Protocol Items: Recommendations for Interventional Trials).[23–25] The trial has been registered with the ISRCTN registry (trial registration number: ISRCTN31583496).

Ethical approval

For peer review only - http://bmjopen.bmj.com/site/about/guidelines.xhtml

Ethical approval for the conduct of the study was gained from the Cambridge Psychology Research Ethics Committee, who previously provided ethical approval for the development, feasibility and pilot studies following similar procedures. [22,26]

Recruitment procedures

Schools

We will recruit 16 secondary schools with a mixture of socio-economic status, representative of UK variability. Head teachers, Year 9 leaders, and Physical Education (PE) leaders from all eligible schools will be sent an invitation letter and school information sheet via email. These documents will describe the study procedures (e.g. student recruitment and consent, measurements), and will include an electronic link to an information video describing GoActive. A follow-up phone call to each school will be made approximately one week after the initial invitation, asking for a meeting with relevant staff to discuss the study and request consent to participate. Phone calls and repeat emails will continue until 16 schools (8 in Cambridgeshire, and 8 in Essex) have provided consent to participate. We will also create a waiting list to replace any schools who may withdraw from the study prior to randomisation. We will also use our existing networks and school contacts to facilitate school recruitment. Schools who do not agree to take part will be asked to select the most relevant reason for their refusal from a pre-determined list (e.g. lack of interest, lack of time).

Participants

All Year 9 students (13-14 year-olds) in participating schools will be eligible to participate in study measurements. As in feasibility and pilot work, we plan to include disabled participants and those with learning or movement difficulties, taking care to follow advice from schools.[26] This is appropriate due to the inclusive nature of the GoActive intervention, and will help to avoid stigmatisation of any groups within schools.[27] As such, no exclusion criteria will be applied.

All Year 9 students and their parents will receive a paper invitation pack, including a participant information sheet and an invitation to participate in study measurements. These information packs will be distributed to students during an introductory assembly conducted by a member of the GoActive team; students will be asked to take the packs home to their parents. Parents will also be sent duplicate information via email ('ParentMail' or the appropriate equivalent system as agreed by the school). Parents will be asked to provide passive consent (active opt-out consent) for their child to take part in study measurements. We will give parents at least two weeks to respond (a final date for response will be included in all correspondence). After one week, parents will receive an additional copy to ensure further opportunity for opting out prior to study measurements. Parents will be given the option to phone or email the study team (in lieu of returning a form) to facilitate their ability to respond. Reminders will additionally be included in all relevant school media, including regular newsletters sent from the school. Written assent will be obtained from the students by research assistants trained in Good Clinical Practice prior to any baseline measurements taking place. Consent forms will be available on the study website www.goactive-uk.com after ethical approval for the trial has been obtained. Mentors and teachers will provide written consent or assent (for those older and younger than 16 years, respectively) to participate in process evaluation following the same procedures as study participants.

For peer review only - http://bmjopen.bmj.com/site/about/guidelines.xhtml

Parental opt-out responses ranged from 2 (<1%) to 18 (7%) in feasibility and pilot schools with 72-88% of eligible students assenting to participate.[26] Recruitment rates using this strategy are substantially higher than previous UK-based research in this age group using parental opt-in consent (23% of eligible participants).[7] Participants will be informed that they can discontinue all or any part of the study (either or both measurements and intervention) at any time at their or their parent/guardian's request.

School randomisation

Schools will be stratified based on Pupil Premium (proxy for socio-economic status, below/above the county-specific median; for information: https://www.gov.uk/guidance/pupil-premium-information-for-schools-and-alternative-provision-settings), and county (i.e. Cambridgeshire or Essex). Randomisation lists for each stratum will be prepared by a statistician, using Stata (ref: StataCorp. 2015. Stata Statistical Software: Release 14. College Station, TX: StataCorp LP), after baseline measurements are completed to ensure schools and participants are unaware of their group allocation at baseline. Eight schools will be randomised to deliver the GoActive intervention and eight to a no-treatment control condition. For measurements after randomisation, it will not be possible to blind participants to randomised allocation, as the intervention schools will have received the GoActive intervention.

Measurement staff will be blinded to intervention condition throughout the study, as they will be trained and work separately from those involved in intervention delivery. Process evaluation with measurement staff will examine the success of blinding.

Control condition

The control group will receive no-treatment or 'usual care', and no intervention will be implemented. If we were to offer the control group the intervention after follow-up measures, it would prevent us from potentially assessing longer-term impact of the programme. As such, this study has no wait-list control condition.

Data collection

Measurements will be conducted at four time points by trained researchers (Figure 1). The primary measure of intervention effectiveness will be change from baseline in accelerometer-measured average daily MVPA at 10-month follow-up. All primary and secondary outcomes will be assessed at T1, T3, and T4; T2 will focus on assessing the questionnaire-based measures (including mediators of change). To prevent artificially inflated school-level clustering (due to weather conditions or school events) and facilitate recruitment and retention, measurements at each school will be staggered over ≥2 weeks using a predetermined schedule.

Figure 1 near here

Accelerometry

The primary outcome will be accelerometer-assessed change in average daily MVPA between baseline and 10-month follow-up. Secondary accelerometry outcomes will be change from baseline in average minutes spent in sedentary and light activity, as well as overall physical activity (counts per minute) during school, weekdays after school and at weekends.

For peer review only - http://bmjopen.bmj.com/site/about/guidelines.xhtml

Participants will be asked to wear a wrist-worn Axivity AX3 monitor at T1, T3 and T4. Participants will be asked to wear the monitors on a strap on their non-dominant wrist, continuously for seven consecutive days, (including when in water and when asleep). Wrist-worn monitors have been validated for use among children and adolescents, in laboratory and free-living environments, and to assess physical activity, sedentary time and postural allocation.[28–30] There is evidence to support the increased acceptability and higher compliance rates of wrist-worn monitors compared to waist-worn monitors.[31–37] To further optimise accelerometer-wear compliance, we have developed a monitor wear and return protocol which is led by researchers (and not teachers), and includes regular reminders and an incentive (e.g. GoActive-branded headphones, GoActive branded pens). We have previously successfully applied this protocol in adolescent cohort studies to obtain high levels of valid accelerometry data (ROOTS: 825/930- 89%[7]; SPEEDY-3: 428/480 - 89%[16]).

Throughout data collection, we will continuously monitor response rates and take appropriate action (e.g. requesting teacher involvement) if it drops below 70% for the primary outcome. In cases where participants do not return their accelerometer after frequent requests, they may not be issued a monitor at subsequent measurements, but will be allowed to continue their participation in the study and all other (secondary) measures. This is to prevent excessive monitor loss. We deem this appropriate as sample size calculations indicate that we will retain 95% power should retention drop to 55% (80/150 participants predicted to participate in each school based on pilot data).

Once returned, data (continuous waveform data) from the accelerometers will be downloaded. Non-wear time with a minimum duration of 60 minutes will be removed; the acceleration threshold for identifying non-worn time will be based on visual inspection of the data...[38,39] As we will use a 24-hour protocol, we plan to apply a diurnal adjustment to reduce any bias that may occur if data was not fully representative of a 24 hour period but will also allow full use of the data collected.[40] For any daily analysis, we will set minimum criteria to ensure hours are equally distributed across whole day.[40]

Continuous waveform data will be converted to be comparable to cut-points used previously for ActiGraph accelerometers used to classify time spent sedentary (equivalent to ≤100 ActiGraph cpm), or in light (equivalent to 101 - 1999 ActiGraph cpm), moderate-vigorous (equivalent to ≥2000 ActiGraph cpm) or appropriate vector magnitude equivalents.[41–43] Monitor output will be reviewed prior to analysis to confirm that these decisions are appropriate for the population and monitor applied. Further, we will consult physical activity measurement experts to ensure we can be aware of relevant new methodology and apply where appropriate. Algorithms to identify sleep time are constantly in development. Given that we are operating a 24 hour wear time protocol, we will use the most up to date sleep identification algorithms to remove sleep time when estimating physical activity intensities (particularly sedentary time).

Anthropometry

Trained staff will measure height, weight and waist circumference following standardised operating procedures (e.g. wearing light clothing, removing shoes). Age- and sex-specific body fat percentage will be calculated from bio-electrical impedance (collected using Tanita TBF 300 scales), age- and sex-specific BMI z-score will be calculated from height and weight. Quality checking of researchers' anthropometry measurements will be conducted prior to baseline measurements and before 10-month follow-up.

For peer review only - http://bmjopen.bmj.com/site/about/guidelines.xhtml

Questionnaires

At each measurement session (i.e. T1, T2, T3, and T4), participants will complete a questionnaire concerning secondary outcomes, potential mediators or moderators, and items to monitor any adverse intervention effects. Physical activity type will be assessed using the 30-item Youth Physical Activity Questionnaire (YPAQ), which has previously been validated in 12-17 year olds.[44] Self-efficacy[45] and social support for physical activity[46] will be assessed using two scales (each with 3 items). Further items include friendship quality (8-item Cambridge Friendships Questionnaire),[47] well-being (14-item Edinburgh-Warwick Wellbeing Scale)[48] self-esteem (10- item Rosenberg Self Esteem Scale)[49], and an adapted social network modelling tool in which participants provided with a list of tutor group members and asked to select names of their friends)[50], and shyness and sociability (two 5-item measures from EAS temperament scale).[51] Questionnaires will be checked for completion before the end of the measurement sessions, and participants will be asked to complete any missing items. At T1, participants will respond to additional items providing demographic data (i.e. age, sex, ethnicity, language spoken at home, parent education, and family socio-economic status). School-level attendance and academic performance (from National Pupil Database) will be collected (publicly available data).

Process evaluation

Process evaluation will examine the proposed logic model for the GoActive intervention (Supplementary File 1). Intervention process data will include mixed-methods assessment of student, mentor and teacher experiences, and perspectives on intervention delivery, feasibility, acceptance, and barriers/facilitators to participation. Uptake (e.g. how many students participate in GoActive activities), dose (e.g. how often students download QuickCards), and maintenance (e.g. whether students continue to upload points to the website throughout the intervention) will be established using the points entries on the study website, download statistics for intervention materials and mentor-reported participation. Process questionnaires will be administered at T2 and T3 for (both intervention and control) participants, mentors, and form teachers. Control participants will also be asked to complete process questionnaires to determine possible contamination.[52] We will include a GoActive logbook for the intervention facilitator and mentors to assess frequency of intervention delivery. Given the flexible, spontaneous and informal nature of the intervention (mentors/leaders attend the same school and can therefore encourage/motivate Year 9 students at any time during the week), observation of intervention delivery is not deemed feasible. However, existing and emerging school practices which may affect students' physical activity behaviour will be documented and monitored in a structured manner using an adapted school environment questionnaire.[53]

A qualitative researcher will conduct semi-structured focus groups after the facilitated intervention phase (T2) with representatives from all relevant groups (Year 9 students, mentors, and teachers) in each intervention school. Each focus group (separate for students, mentors, and teachers) will comprise 3-8 individuals, with up to two facilitators. Students will be purposively sampled to ensure a mix of sex and ethnicity, and grouped by level of participation and physical activity. A topic guide will be developed and updated as new issues and themes emerge; participants will be encouraged to discuss additional issues. Issues arising will be incorporated into the next round of questionnaires and subsequent focus groups, so that additional mechanisms of change can be investigated. In addition to focus groups, interviews will be conducted with a

For peer review only - http://bmjopen.bmj.com/site/about/guidelines.xhtml

purposive sample of inactive and shy participants (identified using questionnaire data) at intervention schools to provide a deeper understanding of their intervention experience, and barriers and facilitators to participation (we anticipate these individuals will be more comfortable participating in one-to-one interviews).

At T4, additional semi-structured focus groups and interviews with students will explore maintenance of physical activity behaviour change, including who did or did not maintain physical activity behaviour change and why, whether GoActive helped and why or how, and other factors that helped or hindered physical activity maintenance. T2 participants will be re-invited, supplemented by additional students if needed. This gives us a unique opportunity to explore physical activity maintenance across time in the context of a trial, and to better understand barriers and facilitators to physical activity maintenance.

Cost-effectiveness

We will conduct both a within-trial and decision-model based economic evaluation. The within-trial analysis will be from the cost perspective of the school/local authority. Cost data collected will include intervention-related facilitator time, travel, and expenses collected by schools/researchers. Outcomes will comprise change in MVPA and quality adjusted life years (QALYs) gained. These will be assessed using the CHU-9D[54] and converted to health state utilities using UK specific valuations.[55] Change in physical activity observed and costs to schools/local authorities will be input into a previously developed model to predict longer term costs (to the National Health Service (NHS)) and QALYS hence cost-effectiveness from a public sector perspective (defined as local authority and NHS).

Data collection forms and questionnaires for all measurements are available on request from the corresponding author.

Data management and monitoring

All data will be collected and managed in line with International Conference on Harmonisation Good Clinical Practice guidelines. Real time entry and retrospective data validation checks will be conducted. All paper based questionnaire data will be professionally double data entered and a sample verified for accuracy. Data will be stored securely at the MRC Epidemiology Unit, Cambridge, UK. The MRC Epidemiology Unit specialist teams will provide support for training, and quality assessment and control of measurements, and this support will ensure that collection, processing, protection and management of data is timely and of high quality. We will ensure that all provided data are treated as confidential and stored securely. Where this is electronic, data are held on secure computer systems with at minimum password access. All identifiable data will be held on a separate computer system with access limited to appropriate staff by group and password permissions. Personal data will be stored and accessed up to 20 years after study completion.

Due to the low risk nature of the trial, a formal data monitoring committee has not been appointed. However, the Trial Steering Committee (TSC) will receive regular reports from the investigators and will monitor trial progress and conduct. The TSC will consist of an independent chair, one independent expert, two lay representatives (including a representative from educational sector) and at least two investigators; the committee will be at least 75% independent. The study coordinator and a sponsor representative will be invited as observers. The TSC will meet approximately once per year, or more frequently if needed. The TSC

For peer review only - http://bmjopen.bmj.com/site/about/guidelines.xhtml

is responsible for communicating any issues of concern to the Sponsor, specifically where the integrity of the study or data or patient safety could be comprised. The study coordinator will also monitor trial conduct and will report independently to the MRC Epidemiology Unit Clinical Research Manager. Potential harms will be monitored by the study team. These will be reviewed by the Study Coordinator, Principal Investigator, and Trial Steering Committee, and will include reported adverse events (e.g. injuries or psychological indicators such as well-being). While we do not expect harm as a result of the GoActive intervention or this trial, it is insured by the University of Cambridge who would provide compensation in case of harm.

The council-funded intervention facilitators will work closely with mentors and research staff to monitor protocol adherence. Poor adherence will be discussed with the research team and TSC, and strategies will be put in place where necessary. No activities are prohibited during the trial as students are expected to do their normal physical activities, including school PE.

Any protocol amendments will be proposed to the TSC and subsequently altered if necessary before submission to funder (NIHR) for approval. Protocol updates will then be uploaded to the NIHR website and trial registry if relevant.

Analyses

Sample size

We aim to detect a 5-minute difference in change in MVPA per day at 10-month follow-up, as observed in the pilot study.[26] A 5-minute increase is relevant at population level, as it would increase the proportion of adolescents meeting the guidelines of 60 minutes of MVPA per day from 43% to 50% (based on baseline pilot data), with significant impact on population health.[2] To estimate the required sample size, the following parameters have been used: power=85%, significance level=5%, standard deviation=17.8 (observed in the GoActive pilot),[26] intraclass correlation coefficient=0.034 (observed in SPEEDY-3, N=57 schools),[43] correlation between baseline and follow-up MVPA=0.59 (observed in GoActive pilot, to account for adjustment for baseline MVPA),[26] and average cluster size=100. Based on these parameters, we estimate N=1310 participants will be required for the primary effectiveness analysis. To account for potential school dropout and an estimated loss-to-follow-up of 30-40%, we aim to recruit 16 schools with 150 participants (total N=2400; average recruitment per school in pilot=154).[26] Should a school have more than 150 students in Year 9, we will include all those who assent to measurement.

Quantitative analyses

The primary analysis of effectiveness, intermediate, and safety outcomes will use an Intention To Treat (ITT) population, which includes all participants in the group to which they were randomised, regardless of the intervention received. A secondary analysis of efficacy and intermediate outcomes will use a Per Protocol (PP) population. Inclusion in the PP population will be based on the degree of usage of the intervention website and/or submission of points, and will be defined once clean data are available (but before the start of any trial analyses), when the distributions of degree of website usage can be inspected.

Outcome analyses

For peer review only - http://bmjopen.bmj.com/site/about/guidelines.xhtml

The primary efficacy outcome, MVPA, will be compared between intervention and control groups using analysis of covariance (ANCOVA), with adjustment for baseline MVPA; robust standard errors will be calculated to allow for the non-independence of individuals within each school. Where baseline values of MVPA are missing, the missing indicator method will be used to enable these participants to be included in the analysis.[56] An estimate of the intervention effect, 95% confidence interval, and p-value will be calculated. A similar method will be used for the secondary efficacy outcomes. School-level data will also enable analysis of key differences between those participating in the evaluation, and the wider school population; for example, patterns of non-response by demographic variables will be explored. Subgroup analyses by pre-specified moderators (sex, socio-economic status, ethnicity, baseline activity level, weight status) will be performed for the primary outcome only. The interaction between randomised group and each moderator will be tested, and if the p-value is <0.05, the intervention effect (difference between intervention and control, and 95% confidence interval) will be estimated within each subgroup. The effect on potential mediating variables will initially be assessed as described above. We will subsequently conduct formal mediation analyses using the product of coefficient method[57] to assess the underlying causal pathways of the intervention.

Qualitative analyses

Focus groups and interviews will be audio recorded, transcribed verbatim and made anonymous. Data will be analysed using constant comparative analysis, facilitated by QSR NVivo. Coding will be inductive, incorporating emerging themes as well as topics presented a priori in the topic guide. Initial analyses will inform future data collection and analysis. Interim themes will be discussed by the research team to reach consensus.

Cost-effectiveness analyses

Cost-effectiveness analyses will follow standardised protocols.[58] The main economic outcome will be the incremental cost-effectiveness ratio, expressed as incremental costs per incremental change in physical activity (MVPA) and per QALY gained (based on CHU-9D) for the trial period (including follow-up). Data collected will include intervention time, travel, expenses, resource use, and study-specific costs. In addition, if GoActive increases physical activity, this should reduce adult chronic disease via changes in weight or BMI, and blood glucose. To establish whether GoActive could increase length and/or quality of life and at what cost, it is not practical to conduct lifetime follow-up, therefore we propose adjusting an existing decision-analytic model to estimate the impact of physical activity on disease risk, quality-adjusted life expectancy (QALY) and cost to the NHS. The modelled analysis will therefore be from a public sector perspective (schools/local authority and NHS).

Further analyses

Further research questions can be addressed using the cohort data, including (but not limited to) assessment of the predictors of activity maintenance, and the longitudinal association between physical activity/sedentary behaviour and a) academic performance; b) shyness and sociability; and c) friendship quality. All proposed analyses will be approved by the project group, and authorship of manuscripts will be informed by recommended guidelines.[59]

For peer review only - http://bmjopen.bmj.com/site/about/guidelines.xhtml

Wider dissemination

If successful, it would be appropriate to disseminate this programme to schools and councils across the UK (in addition to peer-reviewed publications). Towards the end of the project, a deliberative dialogue workshop will be held with key stakeholders including students, parents, teachers, school governors, and representatives from local/national government. This final workshop will focus on plans for dissemination of results, and will include discussion of the process of programme adaptation to a diverse range of secondary schools and further ways of ensuring long-term appeal for adolescents. We anticipate that dissemination could be facilitated through the study website, hosting intervention materials (including videos) and study information.

Discussion

Given the lack of rigorously evaluated interventions, the results of a CRCT of the effectiveness and cost-effectiveness of GoActive are expected to add substantially to the limited evidence on adolescent physical activity promotion. This study will include an objective, wrist-worn measure of physical activity, aligning with contemporary population surveillance studies[60–62] and ensuring greater protocol compliance for enhanced data retention and quality.[32,34,35] Achieving sustained health behaviour change is an established priority,[10] and so the inclusion of medium to long-term follow-up of participants will enable conclusions regarding the trajectories of change (in particular, whether any initial behaviour change is maintained). It will also form one of the largest cohorts in the field of adolescent physical activity promotion, providing many opportunities for secondary data analysis, in addition to testing causal pathways of effect and examining cost-effectiveness. Irrespective of study outcome, the evaluation of the GoActive intervention to increase physical activity in adolescents has the potential for significant academic impact.

Contributors: The PI, KC will have overall responsibility for project progress and direction; HB will be the day-to-day scientific lead for the project and FW will be the operational lead. EvS, PW and AV will advise on study procedures and evaluation from their respective disciplines; PW will additionally lead the design and evaluation of psychosocial outcomes. CC will lead the qualitative and mixed methods research. EW will lead the economic evaluation. Study sponsor and funders will have no role in the study design, collection, management, analysis, and interpretation of data, writing of the report; and the decision to submit the report for publication.

Funding

This project is funded by the National Institute for Health Research Public Health Research (13/90/18). Intervention delivery costs will be borne by Essex and Cambridgeshire County Councils.

For peer review only - http://bmjopen.bmj.com/site/about/guidelines.xhtml

Figure 1: Measurement sessions included in the GoActive evaluation

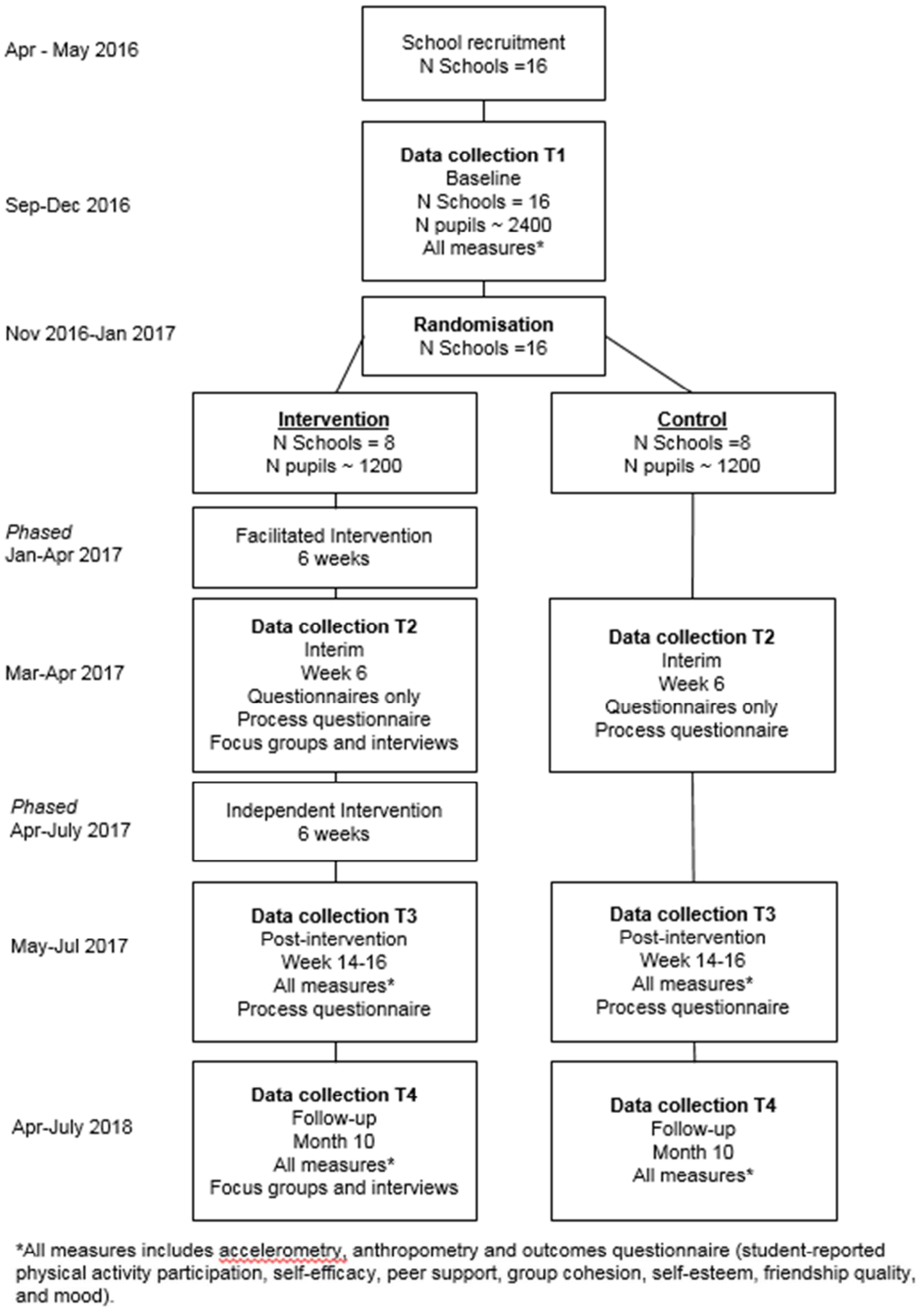

*All measures includes accelerometry, anthropometry and outcomes questionnaire (student-reported physical activity participation, self-efficacy, peer support, group cohesion, self-esteem, friendship quality, and mood).

For peer review only - http://bmjopen.bmj.com/site/about/guidelines.xhtml

Data sharing statement

After publication of trial analyses, and pending review of data access proposals by the investigators, data will be available on request from the corresponding author. The Principal Investigator, Co-Investigators, Statistician and Chair of the TSC will have access to the final trial dataset prior to conduct of the trial analyses. Statistical code for trial analysis will be available on request from the corresponding author.

There are no competing interests reported.

References

1 Janssen I, LeBlanc AG. Systematic review of the health benefits of physical activity and fitness in school-aged children and youth. Int. J. Behav. Nutr. Phys. Act. 2010;**7**:40.

2 Ekelund U, Luan J, Sherar LB, *et al.* Moderate to Vigorous Physical Activity and Sedentary Time and Cardiometabolic Risk Factors in Children and Adolescents. *JAMA J Am Med Assoc* 2012;**307**:704–12.

3 Dumith SC, Gigante DP, Domingues MR, *et al.* Physical activity change during adolescence: a systematic review and a pooled analysis. *Int J Epidemiol* 2011;**40**:685–98.

4 Raustorp A, Ekroth Y. Tracking of pedometer-determined physical activity: a 10-year follow-up study from adolescence to adulthood in Sweden. *J Phys Act Heal* 2013;**10**:1186–92.

5 Gill JMR, Cooper AR. Physical activity and prevention of type 2 diabetes mellitus. *Sports Med* 2008;**38**:807–24.

6 Khaw K-T, Wareham N, Bingham S, *et al.* Combined impact of health behaviours and mortality in men and women: the EPIC-Norfolk prospective population study. *PLoS Med* 2008;**5**:e12.

7 Corder K, Sharp SJ, Atkin AJ, *et al.* Change in objectively measured physical activity during the transition to adolescence. *Br J Sports Med* 2015;**49**:730–6.

8 Viner RM, Ozer EM, Denny S, *et al.* Adolescence and the social determinants of health. *Lancet* 2012;**379**:1641–52.

9 Department of Health. Annual Report of the Chief Medical Officer 2012: ‘Our Children Deserve Better: Prevention Pays’. 2013. https://www.gov.uk/government/uploads/system/uploads/attachment_data/file/255237/2901304_CMO_complete_low_res_accessible.pdf

10 Gillis L, Tomkinson G, Olds T, *et al.* Research priorities for child and adolescent physical activity and sedentary behaviours: an international perspective using a twin-panel Delphi procedure. *Int J Behav Nutr Phys Act* 2013;**10**:112.

11 Report: Tackling Physical Inactivity— A Coordinated Approach | All-Party Commission on Physical Activity on WordPress.com. https://activitycommission.com/report-tackling-physical-inactivity-a-coordinated-approach/ (accessed 21 Jul2016).

12 Van Sluijs EMF, McMinn AM, Griffin SJ. Effectiveness of interventions to promote physical activity in children and adolescents: systematic review of controlled trials. *BMJ* 2007;**335**:703.

13 Kriemler S, Meyer U, Martin E, *et al.* Effect of school-based interventions on physical activity and fitness in children and adolescents: a review of reviews and systematic update. *Br J Sports Med* 2011;**45**:923–30.

For peer review only - http://bmjopen.bmj.com/site/about/guidelines.xhtml

14 Metcalf B, Henley W, Wilkin T. Effectiveness of intervention on physical activity of children: systematic review and meta-analysis of controlled trials with objectively measured outcomes (EarlyBird 54). *BMJ* 2012;**345**:e5888.

15 Dobbins M, DeCorby K, Robeson P, *et al.* School-based physical activity programs for promoting physical activity and fitness in children and adolescents aged 6-18. *Cochrane Database Syst Rev* Published Online First: 2009.http://www.scopus.com/inward/record.url?eid=2-s2.0-70249099867&partnerID=40&md5=1ad81652911cae49189a325a9acf1c25

16 Corder K, Atkin AJ, Ekelund U, *et al.* What do adolescents want in order to become more active? *BMC Public Health* 2013;**13**:718.

17 Jago R, Edwards MJ, Sebire SJ, *et al.* Bristol girls dance project (BGDP): Protocol for a cluster randomised controlled trial of an after-school dance programme to increase physical activity among 11--12 year old girls. BMC Public Health. 2013;:1003.

18 Smith J, Morgan P, Plotnikoff R, *et al.* Smart-phone obesity prevention trial for adolescent boys in low-income communities: The ATLAS RCT. *Pediatrics* 2014;**134**:e723–31.

19 Dobbins M, Husson H, DeCorby K, *et al.* School-based physical activity programs for promoting physical activity and fitness in children and adolescents aged 6 to 18. *Cochrane database Syst Rev* 2013;**2**:CD007651.

20 Lonsdale C, Rosenkranz RR, Peralta LR, *et al.* A systematic review and meta-analysis of interventions designed to increase moderate-to-vigorous physical activity in school physical education lessons. Prev. Med. (Baltim). 2013;**56**:152–61.

21 Van Sluijs EMF, Kriemler S. Reflections on physical activity intervention research in young people - dos, don'ts, and critical thoughts. *Int J Behav Nutr Phys Act* 2016;**13**:25.

22 Corder K, Schiff A, Kesten JM, *et al.* Development of a universal approach to increase physical activity among adolescents: the GoActive intervention. *BMJ Open* 2015;**5**:e008610.

23 Chan AW, Tetzlaff JM, Altman DG, *et al.* SPIRIT 2013 statement: Defining standard protocol items for clinical trials. Ann. Intern. Med. 2013;**158**:200–7.

24 Chan AW, Tetzlaff JM, Altman DG, *et al.* SPIRIT 2013: New guidance for content of clinical trial protocols. Lancet. 2013;**381**:91–2.

25 Chan A-W, Tetzlaff JM, Gøtzsche PC, *et al.* SPIRIT 2013 explanation and elaboration: guidance for protocols of clinical trials. *BMJ* 2013;**346**:e7586.

26 Corder, Kirsten; Brown, Helen Elizabeth; Schiff, Anne;, van Sluijs EM. Feasibility study and pilot cluster randomised controlled trial of the GoActive Intervention aiming to promote physical activity among adolescents: outcomes and lessons learnt. *BMJ Open*

27 Shochet IM, Dadds MR, Holland D, *et al.* The efficacy of a universal school-based program to prevent adolescent depression. *J Clin Child Psychol* 2001;**30**:303–15.

28 Rowlands A V, Olds TS, Hillsdon M, *et al.* Assessing sedentary behavior with the GENEActiv: introducing the sedentary sphere. *Med Sci Sports Exerc* 2014;**46**:1235–47.

29 Phillips LRS, Parfitt G, Rowlands A V. Calibration of the GENEA accelerometer for assessment of physical activity intensity in children. *J Sci Med Sport* 2013;**16**:124–8.

30 Schaefer CA, Nigg CR, Hill JO, *et al.* Establishing and evaluating wrist cutpoints for the GENEActiv accelerometer in youth. *Med Sci Sports Exerc* 2014;**46**:826–33.

For peer review only - http://bmjopen.bmj.com/site/about/guidelines.xhtml

31 Rowlands A V., Yates T, Olds TS, *et al.* Sedentary sphere: Wrist-worn accelerometer-brand independent posture classification. *Med Sci Sports Exerc* 2016;**48**:748–54.

32 Rowlands A V., Rennie K, Kozarski R, *et al.* Children's physical activity assessed with wrist- and hip-worn accelerometers. *Med Sci Sports Exerc* 2014;**46**:2308–16.

33 Rowlands A V., Fraysse F, Catt M, *et al.* Comparability of measured acceleration from accelerometry-based activity monitors. *Med Sci Sports Exerc* 2015;**47**:201–10.

34 Rosenberger ME, Buman MP, Haskell WL, *et al.* 24 Hours of Sleep, Sedentary Behavior, and Physical Activity with Nine Wearable Devices. *Med Sci Sports Exerc* 2015;:457–65.

35 Hildebrand M, Van Hees VT, Hansen BH, *et al.* Age group comparability of raw accelerometer output from wrist-and hip-worn monitors. *Med Sci Sports Exerc* 2014;**46**:1816–24.

36 Fairclough SJ, Noonan R, Rowlands A V., *et al.* Wear compliance and activity in children wearing wrist- and hip-mounted accelerometers. *Med Sci Sports Exerc* 2016;**48**:245–53.

37 Edwardson CLL, Rowlands A V., Bunnewell S, *et al.* Accuracy of Posture Allocation Algorithms for Thigh- and Waist-Worn Accelerometers. Med. Sci. Sports Exerc. 2016.

38 Mattocks C, Ness A, Leary S, *et al.* Use of accelerometers in a large field-based study of children: protocols, design issues, and effects on precision. *J Phys Act Health* 2008;**5 Suppl 1**:S98–111.

39 Riddoch CJ, Bo Andersen L, Wedderkopp N, *et al.* Physical activity levels and patterns of 9- and 15-yr-old European children. *Med Sci Sport Exerc* 2004;**36**:86–92.

40 Collings PJ, Wijndaele K, Corder K, *et al.* Levels and patterns of objectively-measured physical activity volume and intensity distribution in UK adolescents: the ROOTS study. *Int J Behav Nutr Phys Act* 2014;**11**:23.

41 Trost SG, Loprinzi PD, Moore R, *et al.* Comparison of accelerometer cut points for predicting activity intensity in youth. *Med Sci Sports Exerc* 2011;**43**:1360–8.

42 Van Sluijs EMF, Skidmore PML, Mwanza K, *et al.* Physical activity and dietary behaviour in a population-based sample of British 10-year old children: the SPEEDY study (Sport, Physical activity and Eating behaviour: environmental Determinants in Young people). *BMC Public Health* 2008;**8**:388.

43 Corder K, van Sluijs EMF, Ekelund U, *et al.* Changes in children's physical activity over 12 months: longitudinal results from the SPEEDY study. *Pediatrics* 2010;**126**:e926–35.

44 Corder K, van Sluijs EMF, Wright A, *et al.* Is it possible to assess free-living physical activity and energy expenditure in young people by self-report? *Am J Clin Nutr* 2009;**89**:862–70.

45 Saunders RP, Pate RR, Felton G, *et al.* Development of Questionnaires to Measure Psychosocial Influences on Children's Physical Activity. *Prev Med (Baltim)* 1997;**26**:241–7.

46 Ommundsen Y, Page A, Ku PW, *et al.* Cross-cultural, age and gender validation of a computerised questionnaire measuring personal, social and environmental associations with children's physical activity: The European Youth Heart Study. *Int J Behav Nutr Phys Act* 2008;**5**:29.http://www.ncbi.nlm.nih.gov/entrez/query.fcgi?cmd=Retrieve&db=PubMed&dopt=Citation&list_uids=18489736

47 Goodyer IM, Herbert J, Tamplin a, *et al.* Short-term outcome of major depression: II. Life events, family dysfunction, and friendship difficulties as predictors of persistent disorder. *J Am Acad Child Adolesc Psychiatry* 1997;**36**:474–80.

For peer review only - http://bmjopen.bmj.com/site/about/guidelines.xhtml

48 Clarke A, Friede T, Putz R, *et al.* Warwick-Edinburgh Mental Well-being Scale (WEMWBS): validated for teenage school students in England and Scotland. A mixed methods assessment. *BMC Public Health* 2011;**11**:487.

49 Rosenberg M. *Society and the adolescent self-image*. NJ: Princeton: : Princeton University Press 1965.

50 Eys M, Loughead T, Bray SR, *et al.* Development of a cohesion questionnaire for youth: the Youth Sport Environment Questionnaire. *J Sport Exerc Psychol* 2009;**31**:390–408.

51 Buss AH, Plomin R. *Temperament: Early developing personality traits*. 1984.

52 Salmon J, Jorna M, Hume C, *et al.* A translational research intervention to reduce screen behaviours and promote physical activity among children: Switch-2-Activity. *Health Promot Int* 2011;**26**:311–21.

53 Wyatt KM, Lloyd JJ, Abraham C, *et al.* The Healthy Lifestyles Programme (HeLP), a novel school-based intervention to prevent obesity in school children: study protocol for a randomised controlled trial. *Trials* 2013;**14**:95.

54 Stevens K. Assessing the performance of a new generic measure of health related quality of life for children and refining it for use in health state valuation. *Appl Heal Econ Heal Policy* 2011;**9**:157–69.

55 Stevens K. Valuation of the child health utility 9D index. *Pharmacoeconomics* 2012;**30**:729–47.

56 White IR, Thompson SG. Adjusting for partially missing baseline measurements in randomized trials. *Stat Med* 2005;**24**:993–1007.

57 MacKinnon DP, Fairchild AJ, Fritz MS. Mediation analysis. *Annu Rev Psychol* 2007;**58**:593–614.

58 Drummond MF, Stoddard GL, Torrance GW. Methods for the economic evaluation of health care programmes. Methods Econ. Eval. Heal. Care Program. 1988;:39–71.

59 ICMJE. Recommendations: Defining the Role of Authors and Contributors. http://www.icmje.org/recommendations/browse/roles-and-responsibilities/defining-the-role-of-authors-and-contributors.html (accessed 9 Aug2016).

60 Troiano R MCJ. Objective Measures of Physical Activity, Sleep, and Strength in U.S. National Health and Nutrition Examination Survey (NHANES) 2011-2014. *8th Intern Conf Diet Act Methods*

61 Mannini A, Intille SS, Rosenberger M, *et al.* Activity recognition using a single accelerometer placed at the wrist or ankle. *Med Sci Sports Exerc* 2013;**45**:2193–203.

62 Sudlow C, Gallacher J, Allen N, *et al.* UK biobank: an open access resource for identifying the causes of a wide range of complex diseases of middle and old age. *PLoS Med* 2015;**12**:e1001779.

For peer review only - http://bmjopen.bmj.com/site/about/guidelines.xhtml

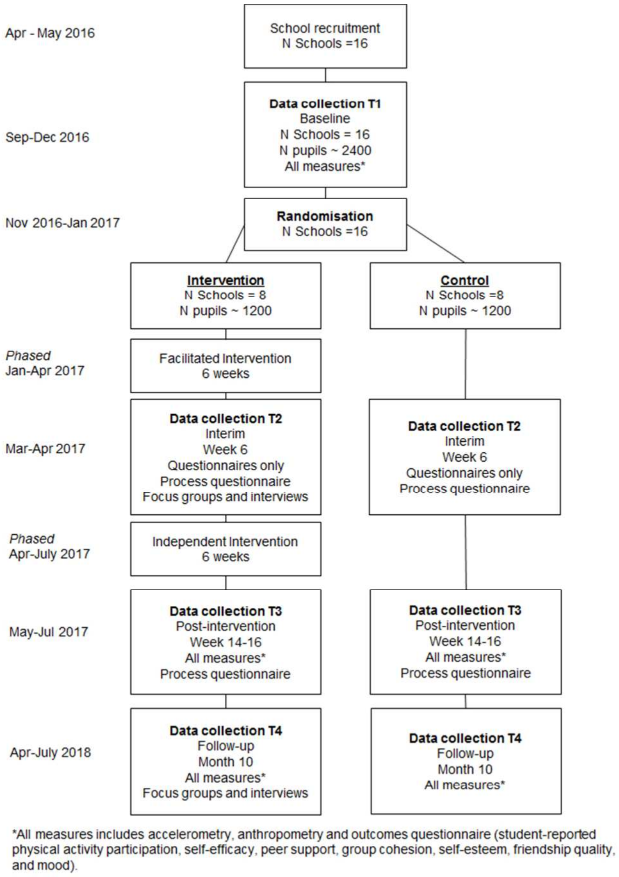

*All measures includes accelerometry, anthropometry and outcomes questionnaire (student-reported physical activity participation, self-efficacy, peer support, group cohesion, self-esteem, friendship quality, and mood).

SPIRIT
STANDARD PROTOCOL ITEMS: RECOMMENDATIONS FOR INTERVENTIONAL TRIALS

SPIRIT 2013 Checklist: Recommended items to address in a clinical trial protocol and related documents*

Section/item	Item No	Description	Addressed on page number
**Administrative information**			
Title	1	Descriptive title identifying the study design, population, interventions, and, if applicable, trial acronym	1
Trial registration	2a	Trial identifier and registry name. If not yet registered, name of intended registry	2
	2b	All items from the World Health Organization Trial Registration Data Set (some overlap)	
		Primary Registry and Trial Identifying Number	2
		Date of Registration in Primary Registry	2
		Secondary Identifying Numbers	2
		Source(s) of Monetary or Material Support	2
		Primary Sponsor	2
		Secondary Sponsor(s)	2
		Contact for Public Queries	1
		Contact for Scientific Queries	1
		Public Title	2
		Scientific Title	1
		Countries of Recruitment	4

For peer review only - http://bmjopen.bmj.com/site/about/guidelines.xhtml

		Health Condition(s) or Problem(s) Studied	3
		Intervention(s)	3
		Key Inclusion and Exclusion Criteria	5
		Study Type	4
		Type of study (interventional or observational)	4
		Method of allocation (randomized/non-randomized)	5
		Masking (is masking used and, if so, who is masked)	5
		Assignment (single arm, parallel, crossover or factorial)	5
		Purpose	3
		Date of First Enrollment	4
		Target Sample Size	9
		Recruitment Status	4
		Primary Outcome(s)	6
		Key Secondary Outcomes	7
Protocol version	3	Date and version identifier	2
Funding	4	Sources and types of financial, material, and other support	2
Roles and responsibilities	5a	Names, affiliations, and roles of protocol contributors	1/11
	5b	Name and contact information for the trial sponsor	2
	5c	Role of study sponsor and funders, if any, in study design; collection, management, analysis, and interpretation of data; writing of the report; and the decision to submit the report for publication, including whether they will have ultimate authority over any of these activities	11

For peer review only - http://bmjopen.bmj.com/site/about/guidelines.xhtml

For peer review only

	5d	Composition, roles, and responsibilities of the coordinating centre, steering committee, endpoint adjudication committee, data management team, and other individuals or groups overseeing the trial, if applicable (see Item 21a for data monitoring committee)	11
**Introduction**			
Background and rationale	6a	Description of research question and justification for undertaking the trial, including summary of relevant studies (published and unpublished) examining benefits and harms for each intervention	3
	6b	Explanation for choice of comparators	6
Objectives	7	Specific objectives or hypotheses	3
Trial design	8	Description of trial design including type of trial (eg, parallel group, crossover, factorial, single group), allocation ratio, and framework (eg, superiority, equivalence, noninferiority, exploratory)	4
**Methods: Participants, interventions, and outcomes**			
Study setting	9	Description of study settings (eg, community clinic, academic hospital) and list of countries where data will be collected. Reference to where list of study sites can be obtained	4
Eligibility criteria	10	Inclusion and exclusion criteria for participants. If applicable, eligibility criteria for study centres and individuals who will perform the interventions (eg, surgeons, psychotherapists)	5
Interventions	11a	Interventions for each group with sufficient detail to allow replication, including how and when they will be administered	3
	11b	Criteria for discontinuing or modifying allocated interventions for a given trial participant (eg, drug dose change in response to harms, participant request, or improving/worsening disease)	NA
	11c	Strategies to improve adherence to intervention protocols, and any procedures for monitoring adherence (eg, drug tablet return, laboratory tests)	7
	11d	Relevant concomitant care and interventions that are permitted or prohibited during the trial	NA

For peer review only - http://bmjopen.bmj.com/site/about/guidelines.xhtml

Outcomes	12	Primary, secondary, and other outcomes, including the specific measurement variable (eg, systolic blood pressure), analysis metric (eg, change from baseline, final value, time to event), method of aggregation (eg, median, proportion), and time point for each outcome. Explanation of the clinical relevance of chosen efficacy and harm outcomes is strongly recommended	6-8
Participant timeline	13	Time schedule of enrolment, interventions (including any run-ins and washouts), assessments, and visits for participants. A schematic diagram is highly recommended (see Figure)	13
Sample size	14	Estimated number of participants needed to achieve study objectives and how it was determined, including clinical and statistical assumptions supporting any sample size calculations	9
Recruitment	15	Strategies for achieving adequate participant enrolment to reach target sample size	4
**Methods: Assignment of interventions (for controlled trials)**			
Allocation:			
Sequence generation	16a	Method of generating the allocation sequence (eg, computer-generated random numbers), and list of any factors for stratification. To reduce predictability of a random sequence, details of any planned restriction (eg, blocking) should be provided in a separate document that is unavailable to those who enrol participants or assign interventions	5
Allocation concealment mechanism	16b	Mechanism of implementing the allocation sequence (eg, central telephone; sequentially numbered, opaque, sealed envelopes), describing any steps to conceal the sequence until interventions are assigned	5
Implementation	16c	Who will generate the allocation sequence, who will enrol participants, and who will assign participants to interventions	5
Blinding (masking)	17a	Who will be blinded after assignment to interventions (eg, trial participants, care providers, outcome assessors, data analysts), and how	5/6
	17b	If blinded, circumstances under which unblinding is permissible, and procedure for revealing a participant's allocated intervention during the trial	5
**Methods: Data collection, management, and analysis**			

Data collection methods	18a	Plans for assessment and collection of outcome, baseline, and other trial data, including any related processes to promote data quality (eg, duplicate measurements, training of assessors) and a description of study instruments (eg, questionnaires, laboratory tests) along with their reliability and validity, if known. Reference to where data collection forms can be found, if not in the protocol	6-8
	18b	Plans to promote participant retention and complete follow-up, including list of any outcome data to be collected for participants who discontinue or deviate from intervention protocols	5/10
Data management	19	Plans for data entry, coding, security, and storage, including any related processes to promote data quality (eg, double data entry; range checks for data values). Reference to where details of data management procedures can be found, if not in the protocol	8
Statistical methods	20a	Statistical methods for analysing primary and secondary outcomes. Reference to where other details of the statistical analysis plan can be found, if not in the protocol	9
	20b	Methods for any additional analyses (eg, subgroup and adjusted analyses)	9
	20c	Definition of analysis population relating to protocol non-adherence (eg, as randomised analysis), and any statistical methods to handle missing data (eg, multiple imputation)	10
**Methods: Monitoring**			
Data monitoring	21a	Composition of data monitoring committee (DMC); summary of its role and reporting structure; statement of whether it is independent from the sponsor and competing interests; and reference to where further details about its charter can be found, if not in the protocol. Alternatively, an explanation of why a DMC is not needed	9
	21b	Description of any interim analyses and stopping guidelines, including who will have access to these interim results and make the final decision to terminate the trial	NA
Harms	22	Plans for collecting, assessing, reporting, and managing solicited and spontaneously reported adverse events and other unintended effects of trial interventions or trial conduct	9
Auditing	23	Frequency and procedures for auditing trial conduct, if any, and whether the process will be independent from investigators and the sponsor	9
**Ethics and dissemination**			

For peer review only - http://bmjopen.bmj.com/site/about/guidelines.xhtml

Research ethics approval	24	Plans for seeking research ethics committee/institutional review board (REC/IRB) approval	4
Protocol amendments	25	Plans for communicating important protocol modifications (eg, changes to eligibility criteria, outcomes, analyses) to relevant parties (eg, investigators, REC/IRBs, trial participants, trial registries, journals, regulators)	9
Consent or assent	26a	Who will obtain informed consent or assent from potential trial participants or authorised surrogates, and how (see Item 32)	5
	26b	Additional consent provisions for collection and use of participant data and biological specimens in ancillary studies, if applicable	NA
Confidentiality	27	How personal information about potential and enrolled participants will be collected, shared, and maintained in order to protect confidentiality before, during, and after the trial	8
Declaration of interests	28	Financial and other competing interests for principal investigators for the overall trial and each study site	11
Access to data	29	Statement of who will have access to the final trial dataset, and disclosure of contractual agreements that limit such access for investigators	8
Ancillary and post-trial care	30	Provisions, if any, for ancillary and post-trial care, and for compensation to those who suffer harm from trial participation	NA
Dissemination policy	31a	Plans for investigators and sponsor to communicate trial results to participants, healthcare professionals, the public, and other relevant groups (eg, via publication, reporting in results databases, or other data sharing arrangements), including any publication restrictions	11
	31b	Authorship eligibility guidelines and any intended use of professional writers	11
	31c	Plans, if any, for granting public access to the full protocol, participant-level dataset, and statistical code	9
**Appendices**			
Informed consent materials	32	Model consent form and other related documentation given to participants and authorised surrogates	5
Biological specimens	33	Plans for collection, laboratory evaluation, and storage of biological specimens for genetic or molecular analysis in the current trial and for future use in ancillary studies, if applicable	NA

For peer review only - http://bmjopen.bmj.com/site/about/guidelines.xhtml

*It is strongly recommended that this checklist be read in conjunction with the SPIRIT 2013 Explanation & Elaboration for important clarification on the items. Amendments to the protocol should be tracked and dated. The SPIRIT checklist is copyrighted by the SPIRIT Group under the Creative Commons "Attribution-NonCommercial-NoDerivs 3.0 Unported" license.

For peer review only

For peer review only - http://bmjopen.bmj.com/site/about/guidelines.xhtml

BMJ Open

Protocol for a cluster randomised controlled trial to evaluate the effectiveness and cost-effectiveness of the GoActive intervention to increase physical activity among 13-14 year-old adolescents

Journal:	*BMJ Open*
Manuscript ID	bmjopen-2016-014419.R2
Article Type:	Protocol
Date Submitted by the Author:	02-Mar-2017
Complete List of Authors:	Brown, Helen Elizabeth; University of Cambridge, MRC Epidemiology Unit and Centre for Diet and Activity Research<br>Whittle, Fiona; University of Cambridge, MRC Epidemiology Unit<br>Jong, Stephanie; University of Cambridge, MRC Epidemiology Unit and Centre for Diet and Activity Research<br>Croxson, Caroline; University of Oxford, Nuffield Department of Primary Care Health Sciences<br>Sharp, Stephen; University of Cambridge, MRC Epidemiology Unit<br>Wilkinson, Paul; University of Cambridge, Department of Psychiatry<br>Wilson, Edward; University of Cambridge, Cambridge Centre for Health Services Research<br>van Sluijs, Esther; University of Cambridge, MRC Epidemiology Unit and Centre for Diet and Activity Research<br>Vignoles, Anna; University of Cambridge, Faculty of Education<br>Corder, Kirsten; University of Cambridge, MRC Epidemiology Unit and Centre for Diet and Activity Research
**Primary Subject Heading**:	Public health
Secondary Subject Heading:	Epidemiology, Sports and exercise medicine
Keywords:	physical activity, promotion, intervention, adolescent, protocol, school

SCHOLARONE™
Manuscripts

Protocol for a cluster randomised controlled trial to evaluate the effectiveness and cost-effectiveness of the GoActive intervention to increase physical activity among 13-14 year-old adolescents

Helen Elizabeth Brown[1*], Fiona Whittle[1], Stephanie T. Jong[1], Caroline Croxson[2], Stephen J. Sharp[1], Paul Wilkinson[3], Edward CF Wilson[4], Esther MF van Sluijs[1], Anna Vignoles[5], Kirsten Corder[1]

[1]UKCRC Centre for Diet and Activity Research (CEDAR) *and* MRC Epidemiology Unit, University of Cambridge
[2]Nuffield Department of Primary Care Health Sciences, University of Oxford
[3]Department of Psychiatry, University of Cambridge
[4]Cambridge Centre for Health Services Research, University of Cambridge
[5]Faculty of Education, University of Cambridge
*Corresponding Author

Address for correspondence:
Dr Helen Elizabeth Brown
UK CRC Centre for Diet and Activity Research (CEDAR)
Department of MRC Epidemiology
University of Cambridge School of Clinical Medicine
Box 285 Institute of Metabolic Science
Cambridge Biomedical Campus
Cambridge
CB2 0QQ
Email: heb56@medschl.cam.ac.uk

Abstract
Introduction: Adolescent physical activity promotion is rarely effective, despite adolescence being critical for preventing physical activity decline. Low physical activity is likely to last into adulthood, increasing health risks. It aims to increase physical activity through increased peer support, self-efficacy, group cohesion, self-esteem and friendship quality, and is implemented using a tiered-leadership system. We previously established feasibility in 1 school and conducted a pilot randomised controlled trial (RCT) in 3 schools.

Methods and analysis: We will conduct a school-based cluster RCT (CRCT) in 16 secondary schools targeting all Year 9 students (N=2400). In 8 schools, GoActive will run for 2 terms: weekly facilitation support from a council-funded health trainer will be offered in Term 1, with more distant support in Term 2. Tutor groups choose 2 weekly activities, encouraged by older mentors and weekly peer-leaders. Students gain points for trying new activities; points are entered into a between-class competition. Outcomes will be assessed at baseline, interim (week 6), post-intervention (week 14-16), and 10-month follow-up (main outcome). The primary outcome will be change from baseline in daily accelerometer-assessed moderate-to-vigorous physical activity. Secondary outcomes include accelerometer-assessed activity intensities on weekdays/weekends; self-reported physical activity and psycho-social outcomes; cost-effectiveness

For peer review only - http://bmjopen.bmj.com/site/about/guidelines.xhtml

analyses; mixed methods process evaluation integrating information from focus groups and participation logs/questionnaires.

Ethics and dissemination: Ethical approval was gained from the Cambridge Psychology Research Ethics Committee. Given the lack of rigorously evaluated interventions, and the inclusion of objective measurement of physical activity, long-term follow-up, and testing of causal pathways, the results of a CRCT of the effectiveness and cost-effectiveness of GoActive are expected to add substantially to the limited evidence on adolescent physical activity promotion. Workshops will be held with key stakeholders including students, parents, teachers, and government representatives, to discuss plans for wider dissemination of the intervention.

Strengths and limitations of this study

- The GoActive evaluation study uses a cluster-randomised controlled trial design and includes objective measurement of physical activity, long-term follow-up, and testing of causal pathways, to rigorously assess the effectiveness and cost-effectiveness of the GoActive programme.
- We will recruit 16 secondary schools from both Essex and Cambridgeshire. Despite our purposive sampling of schools with varied socio-economic status, it is likely that participants may not be entirely representative of the wider UK population (particularly with regards to ethnicity).
- This manuscript reports in detail on the recruitment and randomisation procedures, gives an overview of the GoActive intervention, and describes the included measures and proposed analyses, in accordance with SPIRIT guidance. As the trial is currently underway, there are no results presented in this manuscript.

Trial registration number: ISRCTN31583496

NB: The pilot trial of the GoActive intervention was registered retrospectively. We attempted to add the full CRCT prospectively, but were unfortunately not allowed to submit this as a new ISRCTN record and so it was added to the pilot record (which remained 'retrospective').

Originally registered: 18/2/2014

Funding reference: NIHR-PHR 13/90/18

Intervention delivery costs will be borne by Essex and Cambridgeshire County Councils.

Sponsor: University of Cambridge, contact**:** Mrs Carolyn Read, University of Cambridge School of Clinical Medicine, Box 111 Cambridge Biomedical Campus, Cambridge, CB2 0SP, United Kingdom, cad50@medschl.cam.ac.uk

Contact for Public and Scientific Queries: As address for correspondence

Public title: To establish the effect of the GoActive programme to increase physical activity among 13-14 year-old (Year 9) adolescents.

Scientific title: A cluster randomised controlled trial to evaluate the effectiveness and cost-effectiveness of the GoActive programme to increase physical activity among 13-14 year-old adolescents.

Protocol version: 2.0

For peer review only - http://bmjopen.bmj.com/site/about/guidelines.xhtml

Keywords: physical activity, promotion, intervention, adolescent, health behaviour

For peer review only

For peer review only - http://bmjopen.bmj.com/site/about/guidelines.xhtml

Background

Physical activity is protective against obesity and related metabolic disorders in young people[1,2]. Meta-analytic data from 20,871 4-18 year olds suggest that every 10-minute increase in moderate-to-vigorous activity (MVPA) is associated with a smaller waist circumference (-0.52 cm) and lower fasting insulin (-0.028 pmol/L).[2] In adolescence however, physical activity declines 7% per year.[3] Low physical activity in adolescence is also likely to progress to adulthood inactivity,[4] increasing the risk of diabetes, cancer and mortality.[5,6] Adolescence is therefore a critical period to increase physical activity,[7] both due to the aforementioned decline and because pubertal, brain and social development during this time leads to new capacity for changing health behaviours,[8] increasing the likelihood of long term change.

The 2012 Chief Medical Officer's report states the importance of physical activity among young people[9] and a recent international expert panel concluded that developing effective and sustainable interventions to increase physical activity among young people is the most important priority in the physical activity research field.[10] Further, the recently published report from the All-Party Commission on Physical Activity calls specifically for the creation of active schools, including the provision of a more diverse and inclusive offer of physical activity.[11]

Reviews highlight the limited efficacy of existing adolescent physical activity promotion interventions.[12,13,14,15] We have previously identified several possible reasons for this lack of effectiveness[16]; for example, many interventions only target subgroups (such as girls[17] or low socio-economic groups[18]) despite activity declining among all groups.[16] We aim to recruit the whole school year group for evaluation, and to target all groups in the GoActive intervention, which to our knowledge has rarely been done in physical activity promotion interventions. In addition, the decline in activity mainly occurs out of school;[16] however, many interventions only target specific school-based times; for example, school time[13,19] or Physical Education lessons.[20] whereas GoActive encourages participants to do more activity both in and out of school. Further, very few adolescent physical activity interventions, especially among older adolescents, have been evaluated using objective measurement of physical activity,[14] and including long-term follow-up, process evaluation, or an assessment of cost-effectiveness.[21] This therefore highlights an urgent need for more rigorous evaluation of potentially effective strategies to increase physical activity in adolescents.

Objectives

The primary aim of this study is to assess the 10-month effectiveness of the GoActive intervention to increase average daily objectively measured MVPA among 13-14 year-old adolescents. We will also assess the effect of GoActive immediately post-intervention, and on the following secondary outcomes: a) objectively assessed activity intensities during school time, weekday evenings and weekends; b) student-reported physical activity participation, self-efficacy, peer support, social networks, self-esteem, friendship quality (proposed mediators), and wellbeing, and school-level attendance and academic performance and c) body composition (body fat percentage and body mass index (BMI) z-score). We will investigate potential moderation of intervention effects by sex, socio-economic status, ethnicity, baseline activity level, and weight status, and potential mechanisms of effect by proposed mediators using a mixed-methods approach. Further, we will assess short term (within-trial) and potential long term cost-effectiveness of the GoActive

For peer review only - http://bmjopen.bmj.com/site/about/guidelines.xhtml

intervention, and will conduct a comprehensive process evaluation including questionnaires, focus groups (with participants, mentors, and teachers), individual interviews, data from intervention logs, and website analytics.

Intervention

The development of the “GoActive” (Get Others Active) intervention with supporting rationale has been described in detail previously.[22] Briefly, each Year 9 class (tutor group or home room class) chooses two activities each week from a selection provided. There are currently 20 activities available, utilising little or no equipment, and appealing to a wide variety of students (including Ultimate Frisbee, Zumba and Hula Hoop). Materials available on the password-protected GoActive intervention website include activity instructions (Quick Cards) which offer an overview of each activity, a short explanation, suggestions for adaptations, and provide advice, safety tips and ‘factoids’, in addition to a short video introducing each activity. GoActive is implemented using a tiered-leadership system where mentors (older adolescents within the school) and peer-leaders (within each Year 9 class) encourage students to try these activities each week. The mentors remain paired with each class for the duration of the intervention, whereas the peer-leaders (two per class each week, one male and one female) change every week. In addition to the student leaders, a local authority-funded intervention facilitator will support the programme during the first term of delivery and will provide distant support thereafter.

Teachers are encouraged to use one tutor time weekly to do one of the chosen activities as a class, however, students gain points for trying these new activities at any time in or out of school. Points are gained every time they try an activity; there is no expectation of time spent doing the activity as points are rewarded for the taking part itself. Individual students keep track of their own points privately on the study website and their points are entered into the between-class competition. Class rankings are available on the website to encourage teacher support and students receive small rewards (such as a Frisbee, a t-shirt, or a drawstring sports bag) for reaching individual points thresholds.

Methods

Study design

We will conduct a school-based cluster randomised controlled trial (CRCT) of the GoActive intervention. The study will be conducted in government-funded, non-fee-paying (state), all-ability, co-educational secondary schools including Year 9 students in Cambridgeshire and Essex, UK. After baseline measurements (September - December 2016), schools will be randomly allocated to one of two conditions; (1) to deliver the GoActive intervention to the whole of Year 9, or (2) to a no-treatment control group. Participant data collection will occur at baseline, 6 weeks, 14-16 weeks and 10 months (primary outcome). The protocol will be conducted and reported in accordance with SPIRIT guidance (Standard Protocol Items: Recommendations for Interventional Trials).[23,24,25] The trial has been registered with the ISRCTN registry (trial registration number: ISRCTN31583496).

Ethical approval

For peer review only - http://bmjopen.bmj.com/site/about/guidelines.xhtml

Ethical approval for the conduct of the study was gained from the Cambridge Psychology Research Ethics Committee, who previously provided ethical approval for the development, feasibility and pilot studies following similar procedures. [22,26]

Recruitment procedures

Schools

We will recruit 16 secondary schools with a mixture of socio-economic status, representative of UK variability. Head teachers, Year 9 leaders, and Physical Education (PE) leaders from all eligible schools will be sent an invitation letter and school information sheet via email. These documents will describe the study procedures (e.g. student recruitment and consent, measurements), and will include an electronic link to an information video describing GoActive. A follow-up phone call to each school will be made approximately one week after the initial invitation, asking for a meeting with relevant staff to discuss the study and request consent to participate. Phone calls and repeat emails will continue until 16 schools (8 in Cambridgeshire, and 8 in Essex) have provided consent to participate. We will also create a waiting list to replace any schools who may withdraw from the study prior to randomisation. We will also use our existing networks and school contacts to facilitate school recruitment. Schools who do not agree to take part will be asked to select the most relevant reason for their refusal from a pre-determined list (e.g. lack of interest, lack of time).

Participants

All Year 9 students (13-14 year-olds) in participating schools will be eligible to participate in study measurements. As in feasibility and pilot work, we plan to include disabled participants and those with learning or movement difficulties, taking care to follow advice from schools.[26] This is appropriate due to the inclusive nature of the GoActive intervention, and will help to avoid stigmatisation of any groups within schools.[27] As such, no exclusion criteria will be applied.

All Year 9 students and their parents will receive a paper invitation pack, including a participant information sheet and an invitation to participate in study measurements. These information packs will be distributed to students during an introductory assembly conducted by a member of the GoActive team; students will be asked to take the packs home to their parents. Parents will also be sent duplicate information via email ('ParentMail' or the appropriate equivalent system as agreed by the school). Parents will be asked to provide passive consent (active opt-out consent) for their child to take part in study measurements. We will give parents at least two weeks to respond (a final date for response will be included in all correspondence). After one week, parents will receive an additional copy to ensure further opportunity for opting out prior to study measurements. Parents will be given the option to phone or email the study team (in lieu of returning a form) to facilitate their ability to respond. Reminders will additionally be included in all relevant school media, including regular newsletters sent from the school. Written assent will be obtained from the students by research assistants trained in Good Clinical Practice prior to any baseline measurements taking place. Consent forms will be available on the study website www.goactive-uk.com after ethical approval for the trial has been obtained. Mentors and teachers will provide written consent or assent (for those older and younger than 16 years, respectively) to participate in process evaluation following the same procedures as study participants.

For peer review only - http://bmjopen.bmj.com/site/about/guidelines.xhtml

Parental opt-out responses ranged from 2 (<1%) to 18 (7%) in feasibility and pilot schools with 72-88% of eligible students assenting to participate.[26] Recruitment rates using this strategy are substantially higher than previous UK-based research in this age group using parental opt-in consent (23% of eligible participants).[7] Participants will be informed that they can discontinue all or any part of the study (either or both measurements and intervention) at any time at their or their parent/guardian's request.

School randomisation

Schools will be stratified based on Pupil Premium (proxy for socio-economic status, below/above the county-specific median; for information: https://www.gov.uk/guidance/pupil-premium-information-for-schools-and-alternative-provision-settings), and county (i.e. Cambridgeshire or Essex). Randomisation lists for each stratum will be prepared by a statistician, using Stata (ref: StataCorp. 2015. Stata Statistical Software: Release 14. College Station, TX: StataCorp LP), after baseline measurements are completed to ensure schools and participants are unaware of their group allocation at baseline. Eight schools will be randomised to deliver the GoActive intervention and eight to a no-treatment control condition. For measurements after randomisation, it will not be possible to blind participants to randomised allocation, as the intervention schools will have received the GoActive intervention.

Measurement staff will be blinded to intervention condition throughout the study, as they will be trained and work separately from those involved in intervention delivery. Process evaluation with measurement staff will examine the success of blinding.

Control condition

The control group will receive no-treatment or 'usual care', and no intervention will be implemented. If we were to offer the control group the intervention after follow-up measures, it would prevent us from potentially assessing longer-term impact of the programme. As such, this study has no wait-list control condition.

Data collection

Measurements will be conducted at four time points by trained researchers (Figure 1). The primary measure of intervention effectiveness will be change from baseline in accelerometer-measured average daily MVPA at 10-month follow-up. All primary and secondary outcomes will be assessed at T1 and T4. Anthropometric measures will be removed from T3 (which will include all other outcomes, i.e. accelerometry and questionnaire-based measures), and T2 will focus on assessing the questionnaire-based measures only (including mediators of change). To prevent artificially inflated school-level clustering (due to weather conditions or school events) and facilitate recruitment and retention, measurements at each school will be staggered over ≥2 weeks using a predetermined schedule.

Figure 1 near here

Accelerometry

The primary outcome will be accelerometer-assessed change in average daily MVPA between baseline and 10-month follow-up. Secondary accelerometry outcomes will be change from baseline in average minutes

For peer review only - http://bmjopen.bmj.com/site/about/guidelines.xhtml

spent in sedentary and light activity, as well as overall physical activity (counts per minute) during school, weekdays after school and at weekends.

Participants will be asked to wear a wrist-worn Axivity AX3 monitor at T1, T3 and T4. Participants will be asked to wear the monitors on a strap on their non-dominant wrist, continuously for seven consecutive days, (including when in water and when asleep). Wrist-worn monitors have been validated for use among children and adolescents, in laboratory and free-living environments, and to assess physical activity, sedentary time and postural allocation.[28,29,30] There is evidence to support the increased acceptability and higher compliance rates of wrist-worn monitors compared to waist-worn monitors.[31,32,33,34,35,36,37] To further optimise accelerometer-wear compliance, we have developed a monitor wear and return protocol which is led by researchers (and not teachers), and includes regular reminders and an incentive (e.g. GoActive-branded headphones, GoActive branded pens). We have previously successfully applied this protocol in adolescent cohort studies to obtain high levels of valid accelerometry data (ROOTS: 825/930- 89%[7]; SPEEDY-3: 428/480 - 89%[16]).

Throughout data collection, we will continuously monitor response rates and take appropriate action (e.g. requesting teacher involvement) if it drops below 70% for the primary outcome. In cases where participants do not return their accelerometer after frequent requests, they may not be issued a monitor at subsequent measurements, but will be allowed to continue their participation in the study and all other (secondary) measures. This is to prevent excessive monitor loss. We deem this appropriate as sample size calculations indicate that we will retain 95% power should retention drop to 55% (80/150 participants predicted to participate in each school based on pilot data).

Once returned, data (continuous waveform data) from the accelerometers will be downloaded. Non-wear time with a minimum duration of 60 minutes will be removed; the acceleration threshold for identifying non-worn time will be based on visual inspection of the data...[38,39] As we will use a 24-hour protocol, we plan to apply a diurnal adjustment to reduce any bias that may occur if data was not fully representative of a 24 hour period but will also allow full use of the data collected.[40] For any daily analysis, we will set minimum criteria to ensure hours are equally distributed across whole day.[40]

Continuous waveform data will be converted to be comparable to cut-points used previously for ActiGraph accelerometers used to classify time spent sedentary (equivalent to ≤100 ActiGraph cpm), or in light (equivalent to 101 - 1999 ActiGraph cpm), moderate-vigorous (equivalent to ≥2000 ActiGraph cpm) or appropriate vector magnitude equivalents.[41,42,43] Monitor output will be reviewed prior to analysis to confirm that these decisions are appropriate for the population and monitor applied. Further, we will consult physical activity measurement experts to ensure we can be aware of relevant new methodology and apply where appropriate. Algorithms to identify sleep time are constantly in development. Given that we are operating a 24 hour wear time protocol, we will use the most up to date sleep identification algorithms to remove sleep time when estimating physical activity intensities (particularly sedentary time).

Anthropometry

For peer review only - http://bmjopen.bmj.com/site/about/guidelines.xhtml

Trained staff will measure height, weight and waist circumference following standardised operating procedures (e.g. wearing light clothing, removing shoes). Age- and sex-specific body fat percentage will be calculated from bio-electrical impedance (collected using Tanita TBF 300 scales), age- and sex-specific BMI z-score will be calculated from height and weight. Quality checking of researchers' anthropometry measurements will be conducted prior to baseline measurements and before 10-month follow-up.

Questionnaires

At each measurement session (i.e. T1, T2, T3, and T4), participants will complete a questionnaire concerning secondary outcomes, potential mediators or moderators, and items to monitor any adverse intervention effects. Physical activity type will be assessed using the 30-item Youth Physical Activity Questionnaire (YPAQ), which has previously been validated in 12-17 year olds.[44] Self-efficacy[45] and social support for physical activity[46] will be assessed using two scales (each with 3 items). Further items include friendship quality (8-item Cambridge Friendships Questionnaire),[47] well-being (14-item Edinburgh-Warwick Wellbeing Scale)[48] self-esteem (10- item Rosenberg Self Esteem Scale)[49], and an adapted social network modelling tool in which participants provided with a list of tutor group members and asked to select names of their friends)[50], and shyness and sociability (two 5-item measures from EAS temperament scale).[51] Questionnaires will be checked for completion before the end of the measurement sessions, and participants will be asked to complete any missing items. At T1, participants will respond to additional items providing demographic data (i.e. age, sex, ethnicity, language spoken at home, parent education, and family socio-economic status). School-level attendance and academic performance (from National Pupil Database) will be collected (publicly available data).

Process evaluation

Process evaluation will examine the proposed action model for the GoActive intervention (Supplementary File 1). These process evaluation questions emulate those depicted in Saunders, Evans and Joshi's (2005) process-evaluation plan to assess the implementation of a targeted health promotion intervention.[52] We focus on six components: fidelity, dose (delivered and received), reach, recruitment and context.[52,53,54] Supplementary File 1 demonstrates the applicability, and operationalisation of these components.

Intervention process data will include mixed-methods assessment of student, mentor, facilitator, teacher, and GoActive staff experiences, and perspectives on intervention delivery, feasibility, acceptance, and barriers/facilitators to participation. Reach (e.g. the intended amount of students that participate within the intervention) and dose received (e.g. the proportion of students who enter points on the GoActive website, how often students download QuickCards and view videos) will be established using the points entries on the study website, download statistics for intervention materials and mentor-reported participation. Process questionnaires will be administered at T2 and T3 for (both intervention and control) students, mentors, facilitators, and form teachers. Control participants will be asked to complete process questionnaires to determine possible contamination. We will include a GoActive logbook for the intervention facilitator and mentors to assess frequency of intervention delivery and any other descriptive notes at T2. Given the flexible, spontaneous and informal nature of the intervention (mentors/leaders attend the same school and can therefore encourage/motivate Year 9 students at any time during the week), observation of all intervention delivery is not feasible; but classroom observation* will be undertaken to complement other

For peer review only - http://bmjopen.bmj.com/site/about/guidelines.xhtml

qualitative methods. Existing and emerging school practices which may affect students' physical activity behaviour will be documented and monitored in a structured manner using an adapted school environment questionnaire.[53]

A qualitative researcher will conduct semi-structured focus groups, using open-ended questions, after the facilitated intervention phase (T2) with Year 9 students in a sample of intervention schools. This sample will reflect variability of socio-economic status of the schools. Three Year 9 classrooms will be randomly selected to participate in the focus groups. Approximately 12 students will be selected to participate in the focus groups from all eligible children within the 3 classrooms. Each focus group will be comprised of approximately four individuals in order to develop themes and generate adequate data. Students will be purposively sampled to ensure a mix of sex and ethnicity, and grouped by level of participation in the GoActive intervention and physical activity. Subsequent interviews with representatives from all other relevant groups within intervention schools (mentors, teachers, and facilitators) will commence in T3. Each focus group (separate for mentors, teachers and facilitators) will comprise of 3 to 8 individuals. An interview guide will be developed and updated as new issues and themes emerge; participants will be encouraged to discuss additional issues. Issues arising will inform the next round of questionnaires and subsequent focus groups, so that additional mechanisms of change can be investigated. In addition to focus groups, individual interviews will be conducted with a purposive sample of inactive and shy participants (identified using questionnaire data) at intervention schools to provide a deeper understanding of their intervention experience, and barriers and facilitators to participation (we anticipate these individuals will be more comfortable participating in one-to-one interviews).

At T4, additional semi-structured focus groups and interviews with students will explore maintenance of physical activity behaviour change, including who did or did not maintain physical activity behaviour change and why, whether GoActive helped and why or how, and other factors that helped or hindered physical activity maintenance. T2 participants will be re-invited, supplemented by additional students if needed. This gives us a unique opportunity to explore physical activity maintenance across time in the context of a trial, and to better understand barriers and facilitators to physical activity maintenance.

Cost-effectiveness

We will conduct both a within-trial and decision-model based economic evaluation. The within-trial analysis will be from the cost perspective of the school/local authority. Cost data collected will include intervention-related facilitator time, travel, and expenses collected by schools/researchers. Outcomes will comprise change in MVPA and quality adjusted life years (QALYs) gained. These will be assessed using the CHU-9D[55] and converted to health state utilities using UK specific valuations.[56] Change in physical activity observed and costs to schools/local authorities will be input into a previously developed model to predict longer term costs (to the National Health Service (NHS)) and QALYS hence cost-effectiveness from a public sector perspective (defined as local authority and NHS).

Data collection forms and questionnaires for all measurements are available on request from the corresponding author.

For peer review only - http://bmjopen.bmj.com/site/about/guidelines.xhtml

Data management and monitoring

All data will be collected and managed in line with International Conference on Harmonisation Good Clinical Practice guidelines. Real time entry and retrospective data validation checks will be conducted. All paper based questionnaire data will be professionally double data entered and a sample verified for accuracy. Data will be stored securely at the MRC Epidemiology Unit, Cambridge, UK. The MRC Epidemiology Unit specialist teams will provide support for training, and quality assessment and control of measurements, and this support will ensure that collection, processing, protection and management of data is timely and of high quality. We will ensure that all provided data are treated as confidential and stored securely. Where this is electronic, data are held on secure computer systems with at minimum password access. All identifiable data will be held on a separate computer system with access limited to appropriate staff by group and password permissions. Personal data will be stored and accessed up to 20 years after study completion.

Due to the low risk nature of the trial, a formal data monitoring committee has not been appointed. However, the Trial Steering Committee (TSC) will receive regular reports from the investigators and will monitor trial progress and conduct. The TSC will consist of an independent chair, one independent expert, two lay representatives (including a representative from educational sector) and at least two investigators; the committee will be at least 75% independent. The study coordinator and a sponsor representative will be invited as observers. The TSC will meet approximately once per year, or more frequently if needed. The TSC is responsible for communicating any issues of concern to the Sponsor, specifically where the integrity of the study or data or patient safety could be comprised. The study coordinator will also monitor trial conduct and will report independently to the MRC Epidemiology Unit Clinical Research Manager. Potential harms will be monitored by the study team. These will be reviewed by the Study Coordinator, Principal Investigator, and Trial Steering Committee, and will include reported adverse events (e.g. injuries or psychological indicators such as well-being). While we do not expect harm as a result of the GoActive intervention or this trial, it is insured by the University of Cambridge who would provide compensation in case of harm.

The council-funded intervention facilitators will work closely with mentors and research staff to monitor protocol adherence. Poor adherence will be discussed with the research team and TSC, and strategies will be put in place where necessary. No activities are prohibited during the trial as students are expected to do their normal physical activities, including school PE.

Any protocol amendments will be proposed to the TSC and subsequently altered if necessary before submission to funder (NIHR) for approval. Protocol updates will then be uploaded to the NIHR website and trial registry if relevant.

Analyses

Sample size

We aim to detect a 5-minute difference in change in MVPA per day at 10-month follow-up, as observed in the pilot study.[26] A 5-minute increase is relevant at population level, as it would increase the proportion of adolescents meeting the guidelines of 60 minutes of MVPA per day from 43% to 50% (based on baseline pilot data), with significant impact on population health.[2] To estimate the required sample size, the following

For peer review only - http://bmjopen.bmj.com/site/about/guidelines.xhtml

parameters have been used: power=85%, significance level=5%, standard deviation=17.8 (observed in the GoActive pilot),[26] intraclass correlation coefficient=0.034 (observed in SPEEDY-3, N=57 schools),[43] correlation between baseline and follow-up MVPA=0.59 (observed in GoActive pilot, to account for adjustment for baseline MVPA),[26] and average cluster size=100. Based on these parameters, we estimate N=1310 participants will be required for the primary effectiveness analysis. To account for potential school dropout and an estimated loss-to-follow-up of 30-40%, we aim to recruit 16 schools with 150 participants (total N=2400; average recruitment per school in pilot=154).[26] Should a school have more than 150 students in Year 9, we will include all those who assent to measurement.

Quantitative analyses

The primary analysis of effectiveness, intermediate, and safety outcomes will use an Intention To Treat (ITT) population, which includes all participants in the group to which they were randomised, regardless of the intervention received. A secondary analysis of efficacy and intermediate outcomes will use a Per Protocol (PP) population. Inclusion in the PP population will be based on the degree of usage of the intervention website and/or submission of points, and will be defined once clean data are available (but before the start of any trial analyses), when the distributions of degree of website usage can be inspected.

Outcome analyses

The primary efficacy outcome, MVPA, will be compared between intervention and control groups using analysis of covariance (ANCOVA), with adjustment for baseline MVPA; robust standard errors will be calculated to allow for the non-independence of individuals within each school. Where baseline values of MVPA are missing, the missing indicator method will be used to enable these participants to be included in the analysis.[57] An estimate of the intervention effect, 95% confidence interval, and p-value will be calculated. A similar method will be used for the secondary efficacy outcomes. School-level data will also enable analysis of key differences between those participating in the evaluation, and the wider school population; for example, patterns of non-response by demographic variables will be explored. Subgroup analyses by pre-specified moderators (sex, socio-economic status, ethnicity, baseline activity level, weight status) will be performed for the primary outcome only. The interaction between randomised group and each moderator will be tested, and if the p-value is <0.05, the intervention effect (difference between intervention and control, and 95% confidence interval) will be estimated within each subgroup. The effect on potential mediating variables will initially be assessed as described above. We will subsequently conduct formal mediation analyses using the product of coefficient method[58] to assess the underlying causal pathways of the intervention.

Qualitative analyses

Focus groups and interviews will be audio recorded, transcribed verbatim and made anonymous. Data will be analysed using constant comparative analysis, facilitated by QSR NVivo. Coding will be inductive, incorporating emerging themes as well as topics presented a priori in the topic guide. Initial analyses will inform future data collection and analysis. Interim themes will be discussed by the research team to reach consensus.

For peer review only - http://bmjopen.bmj.com/site/about/guidelines.xhtml

Cost-effectiveness analyses

Cost-effectiveness analyses will follow standardised protocols.[59] The main economic outcome will be the incremental cost-effectiveness ratio, expressed as incremental costs per incremental change in physical activity (MVPA) and per QALY gained (based on CHU-9D) for the trial period (including follow-up). Data collected will include intervention time, travel, expenses, resource use, and study-specific costs. In addition, if GoActive increases physical activity, this should reduce adult chronic disease via changes in weight or BMI, and blood glucose. To establish whether GoActive could increase length and/or quality of life and at what cost, it is not practical to conduct lifetime follow-up, therefore we propose adjusting an existing decision-analytic model to estimate the impact of physical activity on disease risk, quality-adjusted life expectancy (QALY) and cost to the NHS. The modelled analysis will therefore be from a public sector perspective (schools/local authority and NHS).

Further analyses

Further research questions can be addressed using the cohort data, including (but not limited to) assessment of the predictors of activity maintenance, and the longitudinal association between physical activity/sedentary behaviour and a) academic performance; b) shyness and sociability; and c) friendship quality. All proposed analyses will be approved by the project group, and authorship of manuscripts will be informed by recommended guidelines.[60]

Wider dissemination

If successful, it would be appropriate to disseminate this programme to schools and councils across the UK (in addition to peer-reviewed publications). Towards the end of the project, a deliberative dialogue workshop will be held with key stakeholders including students, parents, teachers, school governors, and representatives from local/national government. This final workshop will focus on plans for dissemination of results, and will include discussion of the process of programme adaptation to a diverse range of secondary schools and further ways of ensuring long-term appeal for adolescents. We anticipate that dissemination could be facilitated through the study website, hosting intervention materials (including videos) and study information.

Discussion

Given the lack of rigorously evaluated interventions, the results of a CRCT of the effectiveness and cost-effectiveness of GoActive are expected to add substantially to the limited evidence on adolescent physical activity promotion. This study will include an objective, wrist-worn measure of physical activity, aligning with contemporary population surveillance studies[61,62,63] and ensuring greater protocol compliance for enhanced data retention and quality.[32,34,35] Achieving sustained health behaviour change is an established priority,[10] and so the inclusion of medium to long-term follow-up of participants will enable conclusions regarding the trajectories of change (in particular, whether any initial behaviour change is maintained). It will also form one of the largest cohorts in the field of adolescent physical activity promotion, providing many opportunities for secondary data analysis, in addition to testing causal pathways of effect and examining cost-effectiveness. Irrespective of study outcome, the evaluation of the GoActive intervention to increase physical activity in adolescents has the potential for significant academic impact.

For peer review only - http://bmjopen.bmj.com/site/about/guidelines.xhtml

Contributors: The PI, KC will have overall responsibility for project progress and direction; HB will be the day-to-day scientific lead for the project and FW will be the operational lead. EvS, PW and AV will advise on study procedures and evaluation from their respective disciplines; PW will additionally lead the design and evaluation of psychosocial outcomes. CC will lead the qualitative and mixed methods research. EW will lead the economic evaluation. Study sponsor and funders will have no role in the study design, collection, management, analysis, and interpretation of data, writing of the report; and the decision to submit the report for publication.

Funding

This project is funded by the National Institute for Health Research Public Health Research (13/90/18). Intervention delivery costs will be borne by Essex and Cambridgeshire County Councils.

Data sharing statement

After publication of trial analyses, and pending review of data access proposals by the investigators, data will be available on request from the corresponding author. The Principal Investigator, Co-Investigators, Statistician and Chair of the TSC will have access to the final trial dataset prior to conduct of the trial analyses. Statistical code for trial analysis will be available on request from the corresponding author.

There are no competing interests reported.

References

1 Janssen I, LeBlanc AG. Systematic review of the health benefits of physical activity and fitness in school-aged children and youth. Int. J. Behav. Nutr. Phys. Act. 2010;**7**:40.

2 Ekelund U, Luan J, Sherar LB, *et al.* Moderate to Vigorous Physical Activity and Sedentary Time and Cardiometabolic Risk Factors in Children and Adolescents. *JAMA J Am Med Assoc* 2012;**307**:704–12.

3 Dumith SC, Gigante DP, Domingues MR, *et al.* Physical activity change during adolescence: a systematic review and a pooled analysis. *Int J Epidemiol* 2011;**40**:685–98.

4 Raustorp A, Ekroth Y. Tracking of pedometer-determined physical activity: a 10-year follow-up study from adolescence to adulthood in Sweden. *J Phys Act Heal* 2013;**10**:1186–92.

5 Gill JMR, Cooper AR. Physical activity and prevention of type 2 diabetes mellitus. *Sports Med* 2008;**38**:807–24.

6 Khaw K-T, Wareham N, Bingham S, *et al.* Combined impact of health behaviours and mortality in men and women: the EPIC-Norfolk prospective population study. *PLoS Med* 2008;**5**:e12.

7 Corder K, Sharp SJ, Atkin AJ, *et al.* Change in objectively measured physical activity during the transition to adolescence. *Br J Sports Med* 2015;**49**:730–6.

8 Viner RM, Ozer EM, Denny S, *et al.* Adolescence and the social determinants of health. *Lancet* 2012;**379**:1641–52.

9 Department of Health. Annual Report of the Chief Medical Officer 2012: 'Our Children Deserve Better: Prevention Pays'. 2013. https://www.gov.uk/government/uploads/system/uploads/attachment_data/file/255237/2901304_CMO_complete_low_res_accessible.pdf

10 Gillis L, Tomkinson G, Olds T, *et al.* Research priorities for child and adolescent physical activity and sedentary behaviours: an international perspective using a twin-panel Delphi procedure. *Int J Behav Nutr Phys Act* 2013;**10**:112.

11 Report: Tackling Physical Inactivity— A Coordinated Approach | All-Party Commission on Physical

For peer review only - http://bmjopen.bmj.com/site/about/guidelines.xhtml

Activity on WordPress.com. https://activitycommission.com/report-tackling-physical-inactivity-a-coordinated-approach/ (accessed 21 Jul2016).

12 van Sluijs EMF, McMinn AM, Griffin SJ. Effectiveness of interventions to promote physical activity in children and adolescents: systematic review of controlled trials. *BMJ* 2007;**335**:703.

13 Kriemler S, Meyer U, Martin E, *et al.* Effect of school-based interventions on physical activity and fitness in children and adolescents: a review of reviews and systematic update. *Br J Sports Med* 2011;**45**:923–30.

14 Metcalf B, Henley W, Wilkin T. Effectiveness of intervention on physical activity of children: systematic review and meta-analysis of controlled trials with objectively measured outcomes (EarlyBird 54). *BMJ* 2012;**345**:e5888.

15 Dobbins M, DeCorby K, Robeson P, *et al.* School-based physical activity programs for promoting physical activity and fitness in children and adolescents aged 6-18. *Cochrane Database Syst Rev* Published Online First: 2009.http://www.scopus.com/inward/record.url?eid=2-s2.0-70249099867&partnerID=40&md5=1ad81652911cae49189a325a9acf1c25

16 Corder K, Atkin AJ, Ekelund U, *et al.* What do adolescents want in order to become more active? *BMC Public Health* 2013;**13**:718.

17 Jago R, Edwards MJ, Sebire SJ, *et al.* Bristol girls dance project (BGDP): Protocol for a cluster randomised controlled trial of an after-school dance programme to increase physical activity among 11--12 year old girls. BMC Public Health. 2013;:1003.

18 Smith J, Morgan P, Plotnikoff R, *et al.* Smart-phone obesity prevention trial for adolescent boys in low-income communities: The ATLAS RCT. *Pediatrics* 2014;**134**:e723-731.

19 Dobbins M, Husson H, DeCorby K, *et al.* School-based physical activity programs for promoting physical activity and fitness in children and adolescents aged 6 to 18. *Cochrane database Syst Rev* 2013;**2**:CD007651.

20 Lonsdale C, Rosenkranz RR, Peralta LR, *et al.* A systematic review and meta-analysis of interventions designed to increase moderate-to-vigorous physical activity in school physical education lessons. Prev. Med. (Baltim). 2013;**56**:152–61.

21 van Sluijs EMF, Kriemler S. Reflections on physical activity intervention research in young people - dos, don'ts, and critical thoughts. *Int J Behav Nutr Phys Act* 2016;**13**:25.

22 Corder K, Schiff A, Kesten JM, *et al.* Development of a universal approach to increase physical activity among adolescents: the GoActive intervention. *BMJ Open* 2015;**5**:e008610.

23 Chan AW, Tetzlaff JM, Altman DG, *et al.* SPIRIT 2013 statement: Defining standard protocol items for clinical trials. Ann. Intern. Med. 2013;**158**:200–7.

24 Chan AW, Tetzlaff JM, Altman DG, *et al.* SPIRIT 2013: New guidance for content of clinical trial protocols. Lancet. 2013;**381**:91–2.

25 Chan A-W, Tetzlaff JM, Gøtzsche PC, *et al.* SPIRIT 2013 explanation and elaboration: guidance for protocols of clinical trials. *BMJ* 2013;**346**:e7586.

26 Corder, Kirsten; Brown, Helen Elizabeth; Schiff, Anne;, van Sluijs EM. Feasibility study and pilot cluster randomised controlled trial of the GoActive Intervention aiming to promote physical activity among adolescents: outcomes and lessons learnt. *BMJ Open*

27 Shochet IM, Dadds MR, Holland D, *et al.* The efficacy of a universal school-based program to prevent adolescent depression. *J Clin Child Psychol* 2001;**30**:303–15.

28 Rowlands A V, Olds TS, Hillsdon M, *et al.* Assessing sedentary behavior with the GENEActiv: introducing the sedentary sphere. *Med Sci Sports Exerc* 2014;**46**:1235–47.

29 Phillips LRS, Parfitt G, Rowlands A V. Calibration of the GENEA accelerometer for assessment of physical activity intensity in children. *J Sci Med Sport* 2013;**16**:124–8.

30 Schaefer CA, Nigg CR, Hill JO, *et al.* Establishing and evaluating wrist cutpoints for the GENEActiv accelerometer in youth. *Med Sci Sports Exerc* 2014;**46**:826–33.

31 Rowlands A V., Yates T, Olds TS, *et al.* Sedentary sphere: Wrist-worn accelerometer-brand independent posture classification. *Med Sci Sports Exerc* 2016;**48**:748–54.

32 Rowlands A V., Rennie K, Kozarski R, *et al.* Children's physical activity assessed with wrist- and hip-worn accelerometers. *Med Sci Sports Exerc* 2014;**46**:2308–16.

33 Rowlands A V., Fraysse F, Catt M, *et al.* Comparability of measured acceleration from accelerometry-

For peer review only - http://bmjopen.bmj.com/site/about/guidelines.xhtml

based activity monitors. *Med Sci Sports Exerc* 2015;**47**:201–10.

34 Rosenberger ME, Buman MP, Haskell WL, *et al.* 24 Hours of Sleep, Sedentary Behavior, and Physical Activity with Nine Wearable Devices. *Med Sci Sports Exerc* 2015;:457–65.

35 Hildebrand M, Van Hees VT, Hansen BH, *et al.* Age group comparability of raw accelerometer output from wrist-and hip-worn monitors. *Med Sci Sports Exerc* 2014;**46**:1816–24.

36 Fairclough SJ, Noonan R, Rowlands A V., *et al.* Wear compliance and activity in children wearing wrist- and hip-mounted accelerometers. *Med Sci Sports Exerc* 2016;**48**:245–53.

37 Edwardson CLL, Rowlands A V., Bunnewell S, *et al.* Accuracy of Posture Allocation Algorithms for Thigh- and Waist-Worn Accelerometers. Med. Sci. Sports Exerc. 2016.

38 Mattocks C, Ness A, Leary S, *et al.* Use of accelerometers in a large field-based study of children: protocols, design issues, and effects on precision. *J Phys Act Health* 2008;**5 Suppl 1**:S98–111.

39 Riddoch CJ, Bo Andersen L, Wedderkopp N, *et al.* Physical activity levels and patterns of 9- and 15-yr-old European children. *Med Sci Sport Exerc* 2004;**36**:86–92.

40 Collings PJ, Wijndaele K, Corder K, *et al.* Levels and patterns of objectively-measured physical activity volume and intensity distribution in UK adolescents: the ROOTS study. *Int J Behav Nutr Phys Act* 2014;**11**:23.

41 Trost SG, Loprinzi PD, Moore R, *et al.* Comparison of accelerometer cut points for predicting activity intensity in youth. *Med Sci Sports Exerc* 2011;**43**:1360–8.

42 Van Sluijs EMF, Skidmore PML, Mwanza K, *et al.* Physical activity and dietary behaviour in a population-based sample of British 10-year old children: the SPEEDY study (Sport, Physical activity and Eating behaviour: environmental Determinants in Young people). *BMC Public Health* 2008;**8**:388.

43 Corder K, van Sluijs EMF, Ekelund U, *et al.* Changes in children's physical activity over 12 months: longitudinal results from the SPEEDY study. *Pediatrics* 2010;**126**:e926–35.

44 Corder K, van Sluijs EMF, Wright A, *et al.* Is it possible to assess free-living physical activity and energy expenditure in young people by self-report? *Am J Clin Nutr* 2009;**89**:862–70.

45 Saunders RP, Pate RR, Felton G, *et al.* Development of Questionnaires to Measure Psychosocial Influences on Children's Physical Activity. *Prev Med (Baltim)* 1997;**26**:241–7.

46 Ommundsen Y, Page A, Ku PW, *et al.* Cross-cultural, age and gender validation of a computerised questionnaire measuring personal, social and environmental associations with children's physical activity: The European Youth Heart Study. *Int J Behav Nutr Phys Act* 2008;**5**:29.http://www.ncbi.nlm.nih.gov/entrez/query.fcgi?cmd=Retrieve&db=PubMed&dopt=Citation&list_uids=18489736

47 Goodyer IM, Herbert J, Tamplin a, *et al.* Short-term outcome of major depression: II. Life events, family dysfunction, and friendship difficulties as predictors of persistent disorder. *J Am Acad Child Adolesc Psychiatry* 1997;**36**:474–80.

48 Clarke A, Friede T, Putz R, *et al.* Warwick-Edinburgh Mental Well-being Scale (WEMWBS): validated for teenage school students in England and Scotland. A mixed methods assessment. *BMC Public Health* 2011;**11**:487.

49 Rosenberg M. *Society and the adolescent self-image*. NJ: Princeton: : Princeton University Press 1965.

50 Eys M, Loughead T, Bray SR, *et al.* Development of a cohesion questionnaire for youth: the Youth Sport Environment Questionnaire. *J Sport Exerc Psychol* 2009;**31**:390–408.

51 Buss AH, Plomin R. *Temperament: Early developing personality traits*. 1984.

52 Saunders RP, Evans MH, Joshi P. Developing a Process-Evaluation Plan for Assessing Health Promotion Program Implementation: A How-To Guide. *Health Promot Pract* 2005;**6**:134–47.

53 Baranowski T, Stables G. Process Evaluations of the 5-a-Day Projects. *Heal Educ Behav* 2000;**27**:157–66.

54 Linnan L, Steckler A. Process Evaluation for Public Health Interventions and Research. *Eval Program Plann* 2004;**27**:118–9.

55 Stevens K. Assessing the performance of a new generic measure of health related quality of life for children and refining it for use in health state valuation. *Appl Heal Econ Heal Policy* 2011;**9**:157–69.

56 Stevens K. Valuation of the child health utility 9D index. *Pharmacoeconomics* 2012;**30**:729–47.

For peer review only - http://bmjopen.bmj.com/site/about/guidelines.xhtml

57 White IR, Thompson SG. Adjusting for partially missing baseline measurements in randomized trials. *Stat Med* 2005;**24**:993–1007.

58 MacKinnon DP, Fairchild AJ, Fritz MS. Mediation analysis. *Annu Rev Psychol* 2007;**58**:593–614.

59 Drummond MF, Stoddard GL, Torrance GW. Methods for the economic evaluation of health care programmes. Methods Econ. Eval. Heal. Care Program. 1988;:39–71.

60 ICMJE. Recommendations: Defining the Role of Authors and Contributors. http://www.icmje.org/recommendations/browse/roles-and-responsibilities/defining-the-role-of-authors-and-contributors.html (accessed 9 Aug2016).

61 Troiano R MCJ. Objective Measures of Physical Activity, Sleep, and Strength in U.S. National Health and Nutrition Examination Survey (NHANES) 2011-2014. *8th Intern Conf Diet Act Methods*

62 Mannini A, Intille SS, Rosenberger M, *et al.* Activity recognition using a single accelerometer placed at the wrist or ankle. *Med Sci Sports Exerc* 2013;**45**:2193–203.

63 Sudlow C, Gallacher J, Allen N, *et al.* UK biobank: an open access resource for identifying the causes of a wide range of complex diseases of middle and old age. *PLoS Med* 2015;**12**:e1001779.

For peer review only

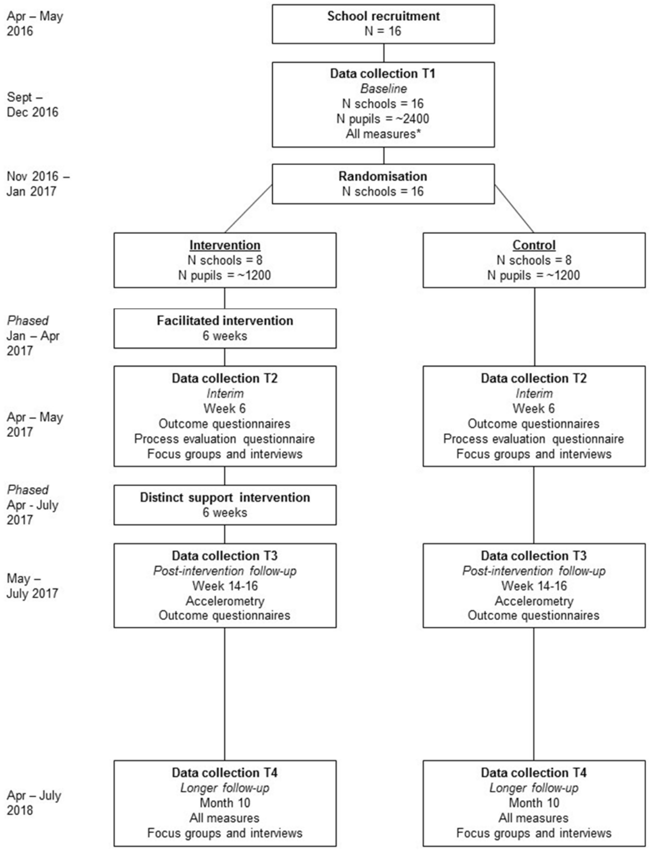

Figure 1: Measurement sessions included in the GoActive evaluation study.
*All measures includes accelerometry, anthropometry and outcomes questionnaire (student-reported physical activity participation, self-efficacy, peer support, group cohesion, self-esteem, friendship quality, and mood).

190x338mm (96 x 96 DPI)

Supplementary File 1: Process-Evaluation Plan for GoActive Intervention

Component	Process Evaluation Question	Data sources	Tools/Procedures
Fidelity	*To what extent was the intervention implemented consistently and as planned?*	- Students<br>- Mentors<br>- Facilitators<br>- Teachers<br>- GoActive staff	- T2 student website use: Google analytics on frequency and duration of website use, resources download and points upload statistics<br>- T2 mentor questionnaire<br>- T2 mentor log book<br>- T3 mentor focus groups<br>- T3 facilitator questionnaire*<br>- T3 facilitator focus groups/interviews*<br>- T2 facilitator log book<br>- T3 teacher questionnaire<br>- T3 teacher focus groups<br>- Field notes<br>- Interview notes<br>- Minutes of meetings<br>- Emails<br>- Logs (record keeping)<br>- Reflections<br>- Classroom observations*
Dose delivered	*To what extent were the units within the intervention implemented?*	- Students<br>- Mentors<br>- Facilitators<br>- Teachers<br>- GoActive staff	- T2 student questionnaire<br>- T2 student focus groups<br>- T3 student individual interviews<br>- T2 mentor questionnaire<br>- T3 mentor focus groups<br>- T2 mentor log book<br>- T3 facilitator questionnaire*<br>- T3 facilitator groups/interviews*<br>- T2 facilitator log book<br>- T3 teacher questionnaire<br>- T3 teacher focus groups<br>- Documentation of staff activities<br>- Review of notes and other documents<br>- Classroom observations*
Dose received	*Did students enjoy the GoActive activities?*<br>*Were mentors, teachers and facilitators satisfied with the intervention?*<br>*Were the GoActive staff satisfied with the intervention?*	- Students<br>- Mentors<br>- Facilitators	- T2 student website use: Google analytics on frequency and duration of website use, resources download and points upload statistics<br>- T2 student questionnaire<br>- T2 student focus groups<br>- T3 student individual interviews<br>- T2 mentor questionnaire<br>- T2 mentor log book<br>- T3 mentor focus groups<br>- T3 facilitator questionnaire*

For peer review only

		- Teachers<br>- Go Active staff	- T3 facilitator focus groups*<br>- T2 facilitator log book<br>- T3 teacher questionnaire<br>- T3 teacher focus groups<br>- Field notes<br>- Interview notes<br>- Minutes of meetings<br>- Emails<br>- Logs<br>- Reflections<br>- Classroom observations*
Reach	*Was the intervention delivered to at least 75% of Year 9 students?*	- Students	- T2 student questionnaire
Recruitment	*What procedures were followed to recruit schools and participants (students, teachers, mentors and facilitators) to the GoActive intervention?*	- GoActive staff<br>- Mentors<br>- Students	- £200 sporting equipment voucher for schools<br>- Sports clothing for mentors<br>Maintenance for students:<br>- Awards<br>- Prizes<br>- Competition
Context	*What were barriers and facilitators to implementing the GoActive intervention?*	- Students<br>- Mentors<br>- Facilitators<br>- Teachers<br>- GoActive staff	- T2 student questionnaire<br>- T2 student focus groups<br>- T3 student individual interview<br>- T2 mentor questionnaire<br>- T3 mentor focus groups<br>- T2 mentor log book<br>- T3 facilitator questionnaire*<br>- T3 facilitator focus groups*<br>- T2 facilitator log book<br>- T2 teacher questionnaire<br>- T3 teacher focus groups<br>- Field notes<br>- Interview notes<br>- Minutes of meetings<br>- Emails<br>- Logs (record keeping)<br>- Reflections<br>- Classroom observations*

*Ethics approval pending for these elements.

For peer review only

BMJ Open

Protocol for a cluster randomised controlled trial to evaluate the effectiveness and cost-effectiveness of the GoActive intervention to increase physical activity among 13-14 year-old adolescents

Journal:	*BMJ Open*
Manuscript ID	bmjopen-2016-014419.R3
Article Type:	Protocol
Date Submitted by the Author:	28-Mar-2017
Complete List of Authors:	Brown, Helen Elizabeth; University of Cambridge, MRC Epidemiology Unit and Centre for Diet and Activity Research<br>Whittle, Fiona; University of Cambridge, MRC Epidemiology Unit<br>Jong, Stephanie; University of Cambridge, MRC Epidemiology Unit and Centre for Diet and Activity Research<br>Croxson, Caroline; University of Oxford, Nuffield Department of Primary Care Health Sciences<br>Sharp, Stephen; University of Cambridge, MRC Epidemiology Unit<br>Wilkinson, Paul; University of Cambridge, Department of Psychiatry<br>Wilson, Edward; University of Cambridge, Cambridge Centre for Health Services Research<br>van Sluijs, Esther; University of Cambridge, MRC Epidemiology Unit and Centre for Diet and Activity Research<br>Vignoles, Anna; University of Cambridge, Faculty of Education<br>Corder, Kirsten; University of Cambridge, MRC Epidemiology Unit and Centre for Diet and Activity Research
**Primary Subject Heading**:	Public health
Secondary Subject Heading:	Epidemiology, Sports and exercise medicine
Keywords:	physical activity, promotion, intervention, adolescent, protocol, school

SCHOLARONE™
Manuscripts

Protocol for a cluster randomised controlled trial to evaluate the effectiveness and cost-effectiveness of the GoActive intervention to increase physical activity among 13-14 year-old adolescents

Helen Elizabeth Brown[1*], Fiona Whittle[1], Stephanie T. Jong[1], Caroline Croxson[2], Stephen J. Sharp[1], Paul Wilkinson[3], Edward CF Wilson[4], Esther MF van Sluijs[1], Anna Vignoles[5], Kirsten Corder[1]

[1]UKCRC Centre for Diet and Activity Research (CEDAR) *and* MRC Epidemiology Unit, University of Cambridge
[2]Nuffield Department of Primary Care Health Sciences, University of Oxford
[3]Department of Psychiatry, University of Cambridge
[4]Cambridge Centre for Health Services Research, University of Cambridge
[5]Faculty of Education, University of Cambridge
*Corresponding Author

Address for correspondence:
Dr Helen Elizabeth Brown
UK CRC Centre for Diet and Activity Research (CEDAR)
Department of MRC Epidemiology
University of Cambridge School of Clinical Medicine
Box 285 Institute of Metabolic Science
Cambridge Biomedical Campus
Cambridge
CB2 0QQ
Email: heb56@medschl.cam.ac.uk

Abstract

Introduction: Adolescent physical activity promotion is rarely effective, despite adolescence being critical for preventing physical activity decline. Low adolescent physical activity is likely to last into adulthood, increasing health risks. The GoActive intervention is evidence-based and was developed iteratively with adolescents and teachers. This intervention aims to increase physical activity through increased peer support, self-efficacy, group cohesion, self-esteem and friendship quality, and is implemented using a tiered-leadership system. We previously established feasibility in 1 school and conducted a pilot randomised controlled trial (RCT) in 3 schools.

Methods and analysis: We will conduct a school-based cluster RCT (CRCT) in 16 secondary schools targeting all Year 9 students (N=2400). In 8 schools, GoActive will run for 2 terms: weekly facilitation support from a council-funded health trainer will be offered in Term 1, with more distant support in Term 2. Tutor groups choose 2 weekly activities, encouraged by older adolescent mentors and weekly peer-leaders. Students gain points for trying new activities; points are entered into a between-class competition. Outcomes will be assessed at baseline, interim (week 6), post-intervention (week 14-16), and 10-month follow-up (main outcome). The primary outcome will be change from baseline in daily accelerometer-assessed moderate-to-

For peer review only - http://bmjopen.bmj.com/site/about/guidelines.xhtml

vigorous physical activity. Secondary outcomes include accelerometer-assessed activity intensities on weekdays/weekends; self-reported physical activity and psycho-social outcomes; cost-effectiveness and cost-utility analyses; mixed methods process evaluation integrating information from focus groups and participation logs/questionnaires.

Ethics and dissemination: Ethical approval for the conduct of the study was gained from the Cambridge Psychology Research Ethics Committee. Given the lack of rigorously evaluated interventions, and the inclusion of objective measurement of physical activity, long-term follow-up, and testing of causal pathways, the results of a CRCT of the effectiveness and cost-effectiveness of GoActive are expected to add substantially to the limited evidence on adolescent physical activity promotion. Workshops will be held with key stakeholders including students, parents, teachers, school governors, and government representatives, to discuss plans for wider dissemination of the intervention.

Strengths and limitations of this study

- The strengths of the GoActive evaluation study include the cluster-randomised controlled trial design, objective measurement of physical activity, long-term follow-up, and testing of causal pathways, to rigorously assess the effectiveness and cost-effectiveness of the GoActive programme.
- We will recruit 16 secondary schools from both Essex and Cambridgeshire. A possible limitation of the study is that, despite our purposive sampling of schools with varied socio-economic status, it is likely that participants may not be entirely representative of the wider UK population (particularly with regards to ethnicity).

Trial registration number: ISRCTN31583496

NB: The pilot trial of the GoActive intervention was registered retrospectively. We attempted to add the full CRCT prospectively, but were unfortunately not allowed to submit this as a new ISRCTN record and so it was added to the pilot record (which remained 'retrospective').

Originally registered: 18/2/2014

Funding reference: NIHR-PHR 13/90/18

Intervention delivery costs will be borne by Essex and Cambridgeshire County Councils.

Sponsor: University of Cambridge, contact**:** Mrs Carolyn Read, University of Cambridge School of Clinical Medicine, Box 111 Cambridge Biomedical Campus, Cambridge, CB2 0SP, United Kingdom, cad50@medschl.cam.ac.uk

Contact for Public and Scientific Queries: As address for correspondence

Public title: To establish the effect of the GoActive programme to increase physical activity among 13-14 year-old (Year 9) adolescents.

Scientific title: A cluster randomised controlled trial to evaluate the effectiveness and cost-effectiveness of the GoActive programme to increase physical activity among 13-14 year-old adolescents.

Protocol version: 5.0

Keywords: physical activity, promotion, intervention, adolescent, health behaviour

For peer review only - http://bmjopen.bmj.com/site/about/guidelines.xhtml

For peer review only

For peer review only - http://bmjopen.bmj.com/site/about/guidelines.xhtml

Background

Physical activity is protective against obesity and related metabolic disorders in young people[1,2]. Meta-analytic data from 20,871 4-18 year olds suggest that every 10-minute increase in moderate-to-vigorous activity (MVPA) is associated with a smaller waist circumference (-0.52 cm) and lower fasting insulin (-0.028 pmol/L).[2] In adolescence however, physical activity declines 7% per year.[3] Low physical activity in adolescence is also likely to progress to adulthood inactivity,[4] increasing the risk of diabetes, cancer and mortality.[5,6] Adolescence is therefore a critical period to increase physical activity,[7] both due to the aforementioned decline and because pubertal, brain and social development during this time leads to new capacity for changing health behaviours,[8] increasing the likelihood of long term change.

The 2012 Chief Medical Officer's report states the importance of physical activity among young people[9] and a recent international expert panel concluded that developing effective and sustainable interventions to increase physical activity among young people is the most important priority in the physical activity research field.[10] Further, the recently published report from the All-Party Commission on Physical Activity calls specifically for the creation of active schools, including the provision of a more diverse and inclusive offer of physical activity.[11]

Reviews highlight the limited efficacy of existing adolescent physical activity promotion interventions.[12,13,14,15] We have previously identified several possible reasons for this lack of effectiveness[16]; for example, many interventions only target subgroups (such as girls[17] or low socio-economic groups[18]) despite activity declining among all groups.[16] We aim to recruit the whole school year group for evaluation, and to target all groups in the GoActive intervention, which to our knowledge has rarely been done in physical activity promotion interventions. In addition, the decline in activity mainly occurs out of school;[16] however, many interventions only target specific school-based times; for example, school time[13,19] or Physical Education lessons.[20] whereas GoActive encourages participants to do more activity both in and out of school. Further, very few adolescent physical activity interventions, especially among older adolescents, have been evaluated using objective measurement of physical activity,[14] and including long-term follow-up, process evaluation, or an assessment of cost-effectiveness.[21] This therefore highlights an urgent need for more rigorous evaluation of potentially effective strategies to increase physical activity in adolescents.

Objectives

The primary aim of this study is to assess the 10-month effectiveness of the GoActive intervention to increase average daily objectively measured MVPA among 13-14 year-old adolescents. We will also assess the effect of GoActive immediately post-intervention, and on the following secondary outcomes: a) objectively assessed activity intensities during school time, weekday evenings and weekends; b) student-reported physical activity participation, self-efficacy, peer support, social networks, self-esteem, friendship quality (proposed mediators), and wellbeing, and school-level attendance and academic performance and c) body composition (body fat percentage and body mass index (BMI) z-score). We will investigate potential moderation of intervention effects by sex, socio-economic status, ethnicity, baseline activity level, and weight status, and potential mechanisms of effect by proposed mediators using a mixed-methods approach. Further, we will assess short term (within-trial) and potential long term cost-effectiveness of the GoActive

For peer review only - http://bmjopen.bmj.com/site/about/guidelines.xhtml

intervention, and will conduct a comprehensive process evaluation including questionnaires, focus groups (with participants, mentors, and teachers), individual interviews, data from intervention logs, and website analytics.

Intervention

The development of the "GoActive" (Get Others Active) intervention with supporting rationale has been described in detail previously.[22] Briefly, each Year 9 class (tutor group or home room class) chooses two activities each week from a selection provided. There are currently 20 activities available, utilising little or no equipment, and appealing to a wide variety of students (including Ultimate Frisbee, Zumba and Hula Hoop). Materials available on the password-protected GoActive intervention website include activity instructions (Quick Cards) which offer an overview of each activity, a short explanation, suggestions for adaptations, and provide advice, safety tips and 'factoids', in addition to a short video introducing each activity. GoActive is implemented using a tiered-leadership system where mentors (older adolescents within the school) and peer-leaders (within each Year 9 class) encourage students to try these activities each week. The mentors remain paired with each class for the duration of the intervention, whereas the peer-leaders (two per class each week, one male and one female) change every week. In addition to the student leaders, a local authority-funded intervention facilitator will support the programme during the first term of delivery and will provide distant support thereafter.

Teachers are encouraged to use one tutor time weekly to do one of the chosen activities as a class, however, students gain points for trying these new activities at any time in or out of school. Points are gained every time they try an activity; there is no expectation of time spent doing the activity as points are rewarded for the taking part itself. Individual students keep track of their own points privately on the study website and their points are entered into the between-class competition. Class rankings are available on the website to encourage teacher support and students receive small rewards (such as a Frisbee, a t-shirt, or a drawstring sports bag) for reaching individual points thresholds.

Methods

Study design

We will conduct a school-based cluster randomised controlled trial (CRCT) of the GoActive intervention. The study will be conducted in government-funded, non-fee-paying (state), all-ability, co-educational secondary schools including Year 9 students in Cambridgeshire and Essex, UK. After baseline measurements (September - December 2016), schools will be randomly allocated to one of two conditions; (1) to deliver the GoActive intervention to the whole of Year 9, or (2) to a no-treatment control group. Participant data collection will occur at baseline, 6 weeks, 14-16 weeks and 10 months (primary outcome). The protocol will be conducted and reported in accordance with SPIRIT guidance (Standard Protocol Items: Recommendations for Interventional Trials).[23,24,25] The trial has been registered with the ISRCTN registry (trial registration number: ISRCTN31583496).

Recruitment procedures

Schools

For peer review only - http://bmjopen.bmj.com/site/about/guidelines.xhtml

We will recruit 16 secondary schools with a mixture of socio-economic status, representative of UK variability. Head teachers, Year 9 leaders, and Physical Education (PE) leaders from all eligible schools will be sent an invitation letter and school information sheet via email. These documents will describe the study procedures (e.g. student recruitment and consent, measurements), and will include an electronic link to an information video describing GoActive. A follow-up phone call to each school will be made approximately one week after the initial invitation, asking for a meeting with relevant staff to discuss the study and request consent to participate. Phone calls and repeat emails will continue until 16 schools (8 in Cambridgeshire, and 8 in Essex) have provided consent to participate. We will also create a waiting list to replace any schools who may withdraw from the study prior to randomisation. We will also use our existing networks and school contacts to facilitate school recruitment. Schools who do not agree to take part will be asked to select the most relevant reason for their refusal from a pre-determined list (e.g. lack of interest, lack of time).

Participants

All Year 9 students (13-14 year-olds) in participating schools will be eligible to participate in study measurements. As in feasibility and pilot work, we plan to include disabled participants and those with learning or movement difficulties, taking care to follow advice from schools.[26] This is appropriate due to the inclusive nature of the GoActive intervention, and will help to avoid stigmatisation of any groups within schools.[27] As such, no exclusion criteria will be applied.

All Year 9 students and their parents will receive a paper invitation pack, including a participant information sheet and an invitation to participate in study measurements. These information packs will be distributed to students during an introductory assembly conducted by a member of the GoActive team; students will be asked to take the packs home to their parents. Parents will also be sent duplicate information via email (‘ParentMail’ or the appropriate equivalent system as agreed by the school). Parents will be asked to provide passive consent (active opt-out consent) for their child to take part in study measurements. We will give parents at least two weeks to respond (a final date for response will be included in all correspondence). After one week, parents will receive an additional copy to ensure further opportunity for opting out prior to study measurements. Parents will be given the option to phone or email the study team (in lieu of returning a form) to facilitate their ability to respond. Reminders will additionally be included in all relevant school media, including regular newsletters sent from the school. Written assent will be obtained from the students by research assistants trained in Good Clinical Practice prior to any baseline measurements taking place. Consent forms will be available on the study website www.goactive-uk.com after ethical approval for the trial has been obtained. Mentors and teachers will provide written consent or assent (for those older and younger than 16 years, respectively) to participate in process evaluation following the same procedures as study participants.

Parental opt-out responses ranged from 2 (<1%) to 18 (7%) in feasibility and pilot schools with 72-88% of eligible students assenting to participate.[26] Recruitment rates using this strategy are substantially higher than previous UK-based research in this age group using parental opt-in consent (23% of eligible participants).[7] Participants will be informed that they can discontinue all or any part of the study (either or both measurements and intervention) at any time at their or their parent/guardian’s request.

For peer review only - http://bmjopen.bmj.com/site/about/guidelines.xhtml

School randomisation

Schools will be stratified based on Pupil Premium (proxy for socio-economic status, below/above the county-specific median; for information: https://www.gov.uk/guidance/pupil-premium-information-for-schools-and-alternative-provision-settings), and county (i.e. Cambridgeshire or Essex). Randomisation lists for each stratum will be prepared by a statistician, using Stata (ref: StataCorp. 2015. Stata Statistical Software: Release 14. College Station, TX: StataCorp LP), after baseline measurements are completed to ensure schools and participants are unaware of their group allocation at baseline. Eight schools will be randomised to deliver the GoActive intervention and eight to a no-treatment control condition. For measurements after randomisation, it will not be possible to blind participants to randomised allocation, as the intervention schools will have received the GoActive intervention.

Measurement staff will be blinded to intervention condition throughout the study, as they will be trained and work separately from those involved in intervention delivery. Process evaluation with measurement staff will examine the success of blinding.

Control condition

The control group will receive no-treatment or ‘usual care’, and no intervention will be implemented. If we were to offer the control group the intervention after follow-up measures, it would prevent us from potentially assessing longer-term impact of the programme. As such, this study has no wait-list control condition.

Data collection

Measurements will be conducted at four time points by trained researchers (Figure 1). The primary measure of intervention effectiveness will be change from baseline in accelerometer-measured average daily MVPA at 10-month follow-up. All primary and secondary outcomes will be assessed at T1 and T4. Anthropometric measures will be removed from T3 (which will include all other outcomes, i.e. accelerometry and questionnaire-based measures), and T2 will focus on assessing the questionnaire-based measures only (including mediators of change). To prevent artificially inflated school-level clustering (due to weather conditions or school events) and facilitate recruitment and retention, measurements at each school will be staggered over ≥2 weeks using a predetermined schedule.

Figure 1 near here

Accelerometry

The primary outcome will be accelerometer-assessed change in average daily MVPA between baseline and 10-month follow-up. Secondary accelerometry outcomes will be change from baseline in average minutes spent in sedentary and light activity, as well as overall physical activity (counts per minute) during school, weekdays after school and at weekends.

Participants will be asked to wear a wrist-worn Axivity AX3 monitor at T1, T3 and T4. Participants will be asked to wear the monitors on a strap on their non-dominant wrist, continuously for seven consecutive days, (including when in water and when asleep). Wrist-worn monitors have been validated for use among children and adolescents, in laboratory and free-living environments, and to assess physical activity, sedentary time

For peer review only - http://bmjopen.bmj.com/site/about/guidelines.xhtml

and postural allocation.[28,29,30] There is evidence to support the increased acceptability and higher compliance rates of wrist-worn monitors compared to waist-worn monitors.[31,32,33,34,35,36,37] To further optimise accelerometer-wear compliance, we have developed a monitor wear and return protocol which is led by researchers (and not teachers), and includes regular reminders and an incentive (e.g. GoActive-branded headphones, GoActive branded pens). We have previously successfully applied this protocol in adolescent cohort studies to obtain high levels of valid accelerometry data (ROOTS: 825/930- 89%[7]; SPEEDY-3: 428/480 - 89%[16]).

Throughout data collection, we will continuously monitor response rates and take appropriate action (e.g. requesting teacher involvement) if it drops below 70% for the primary outcome. In cases where participants do not return their accelerometer after frequent requests, they may not be issued a monitor at subsequent measurements, but will be allowed to continue their participation in the study and all other (secondary) measures. This is to prevent excessive monitor loss. We deem this appropriate as sample size calculations indicate that we will retain 95% power should retention drop to 55% (80/150 participants predicted to participate in each school based on pilot data).

Once returned, data (continuous waveform data) from the accelerometers will be downloaded. Non-wear time with a minimum duration of 60 minutes will be removed; the acceleration threshold for identifying non-worn time will be based on visual inspection of the data...[38,39] As we will use a 24-hour protocol, we plan to apply a diurnal adjustment to reduce any bias that may occur if data was not fully representative of a 24 hour period but will also allow full use of the data collected.[40] For any daily analysis, we will set minimum criteria to ensure hours are equally distributed across whole day.[40]

Continuous waveform data will be converted to be comparable to cut-points used previously for ActiGraph accelerometers used to classify time spent sedentary (equivalent to ≤100 ActiGraph cpm), or in light (equivalent to 101 - 1999 ActiGraph cpm), moderate-vigorous (equivalent to ≥2000 ActiGraph cpm) or appropriate vector magnitude equivalents.[41,42,43] Monitor output will be reviewed prior to analysis to confirm that these decisions are appropriate for the population and monitor applied. Further, we will consult physical activity measurement experts to ensure we can be aware of relevant new methodology and apply where appropriate. Algorithms to identify sleep time are constantly in development. Given that we are operating a 24 hour wear time protocol, we will use the most up to date sleep identification algorithms to remove sleep time when estimating physical activity intensities (particularly sedentary time).

Anthropometry

Trained staff will measure height, weight and waist circumference following standardised operating procedures (e.g. wearing light clothing, removing shoes). Age- and sex-specific body fat percentage will be calculated from bio-electrical impedance (collected using Tanita TBF 300 scales), age- and sex-specific BMI z-score will be calculated from height and weight. Quality checking of researchers' anthropometry measurements will be conducted prior to baseline measurements and before 10-month follow-up.

Questionnaires

For peer review only - http://bmjopen.bmj.com/site/about/guidelines.xhtml

At each measurement session (i.e. T1, T2, T3, and T4), participants will complete a questionnaire concerning secondary outcomes, potential mediators or moderators, and items to monitor any adverse intervention effects. Physical activity type will be assessed using the 30-item Youth Physical Activity Questionnaire (YPAQ), which has previously been validated in 12-17 year olds.[44] Self-efficacy[45] and social support for physical activity[46] will be assessed using two scales (each with 3 items). Further items include friendship quality (8-item Cambridge Friendships Questionnaire),[47] well-being (14-item Edinburgh-Warwick Wellbeing Scale)[48] self-esteem (10- item Rosenberg Self Esteem Scale)[49], and an adapted social network modelling tool in which participants provided with a list of tutor group members and asked to select names of their friends)[50], and shyness and sociability (two 5-item measures from EAS temperament scale).[51] Questionnaires will be checked for completion before the end of the measurement sessions, and participants will be asked to complete any missing items. At T1, participants will respond to additional items providing demographic data (i.e. age, sex, ethnicity, language spoken at home, parent education, and family socio-economic status). School-level attendance and academic performance (from National Pupil Database) will be collected (publicly available data).

Process evaluation

Process evaluation will examine the proposed action model for the GoActive intervention (Supplementary File 1). These process evaluation questions emulate those depicted in Saunders, Evans and Joshi's (2005) process-evaluation plan to assess the implementation of a targeted health promotion intervention.[52] We focus on six components: fidelity, dose (delivered and received), reach, recruitment and context.[52,53,54] Supplementary File 1 demonstrates the applicability, and operationalisation of these components.

Intervention process data will include mixed-methods assessment of student, mentor, facilitator, teacher, and GoActive staff experiences, and perspectives on intervention delivery, feasibility, acceptance, and barriers/facilitators to participation. Reach (e.g. the intended amount of students that participate within the intervention) and dose received (e.g. the proportion of students who enter points on the GoActive website, how often students download QuickCards and view videos) will be established using the points entries on the study website, download statistics for intervention materials and mentor-reported participation. Process questionnaires will be administered at T2 and T3 for (both intervention and control) students, mentors, facilitators, and form teachers. Control participants will be asked to complete process questionnaires to determine possible contamination. We will include a GoActive logbook for the intervention facilitator and mentors to assess frequency of intervention delivery and any other descriptive notes at T2. Given the flexible, spontaneous and informal nature of the intervention (mentors/leaders attend the same school and can therefore encourage/motivate Year 9 students at any time during the week), observation of all intervention delivery is not feasible; but classroom observation* will be undertaken to complement other qualitative methods. Existing and emerging school practices which may affect students' physical activity behaviour will be documented and monitored in a structured manner using an adapted school environment questionnaire.[53]

A qualitative researcher will conduct semi-structured focus groups, using open-ended questions, after the facilitated intervention phase (T2) with Year 9 students in a sample of intervention schools. This sample will reflect variability of socio-economic status of the schools. Three Year 9 classrooms will be randomly selected

For peer review only - http://bmjopen.bmj.com/site/about/guidelines.xhtml

to participate in the focus groups. Approximately 12 students will be selected to participate in the focus groups from all eligible children within the 3 classrooms. Each focus group will be comprised of approximately four individuals in order to develop themes and generate adequate data. Students will be purposively sampled to ensure a mix of sex and ethnicity, and grouped by level of participation in the GoActive intervention and physical activity. Subsequent interviews with representatives from all other relevant groups within intervention schools (mentors, teachers, and facilitators) will commence in T3. Each focus group (separate for mentors, teachers and facilitators) will comprise of 3 to 8 individuals. An interview guide will be developed and updated as new issues and themes emerge; participants will be encouraged to discuss additional issues. Issues arising will inform the next round of questionnaires and subsequent focus groups, so that additional mechanisms of change can be investigated. In addition to focus groups, individual interviews will be conducted with a purposive sample of inactive and shy participants (identified using questionnaire data) at intervention schools to provide a deeper understanding of their intervention experience, and barriers and facilitators to participation (we anticipate these individuals will be more comfortable participating in one-to-one interviews).

At T4, additional semi-structured focus groups and interviews with students will explore maintenance of physical activity behaviour change, including who did or did not maintain physical activity behaviour change and why, whether GoActive helped and why or how, and other factors that helped or hindered physical activity maintenance. T2 participants will be re-invited, supplemented by additional students if needed. This gives us a unique opportunity to explore physical activity maintenance across time in the context of a trial, and to better understand barriers and facilitators to physical activity maintenance.

Cost-effectiveness

We will conduct both a within-trial and decision-model based economic evaluation. The within-trial analysis will be from the cost perspective of the school/local authority. Cost data collected will include intervention-related facilitator time, travel, and expenses collected by schools/researchers. Outcomes will comprise change in MVPA and quality adjusted life years (QALYs) gained. These will be assessed using the CHU-9D[55] and converted to health state utilities using UK specific valuations.[56] Change in physical activity observed and costs to schools/local authorities will be input into a previously developed model to predict longer term costs (to the National Health Service (NHS)) and QALYS hence cost-effectiveness from a public sector perspective (defined as local authority and NHS).

Data collection forms and questionnaires for all measurements are available on request from the corresponding author.

Data management and monitoring

All data will be collected and managed in line with International Conference on Harmonisation Good Clinical Practice guidelines. Real time entry and retrospective data validation checks will be conducted. All paper based questionnaire data will be professionally double data entered and a sample verified for accuracy. Data will be stored securely at the MRC Epidemiology Unit, Cambridge, UK. The MRC Epidemiology Unit specialist teams will provide support for training, and quality assessment and control of measurements, and

For peer review only - http://bmjopen.bmj.com/site/about/guidelines.xhtml

this support will ensure that collection, processing, protection and management of data is timely and of high quality. We will ensure that all provided data are treated as confidential and stored securely. Where this is electronic, data are held on secure computer systems with at minimum password access. All identifiable data will be held on a separate computer system with access limited to appropriate staff by group and password permissions. Personal data will be stored and accessed up to 20 years after study completion.

Due to the low risk nature of the trial, a formal data monitoring committee has not been appointed. However, the Trial Steering Committee (TSC) will receive regular reports from the investigators and will monitor trial progress and conduct. The TSC will consist of an independent chair, one independent expert, two lay representatives (including a representative from educational sector) and at least two investigators; the committee will be at least 75% independent. The study coordinator and a sponsor representative will be invited as observers. The TSC will meet approximately once per year, or more frequently if needed. The TSC is responsible for communicating any issues of concern to the Sponsor, specifically where the integrity of the study or data or patient safety could be comprised. The study coordinator will also monitor trial conduct and will report independently to the MRC Epidemiology Unit Clinical Research Manager. Potential harms will be monitored by the study team. These will be reviewed by the Study Coordinator, Principal Investigator, and Trial Steering Committee, and will include reported adverse events (e.g. injuries or psychological indicators such as well-being). While we do not expect harm as a result of the GoActive intervention or this trial, it is insured by the University of Cambridge who would provide compensation in case of harm.

The council-funded intervention facilitators will work closely with mentors and research staff to monitor protocol adherence. Poor adherence will be discussed with the research team and TSC, and strategies will be put in place where necessary. No activities are prohibited during the trial as students are expected to do their normal physical activities, including school PE.

Any protocol amendments will be proposed to the TSC and subsequently altered if necessary before submission to funder (NIHR) for approval. Protocol updates will then be uploaded to the NIHR website and trial registry if relevant.

Analyses

Sample size

We aim to detect a 5-minute difference in change in MVPA per day at 10-month follow-up, as observed in the pilot study.[26] A 5-minute increase is relevant at population level, as it would increase the proportion of adolescents meeting the guidelines of 60 minutes of MVPA per day from 43% to 50% (based on baseline pilot data), with significant impact on population health.[2] To estimate the required sample size, the following parameters have been used: power=85%, significance level=5%, standard deviation=17.8 (observed in the GoActive pilot),[26] intraclass correlation coefficient=0.034 (observed in SPEEDY-3, N=57 schools),[43] correlation between baseline and follow-up MVPA=0.59 (observed in GoActive pilot, to account for adjustment for baseline MVPA),[26] and average cluster size=100. Based on these parameters, we estimate N=1310 participants will be required for the primary effectiveness analysis. To account for potential school dropout and an estimated loss-to-follow-up of 30-40%, we aim to recruit 16 schools with 150 participants

For peer review only - http://bmjopen.bmj.com/site/about/guidelines.xhtml

(total N=2400; average recruitment per school in pilot=154).[26] Should a school have more than 150 students in Year 9, we will include all those who assent to measurement.

Quantitative analyses

The primary analysis of effectiveness, intermediate, and safety outcomes will use an Intention To Treat (ITT) population, which includes all participants in the group to which they were randomised, regardless of the intervention received. A secondary analysis of efficacy and intermediate outcomes will use a Per Protocol (PP) population. Inclusion in the PP population will be based on the degree of usage of the intervention website and/or submission of points, and will be defined once clean data are available (but before the start of any trial analyses), when the distributions of degree of website usage can be inspected.

Outcome analyses

The primary efficacy outcome, MVPA, will be compared between intervention and control groups using analysis of covariance (ANCOVA), with adjustment for baseline MVPA; robust standard errors will be calculated to allow for the non-independence of individuals within each school. Where baseline values of MVPA are missing, the missing indicator method will be used to enable these participants to be included in the analysis.[57] An estimate of the intervention effect, 95% confidence interval, and p-value will be calculated. A similar method will be used for the secondary efficacy outcomes. School-level data will also enable analysis of key differences between those participating in the evaluation, and the wider school population; for example, patterns of non-response by demographic variables will be explored. Subgroup analyses by pre-specified moderators (sex, socio-economic status, ethnicity, baseline activity level, weight status) will be performed for the primary outcome only. The interaction between randomised group and each moderator will be tested, and if the p-value is <0.05, the intervention effect (difference between intervention and control, and 95% confidence interval) will be estimated within each subgroup. The effect on potential mediating variables will initially be assessed as described above. We will subsequently conduct formal mediation analyses using the product of coefficient method[58] to assess the underlying causal pathways of the intervention.

Qualitative analyses

Focus groups and interviews will be audio recorded, transcribed verbatim and made anonymous. Data will be analysed using thematic analysis following a six phase model[59], facilitated by QSR NVivo. Coding will be inductive, incorporating emerging themes as well as topics presented a priori in the interview guide. Initial analyses will inform future data collection and analysis. Interim themes will be discussed by the research team to reach consensus.

Cost-effectiveness analyses

Cost-effectiveness analyses will follow standardised protocols.[60] The main economic outcome will be the incremental cost-effectiveness ratio, expressed as incremental costs per incremental change in physical activity (MVPA) and per QALY gained (based on CHU-9D) for the trial period (including follow-up). Data collected will include intervention time, travel, expenses, resource use, and study-specific costs. In addition, if GoActive increases physical activity, this should reduce adult chronic disease via changes in weight or BMI, and blood glucose. To establish whether GoActive could increase length and/or quality of life and at

For peer review only - http://bmjopen.bmj.com/site/about/guidelines.xhtml

what cost, it is not practical to conduct lifetime follow-up, therefore we propose adjusting an existing decision-analytic model to estimate the impact of physical activity on disease risk, quality-adjusted life expectancy (QALY) and cost to the NHS. The modelled analysis will therefore be from a public sector perspective (schools/local authority and NHS).

Further analyses

Further research questions can be addressed using the cohort data, including (but not limited to) assessment of the predictors of activity maintenance, and the longitudinal association between physical activity/sedentary behaviour and a) academic performance; b) shyness and sociability; and c) friendship quality. All proposed analyses will be approved by the project group, and authorship of manuscripts will be informed by recommended guidelines.[61]

Ethics and dissemination

Ethical approval for the conduct of the study was gained from the Cambridge Psychology Research Ethics Committee, who previously provided ethical approval for the development, feasibility and pilot studies following similar procedures. [22,26]

If successful, it would be appropriate to disseminate this programme to schools and councils across the UK (in addition to peer-reviewed publications). Towards the end of the project, a deliberative dialogue workshop will be held with key stakeholders including students, parents, teachers, school governors, and representatives from local/national government. This final workshop will focus on plans for dissemination of results, and will include discussion of the process of programme adaptation to a diverse range of secondary schools and further ways of ensuring long-term appeal for adolescents. We anticipate that dissemination could be facilitated through the study website, hosting intervention materials (including videos) and study information.

Given the lack of rigorously evaluated interventions, the results of a CRCT of the effectiveness and cost-effectiveness of GoActive are expected to add substantially to the limited evidence on adolescent physical activity promotion. This study will include an objective, wrist-worn measure of physical activity, aligning with contemporary population surveillance studies[62,63,64] and ensuring greater protocol compliance for enhanced data retention and quality.[32,34,35] Achieving sustained health behaviour change is an established priority,[10] and so the inclusion of medium to long-term follow-up of participants will enable conclusions regarding the trajectories of change (in particular, whether any initial behaviour change is maintained). It will also form one of the largest cohorts in the field of adolescent physical activity promotion, providing many opportunities for secondary data analysis, in addition to testing causal pathways of effect and examining cost-effectiveness. Irrespective of study outcome, the evaluation of the GoActive intervention to increase physical activity in adolescents has the potential for significant academic impact.

Contributors: The PI, KC will have overall responsibility for project progress and direction; HB will be the day-to-day scientific lead for the project and FW will be the operational lead. EvS, PW and AV will advise on study procedures and evaluation from their respective disciplines; PW will additionally lead the design and evaluation of psychosocial outcomes. CC will lead the qualitative and mixed methods research. EW will lead

For peer review only - http://bmjopen.bmj.com/site/about/guidelines.xhtml

the economic evaluation. Study sponsor and funders will have no role in the study design, collection, management, analysis, and interpretation of data, writing of the report; and the decision to submit the report for publication.

Funding

This project is funded by the National Institute for Health Research Public Health Research (13/90/18). Intervention delivery costs will be borne by Essex and Cambridgeshire County Councils.

Data sharing statement

After publication of trial analyses, and pending review of data access proposals by the investigators, data will be available on request from the corresponding author. The Principal Investigator, Co-Investigators, Statistician and Chair of the TSC will have access to the final trial dataset prior to conduct of the trial analyses. Statistical code for trial analysis will be available on request from the corresponding author.

There are no competing interests reported.

References

1 Janssen I, LeBlanc AG. Systematic review of the health benefits of physical activity and fitness in school-aged children and youth. Int. J. Behav. Nutr. Phys. Act. 2010;**7**:40.

2 Ekelund U, Luan J, Sherar LB, *et al.* Moderate to Vigorous Physical Activity and Sedentary Time and Cardiometabolic Risk Factors in Children and Adolescents. *JAMA J Am Med Assoc* 2012;**307**:704–12.

3 Dumith SC, Gigante DP, Domingues MR, *et al.* Physical activity change during adolescence: a systematic review and a pooled analysis. *Int J Epidemiol* 2011;**40**:685–98.

4 Raustorp A, Ekroth Y. Tracking of pedometer-determined physical activity: a 10-year follow-up study from adolescence to adulthood in Sweden. *J Phys Act Heal* 2013;**10**:1186–92.

5 Gill JMR, Cooper AR. Physical activity and prevention of type 2 diabetes mellitus. *Sports Med* 2008;**38**:807–24.

6 Khaw K-T, Wareham N, Bingham S, *et al.* Combined impact of health behaviours and mortality in men and women: the EPIC-Norfolk prospective population study. *PLoS Med* 2008;**5**:e12.

7 Corder K, Sharp SJ, Atkin AJ, *et al.* Change in objectively measured physical activity during the transition to adolescence. *Br J Sports Med* 2015;**49**:730–6.

8 Viner RM, Ozer EM, Denny S, *et al.* Adolescence and the social determinants of health. *Lancet* 2012;**379**:1641–52.

9 Department of Health. Annual Report of the Chief Medical Officer 2012: ‘Our Children Deserve Better: Prevention Pays’. 2013. https://www.gov.uk/government/uploads/system/uploads/attachment_data/file/255237/2901304_CMO_complete_low_res_accessible.pdf

10 Gillis L, Tomkinson G, Olds T, *et al.* Research priorities for child and adolescent physical activity and sedentary behaviours: an international perspective using a twin-panel Delphi procedure. *Int J Behav Nutr Phys Act* 2013;**10**:112.

11 Report: Tackling Physical Inactivity— A Coordinated Approach | All-Party Commission on Physical Activity on WordPress.com. https://activitycommission.com/report-tackling-physical-inactivity-a-coordinated-approach/ (accessed 21 Jul2016).

12 van Sluijs EMF, McMinn AM, Griffin SJ. Effectiveness of interventions to promote physical activity in children and adolescents: systematic review of controlled trials. *BMJ* 2007;**335**:703.

13 Kriemler S, Meyer U, Martin E, *et al.* Effect of school-based interventions on physical activity and fitness in children and adolescents: a review of reviews and systematic update. *Br J Sports Med*

For peer review only - http://bmjopen.bmj.com/site/about/guidelines.xhtml

2011;**45**:923–30.

14 Metcalf B, Henley W, Wilkin T. Effectiveness of intervention on physical activity of children: systematic review and meta-analysis of controlled trials with objectively measured outcomes (EarlyBird 54). *BMJ* 2012;**345**:e5888.

15 Dobbins M, DeCorby K, Robeson P, *et al.* School-based physical activity programs for promoting physical activity and fitness in children and adolescents aged 6-18. *Cochrane Database Syst Rev* Published Online First: 2009.http://www.scopus.com/inward/record.url?eid=2-s2.0-70249099867&partnerID=40&md5=1ad81652911cae49189a325a9acf1c25

16 Corder K, Atkin AJ, Ekelund U, *et al.* What do adolescents want in order to become more active? *BMC Public Health* 2013;**13**:718.

17 Jago R, Edwards MJ, Sebire SJ, *et al.* Bristol girls dance project (BGDP): Protocol for a cluster randomised controlled trial of an after-school dance programme to increase physical activity among 11--12 year old girls. BMC Public Health. 2013;:1003.

18 Smith J, Morgan P, Plotnikoff R, *et al.* Smart-phone obesity prevention trial for adolescent boys in low-income communities: The ATLAS RCT. *Pediatrics* 2014;**134**:e723-731.

19 Dobbins M, Husson H, DeCorby K, *et al.* School-based physical activity programs for promoting physical activity and fitness in children and adolescents aged 6 to 18. *Cochrane database Syst Rev* 2013;**2**:CD007651.

20 Lonsdale C, Rosenkranz RR, Peralta LR, *et al.* A systematic review and meta-analysis of interventions designed to increase moderate-to-vigorous physical activity in school physical education lessons. Prev. Med. (Baltim). 2013;**56**:152–61.

21 van Sluijs EMF, Kriemler S. Reflections on physical activity intervention research in young people - dos, don’ts, and critical thoughts. *Int J Behav Nutr Phys Act* 2016;**13**:25.

22 Corder K, Schiff A, Kesten JM, *et al.* Development of a universal approach to increase physical activity among adolescents: the GoActive intervention. *BMJ Open* 2015;**5**:e008610.

23 Chan AW, Tetzlaff JM, Altman DG, *et al.* SPIRIT 2013 statement: Defining standard protocol items for clinical trials. Ann. Intern. Med. 2013;**158**:200–7.

24 Chan AW, Tetzlaff JM, Altman DG, *et al.* SPIRIT 2013: New guidance for content of clinical trial protocols. Lancet. 2013;**381**:91–2.

25 Chan A-W, Tetzlaff JM, Gøtzsche PC, *et al.* SPIRIT 2013 explanation and elaboration: guidance for protocols of clinical trials. *BMJ* 2013;**346**:e7586.

26 Corder, Kirsten; Brown, Helen Elizabeth; Schiff, Anne;, van Sluijs EM. Feasibility study and pilot cluster randomised controlled trial of the GoActive Intervention aiming to promote physical activity among adolescents: outcomes and lessons learnt. *BMJ Open*

27 Shochet IM, Dadds MR, Holland D, *et al.* The efficacy of a universal school-based program to prevent adolescent depression. *J Clin Child Psychol* 2001;**30**:303–15.

28 Rowlands A V, Olds TS, Hillsdon M, *et al.* Assessing sedentary behavior with the GENEActiv: introducing the sedentary sphere. *Med Sci Sports Exerc* 2014;**46**:1235–47.

29 Phillips LRS, Parfitt G, Rowlands A V. Calibration of the GENEA accelerometer for assessment of physical activity intensity in children. *J Sci Med Sport* 2013;**16**:124–8.

30 Schaefer CA, Nigg CR, Hill JO, *et al.* Establishing and evaluating wrist cutpoints for the GENEActiv accelerometer in youth. *Med Sci Sports Exerc* 2014;**46**:826–33.

31 Rowlands A V., Yates T, Olds TS, *et al.* Sedentary sphere: Wrist-worn accelerometer-brand independent posture classification. *Med Sci Sports Exerc* 2016;**48**:748–54.

32 Rowlands A V., Rennie K, Kozarski R, *et al.* Children’s physical activity assessed with wrist- and hip-worn accelerometers. *Med Sci Sports Exerc* 2014;**46**:2308–16.

33 Rowlands A V., Fraysse F, Catt M, *et al.* Comparability of measured acceleration from accelerometry-based activity monitors. *Med Sci Sports Exerc* 2015;**47**:201–10.

34 Rosenberger ME, Buman MP, Haskell WL, *et al.* 24 Hours of Sleep, Sedentary Behavior, and Physical Activity with Nine Wearable Devices. *Med Sci Sports Exerc* 2015;:457–65.

35 Hildebrand M, Van Hees VT, Hansen BH, *et al.* Age group comparability of raw accelerometer output from wrist-and hip-worn monitors. *Med Sci Sports Exerc* 2014;**46**:1816–24.

36 Fairclough SJ, Noonan R, Rowlands A V., *et al.* Wear compliance and activity in children wearing

For peer review only - http://bmjopen.bmj.com/site/about/guidelines.xhtml

wrist- and hip-mounted accelerometers. *Med Sci Sports Exerc* 2016;**48**:245–53.

37 Edwardson CLL, Rowlands A V., Bunnewell S, *et al.* Accuracy of Posture Allocation Algorithms for Thigh- and Waist-Worn Accelerometers. Med. Sci. Sports Exerc. 2016.

38 Mattocks C, Ness A, Leary S, *et al.* Use of accelerometers in a large field-based study of children: protocols, design issues, and effects on precision. *J Phys Act Health* 2008;**5 Suppl 1**:S98–111.

39 Riddoch CJ, Bo Andersen L, Wedderkopp N, *et al.* Physical activity levels and patterns of 9- and 15-yr-old European children. *Med Sci Sport Exerc* 2004;**36**:86–92.

40 Collings PJ, Wijndaele K, Corder K, *et al.* Levels and patterns of objectively-measured physical activity volume and intensity distribution in UK adolescents: the ROOTS study. *Int J Behav Nutr Phys Act* 2014;**11**:23.

41 Trost SG, Loprinzi PD, Moore R, *et al.* Comparison of accelerometer cut points for predicting activity intensity in youth. *Med Sci Sports Exerc* 2011;**43**:1360–8.

42 Van Sluijs EMF, Skidmore PML, Mwanza K, *et al.* Physical activity and dietary behaviour in a population-based sample of British 10-year old children: the SPEEDY study (Sport, Physical activity and Eating behaviour: environmental Determinants in Young people). *BMC Public Health* 2008;**8**:388.

43 Corder K, van Sluijs EMF, Ekelund U, *et al.* Changes in children's physical activity over 12 months: longitudinal results from the SPEEDY study. *Pediatrics* 2010;**126**:e926–35.

44 Corder K, van Sluijs EMF, Wright A, *et al.* Is it possible to assess free-living physical activity and energy expenditure in young people by self-report? *Am J Clin Nutr* 2009;**89**:862–70.

45 Saunders RP, Pate RR, Felton G, *et al.* Development of Questionnaires to Measure Psychosocial Influences on Children's Physical Activity. *Prev Med (Baltim)* 1997;**26**:241–7.

46 Ommundsen Y, Page A, Ku PW, *et al.* Cross-cultural, age and gender validation of a computerised questionnaire measuring personal, social and environmental associations with children's physical activity: The European Youth Heart Study. *Int J Behav Nutr Phys Act* 2008;**5**:29.http://www.ncbi.nlm.nih.gov/entrez/query.fcgi?cmd=Retrieve&db=PubMed&dopt=Citation&list_uids=18489736

47 Goodyer IM, Herbert J, Tamplin a, *et al.* Short-term outcome of major depression: II. Life events, family dysfunction, and friendship difficulties as predictors of persistent disorder. *J Am Acad Child Adolesc Psychiatry* 1997;**36**:474–80.

48 Clarke A, Friede T, Putz R, *et al.* Warwick-Edinburgh Mental Well-being Scale (WEMWBS): validated for teenage school students in England and Scotland. A mixed methods assessment. *BMC Public Health* 2011;**11**:487.

49 Rosenberg M. *Society and the adolescent self-image*. NJ: Princeton: : Princeton University Press 1965.

50 Eys M, Loughead T, Bray SR, *et al.* Development of a cohesion questionnaire for youth: the Youth Sport Environment Questionnaire. *J Sport Exerc Psychol* 2009;**31**:390–408.

51 Buss AH, Plomin R. *Temperament: Early developing personality traits*. 1984.

52 Saunders RP, Evans MH, Joshi P. Developing a Process-Evaluation Plan for Assessing Health Promotion Program Implementation: A How-To Guide. *Health Promot Pract* 2005;**6**:134–47.

53 Baranowski T, Stables G. Process Evaluations of the 5-a-Day Projects. *Heal Educ Behav* 2000;**27**:157–66.

54 Linnan L, Steckler A. Process Evaluation for Public Health Interventions and Research. *Eval Program Plann* 2004;**27**:118–9.

55 Stevens K. Assessing the performance of a new generic measure of health related quality of life for children and refining it for use in health state valuation. *Appl Heal Econ Heal Policy* 2011;**9**:157–69.

56 Stevens K. Valuation of the child health utility 9D index. *Pharmacoeconomics* 2012;**30**:729–47.

57 White IR, Thompson SG. Adjusting for partially missing baseline measurements in randomized trials. *Stat Med* 2005;**24**:993–1007.

58 MacKinnon DP, Fairchild AJ, Fritz MS. Mediation analysis. *Annu Rev Psychol* 2007;**58**:593–614.

59 Braun V, Clarke V. Using thematic analysis in psychology. *Qual Res Psychol* 2006;**3**:77–101.

60 Drummond MF, Stoddard GL, Torrance GW. Methods for the economic evaluation of health care programmes. Methods Econ. Eval. Heal. Care Program. 1988;:39–71.

For peer review only - http://bmjopen.bmj.com/site/about/guidelines.xhtml

61 ICMJE. Recommendations: Defining the Role of Authors and Contributors. http://www.icmje.org/recommendations/browse/roles-and-responsibilities/defining-the-role-of-authors-and-contributors.html (accessed 9 Aug2016).

62 Troiano R MCJ. Objective Measures of Physical Activity, Sleep, and Strength in U.S. National Health and Nutrition Examination Survey (NHANES) 2011-2014. *8th Intern Conf Diet Act Methods*

63 Mannini A, Intille SS, Rosenberger M, *et al.* Activity recognition using a single accelerometer placed at the wrist or ankle. *Med Sci Sports Exerc* 2013;**45**:2193–203.

64 Sudlow C, Gallacher J, Allen N, *et al.* UK biobank: an open access resource for identifying the causes of a wide range of complex diseases of middle and old age. *PLoS Med* 2015;**12**:e1001779.

For peer review only

Figure 1: Measurement sessions included in the GoActive evaluation

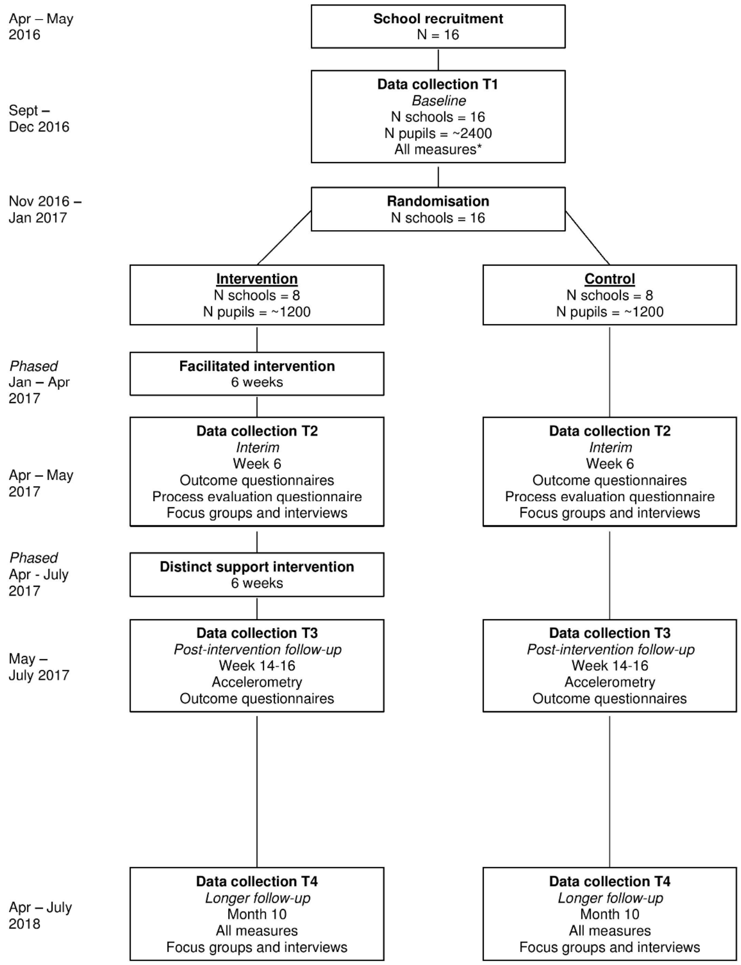

*All measures includes accelerometry, anthropometry and outcomes questionnaire (student-reported physical activity participation, self-efficacy, peer support, group cohesion, self-esteem, friendship quality, and mood).

Figure 1: Measurement sessions included in the GoActive evaluation

*All measures includes accelerometry, anthropometry and outcomes questionnaire (student-reported physical activity participation, self-efficacy, peer support, group cohesion, self-esteem, friendship quality, and mood).

210x297mm (300 x 300 DPI)

Supplementary File 1: Process-Evaluation Plan for GoActive Intervention

Component	Process Evaluation Question	Data sources	Tools/Procedures
Fidelity	*To what extent was the intervention implemented consistently and as planned?*	- Students<br>- Mentors<br>- Facilitators<br>- Teachers<br>- GoActive staff	Fidelity of implementation will be assessed utilizing an observation procedure*. This will include observing the encouragement and modelling of activities from mentors and leaders to students<br>- T2 student website use: Google analytics on points uploaded, and hence, prizes redeemed<br>- T2 mentor questionnaire<br>- T2 mentor log book<br>- T3 mentor focus groups<br>- T3 facilitator questionnaire*<br>- T3 facilitator focus groups/interviews*<br>- T2 facilitator log book<br>- T3 teacher questionnaire<br>- T3 teacher focus groups<br>- Field notes<br>- Interview notes<br>- Minutes of meetings<br>- Emails<br>- Logs (record keeping)<br>- Reflections
Dose delivered	*To what extent were the units within the intervention implemented?*	- Students<br>- Mentors<br>- Facilitators<br>- Teachers<br>- GoActive staff	- T2 student questionnaire<br>- T2 student focus groups<br>- T3 student individual interviews<br>- T2 mentor questionnaire<br>- T3 mentor focus groups<br>- T2 mentor log book<br>- T3 facilitator questionnaire*<br>- T3 facilitator groups/interviews*<br>- T2 facilitator log book<br>- T3 teacher questionnaire<br>- T3 teacher focus groups<br>- Documentation of staff activities<br>- Review of notes and other documents<br>- Classroom observations*
Dose received	*Did students enjoy the GoActive activities?*<br>*Were mentors, teachers and facilitators satisfied with the intervention?*<br>*Were the GoActive staff satisfied with the intervention?*	- Students<br>- Mentors	- T2 student website use: Google analytics on frequency and duration of website use, resources download and points upload statistics<br>- T2 student questionnaire<br>- T2 student focus groups<br>- T3 student individual interviews<br>- T2 mentor questionnaire<br>- T2 mentor log book

For peer review only

		- Facilitators<br>- Teachers<br>- Go Active staff	- T3 mentor focus groups<br>- T3 facilitator questionnaire*<br>- T3 facilitator focus groups*<br>- T2 facilitator log book<br>- T3 teacher questionnaire<br>- T3 teacher focus groups<br>- Field notes<br>- Interview notes<br>- Minutes of meetings<br>- Emails<br>- Logs<br>- Reflections<br>- Classroom observations*
Reach	*Was the intervention delivered to at least 75% of Year 9 students?*	- Students	- T2 student questionnaire
Recruitment	*What procedures were followed to recruit schools and participants (students, teachers, mentors and facilitators) to the GoActive intervention?*	- GoActive staff<br>- Mentors<br>- Students	- £200 sporting equipment voucher for schools<br>- Sports clothing for mentors<br>Maintenance for students:<br>- Awards<br>- Prizes<br>- Competition
Context	*What were barriers and facilitators to implementing the GoActive intervention?*	- Students<br>- Mentors<br>- Facilitators<br>- Teachers<br>- GoActive staff	- T2 student questionnaire<br>- T2 student focus groups<br>- T3 student individual interview<br>- T2 mentor questionnaire<br>- T3 mentor focus groups<br>- T2 mentor log book<br>- T3 facilitator questionnaire*<br>- T3 facilitator focus groups*<br>- T2 facilitator log book<br>- T2 teacher questionnaire<br>- T3 teacher focus groups<br>- Field notes<br>- Interview notes<br>- Minutes of meetings<br>- Emails<br>- Logs (record keeping)<br>- Reflections<br>- Classroom observations*

*Ethics approval pending for these elements.

For peer review only

SPIRIT

STANDARD PROTOCOL ITEMS: RECOMMENDATIONS FOR INTERVENTIONAL TRIALS

SPIRIT 2013 Checklist: Recommended items to address in a clinical trial protocol and related documents*

Section/item	Item No	Description	Addressed on page number
**Administrative information**			
Title	1	Descriptive title identifying the study design, population, interventions, and, if applicable, trial acronym	1
Trial registration	2a	Trial identifier and registry name. If not yet registered, name of intended registry	2
	2b	All items from the World Health Organization Trial Registration Data Set	1-10
Protocol version	3	Date and version identifier	2
Funding	4	Sources and types of financial, material, and other support	2
Roles and responsibilities	5a	Names, affiliations, and roles of protocol contributors	1
	5b	Name and contact information for the trial sponsor	2
	5c	Role of study sponsor and funders, if any, in study design; collection, management, analysis, and interpretation of data; writing of the report; and the decision to submit the report for publication, including whether they will have ultimate authority over any of these activities	2
	5d	Composition, roles, and responsibilities of the coordinating centre, steering committee, endpoint adjudication committee, data management team, and other individuals or groups overseeing the trial, if applicable (see Item 21a for data monitoring committee)	10-11, 13

For peer review only

For peer review only - http://bmjopen.bmj.com/site/about/guidelines.xhtml

Introduction

Background and rationale	6a	Description of research question and justification for undertaking the trial, including summary of relevant studies (published and unpublished) examining benefits and harms for each intervention	4
	6b	Explanation for choice of comparators	5, 7
Objectives	7	Specific objectives or hypotheses	4-5
Trial design	8	Description of trial design including type of trial (eg, parallel group, crossover, factorial, single group), allocation ratio, and framework (eg, superiority, equivalence, noninferiority, exploratory)	5

Methods: Participants, interventions, and outcomes

Study setting	9	Description of study settings (eg, community clinic, academic hospital) and list of countries where data will be collected. Reference to where list of study sites can be obtained	5-6
Eligibility criteria	10	Inclusion and exclusion criteria for participants. If applicable, eligibility criteria for study centres and individuals who will perform the interventions (eg, surgeons, psychotherapists)	6
Interventions	11a	Interventions for each group with sufficient detail to allow replication, including how and when they will be administered	5
	11b	Criteria for discontinuing or modifying allocated interventions for a given trial participant (eg, drug dose change in response to harms, participant request, or improving/worsening disease)	11
	11c	Strategies to improve adherence to intervention protocols, and any procedures for monitoring adherence (eg, drug tablet return, laboratory tests)	9-11
	11d	Relevant concomitant care and interventions that are permitted or prohibited during the trial	7
Outcomes	12	Primary, secondary, and other outcomes, including the specific measurement variable (eg, systolic blood pressure), analysis metric (eg, change from baseline, final value, time to event), method of aggregation (eg, median, proportion), and time point for each outcome. Explanation of the clinical relevance of chosen efficacy and harm outcomes is strongly recommended	7-10
Participant timeline	13	Time schedule of enrolment, interventions (including any run-ins and washouts), assessments, and visits for participants. A schematic diagram is highly recommended (see Figure)	14

For peer review only - http://bmjopen.bmj.com/site/about/guidelines.xhtml

Sample size	14	Estimated number of participants needed to achieve study objectives and how it was determined, including clinical and statistical assumptions supporting any sample size calculations	11
Recruitment	15	Strategies for achieving adequate participant enrolment to reach target sample size	8, 11
**Methods: Assignment of interventions (for controlled trials)**			
Allocation:			
Sequence generation	16a	Method of generating the allocation sequence (eg, computer-generated random numbers), and list of any factors for stratification. To reduce predictability of a random sequence, details of any planned restriction (eg, blocking) should be provided in a separate document that is unavailable to those who enrol participants or assign interventions	7
Allocation concealment mechanism	16b	Mechanism of implementing the allocation sequence (eg, central telephone; sequentially numbered, opaque, sealed envelopes), describing any steps to conceal the sequence until interventions are assigned	7
Implementation	16c	Who will generate the allocation sequence, who will enrol participants, and who will assign participants to interventions	7
Blinding (masking)	17a	Who will be blinded after assignment to interventions (eg, trial participants, care providers, outcome assessors, data analysts), and how	7
	17b	If blinded, circumstances under which unblinding is permissible, and procedure for revealing a participant's allocated intervention during the trial	~
**Methods: Data collection, management, and analysis**			
Data collection methods	18a	Plans for assessment and collection of outcome, baseline, and other trial data, including any related processes to promote data quality (eg, duplicate measurements, training of assessors) and a description of study instruments (eg, questionnaires, laboratory tests) along with their reliability and validity, if known. Reference to where data collection forms can be found, if not in the protocol	7-10
	18b	Plans to promote participant retention and complete follow-up, including list of any outcome data to be collected for participants who discontinue or deviate from intervention protocols	7-10

For peer review only - http://bmjopen.bmj.com/site/about/guidelines.xhtml

Data management	19	Plans for data entry, coding, security, and storage, including any related processes to promote data quality (eg, double data entry; range checks for data values). Reference to where details of data management procedures can be found, if not in the protocol	10-11
Statistical methods	20a	Statistical methods for analysing primary and secondary outcomes. Reference to where other details of the statistical analysis plan can be found, if not in the protocol	11-13
	20b	Methods for any additional analyses (eg, subgroup and adjusted analyses)	11-13
	20c	Definition of analysis population relating to protocol non-adherence (eg, as randomised analysis), and any statistical methods to handle missing data (eg, multiple imputation)	11-13
**Methods: Monitoring**			
Data monitoring	21a	Composition of data monitoring committee (DMC); summary of its role and reporting structure; statement of whether it is independent from the sponsor and competing interests; and reference to where further details about its charter can be found, if not in the protocol. Alternatively, an explanation of why a DMC is not needed	10-11
	21b	Description of any interim analyses and stopping guidelines, including who will have access to these interim results and make the final decision to terminate the trial	11-13
Harms	22	Plans for collecting, assessing, reporting, and managing solicited and spontaneously reported adverse events and other unintended effects of trial interventions or trial conduct	11
Auditing	23	Frequency and procedures for auditing trial conduct, if any, and whether the process will be independent from investigators and the sponsor	11
**Ethics and dissemination**			
Research ethics approval	24	Plans for seeking research ethics committee/institutional review board (REC/IRB) approval	13
Protocol amendments	25	Plans for communicating important protocol modifications (eg, changes to eligibility criteria, outcomes, analyses) to relevant parties (eg, investigators, REC/IRBs, trial participants, trial registries, journals, regulators)	10-11

For peer review only - http://bmjopen.bmj.com/site/about/guidelines.xhtml

Consent or assent	26a	Who will obtain informed consent or assent from potential trial participants or authorised surrogates, and how (see Item 32)	6
	26b	Additional consent provisions for collection and use of participant data and biological specimens in ancillary studies, if applicable	~
Confidentiality	27	How personal information about potential and enrolled participants will be collected, shared, and maintained in order to protect confidentiality before, during, and after the trial	10
Declaration of interests	28	Financial and other competing interests for principal investigators for the overall trial and each study site	13
Access to data	29	Statement of who will have access to the final trial dataset, and disclosure of contractual agreements that limit such access for investigators	14
Ancillary and post-trial care	30	Provisions, if any, for ancillary and post-trial care, and for compensation to those who suffer harm from trial participation	~
Dissemination policy	31a	Plans for investigators and sponsor to communicate trial results to participants, healthcare professionals, the public, and other relevant groups (eg, via publication, reporting in results databases, or other data sharing arrangements), including any publication restrictions	13
	31b	Authorship eligibility guidelines and any intended use of professional writers	13
	31c	Plans, if any, for granting public access to the full protocol, participant-level dataset, and statistical code	13
**Appendices**			
Informed consent materials	32	Model consent form and other related documentation given to participants and authorised surrogates	~
Biological specimens	33	Plans for collection, laboratory evaluation, and storage of biological specimens for genetic or molecular analysis in the current trial and for future use in ancillary studies, if applicable	~

*It is strongly recommended that this checklist be read in conjunction with the SPIRIT 2013 Explanation & Elaboration for important clarification on the items. Amendments to the protocol should be tracked and dated. The SPIRIT checklist is copyrighted by the SPIRIT Group under the Creative Commons "Attribution-NonCommercial-NoDerivs 3.0 Unported" license.

For peer review only - http://bmjopen.bmj.com/site/about/guidelines.xhtml
